# Supplementary material for: Jingmen Tick Virus in Ticks from Kenya
Source: Viruses. 2022 May 13;14(5):1041. doi: 10.3390/v14051041 (PMC9147648; doi:10.3390/v14051041)
Supplement: Supplementary file 1 [file viruses-14-01041-s001.zip › viruses-1707077-supplementary/Figure S1.pdf]

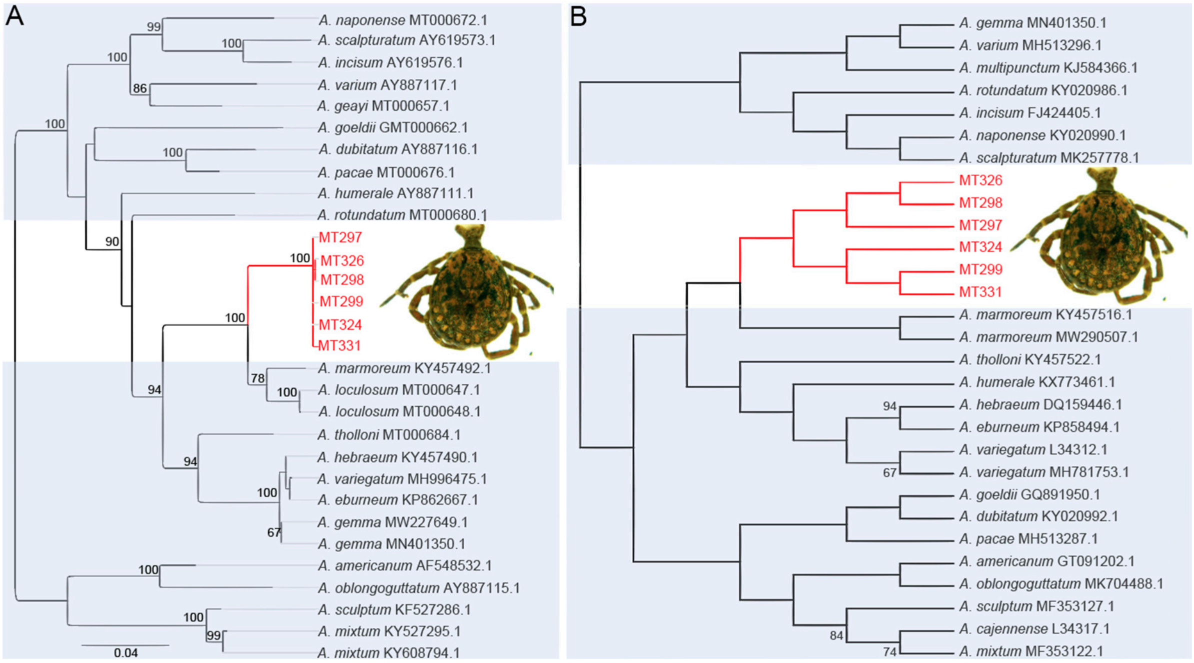

Figure S1. Phylogenetic relationship of *Amblyomma* ticks collected from tortoises (that could not be identified to species level using morphological keys), and other *Amblyomma* congeners. The maximum likelihood phylogenetic trees were based on (**A**) ITS2 (1,008 bp) and (**B**) 16S rRNA (402 bp). Phylogenetic analyses for ITS2 was performed in PhyML v. 2.2.4 with General-time-reversible (GTR) substitution models over 1000 bootstrap replicates. For 16S rRNA, phylogenetic analyses was performed in MEGA-X with Tamura-Nei model applying 1000 bootstrap replicates. Only bootstrap values exceeding 65% are shown. The identified sequences are highlighed in red.
