# Supplementary material for: Jingmen Tick Virus in Ticks from Kenya
Source: Viruses. 2022 May 13;14(5):1041. doi: 10.3390/v14051041 (PMC9147648; doi:10.3390/v14051041)
Supplement: Supplementary file 1 [file viruses-14-01041-s001.zip › viruses-1707077-supplementary/Figure S3.pdf]

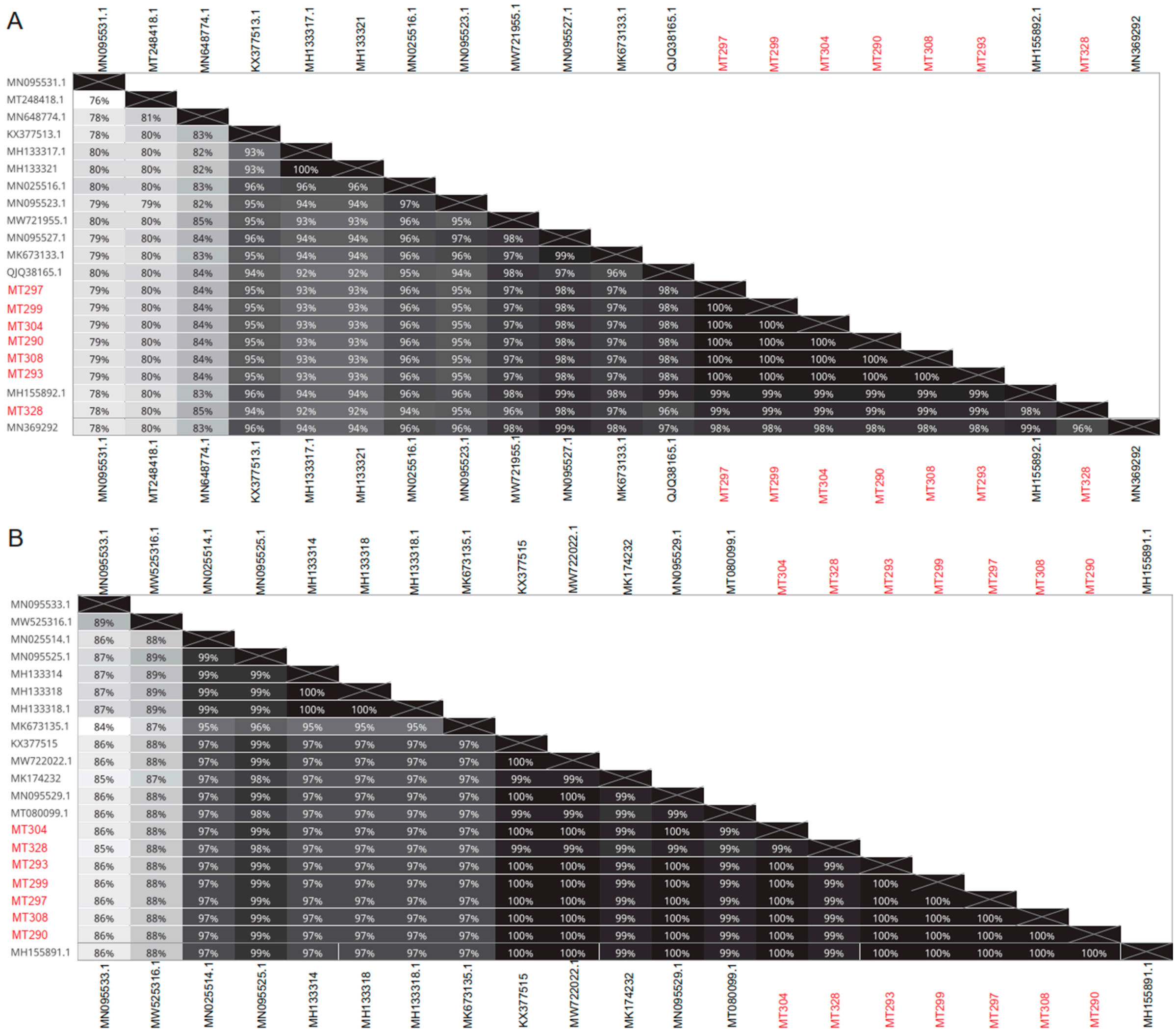

Figure S3. Jingmenviruses distance matrixes. (**A**) RNA-dependent RNA polymerase gene of selected Jingmenviruses and viruses sequenced in the present study; (**B**) Protease/helicase protein of selected Jingmenviruses and viruses sequenced in the present study. Light grey to dark shades highlights amino acid distances. The viruses sequenced in the present study are shown in red.
