# Supplementary material for: Jingmen Tick Virus in Ticks from Kenya
Source: Viruses. 2022 May 13;14(5):1041. doi: 10.3390/v14051041 (PMC9147648; doi:10.3390/v14051041)
Supplement: Supplementary file 1 [file viruses-14-01041-s001.zip › viruses-1707077-supplementary/Table S1.pdf]

Table S1. Primers, probes and PCR conditions used in the study.

Primer or Probe Name	Sequence (5'to 3')	Amplicon Size (nt)	Annealing Temperature ℃	Note
Tick COI F	TAAACTTCTGGATGTCCAAAAAATCA	658	55	Tick identification [1]
Tick COI R	ATTCAACCAATCATAAAGATATTGG			
F-RMI16S	AATTGCTGTAGTATTTTGAC	450	55	Tick identification [2]
R-RMI16S	TCTGAACTCAGATCAAGTAG			
TITS2F1	CGAGACTTGGTGTGAATTGCA	750-2000	65	Tick identification [3]
TITS2R1	TCCCATACACCACATTTCCCG			
JMTV-b_F1	AGYGUGGCRGGACAGGG	616	64 - 56	JMTV screening
JMTV-b_R1	GCCAGUACCURUGGGAGC			
JMTV_115_F	GAGGCGTAAACCCTAGCCTC	117	60	Quantitative TaqMan Real-time PCR
JMTV_231_R	GACTGGCCCCGACTGATAAG			
JMTV_118_Probe	CGCCTGTTTCCCCTCTCATC			

References

1. Lv, J.; Wu, S.; Zhang, Y.; Chen, Y.; Feng, C.; Yuan, X.; Jia, G.; Deng, J.; Wang, C.; Wang, Q.; Mei, L.; Lin, X. Assessment of Four DNA Fragments (COI, 16S RDNA, ITS2, 12S rDNA) for Species Identification of the Ixodida (Acari: Ixodida). *Parasites and Vectors* **2014**, *7*, 1–11. https://doi.org/10.1186/1756-3305-7-93/TABLES/4.
2. Brahma, R. K.; Dixit, V.; Sangwan, A. K.; Doley, R. Identification and Characterization of Rhipicephalus (Boophilus) Microplus and Haemaphysalis Bispinosa Ticks (Acari: Ixodidae) of Northeast India by ITS2 and 16S RDNA Sequences and Morphological Analysis. *Exp. Appl. Acarol.* **2014**, *62*, 253–265. https://doi.org/10.1007/S10493-013-9732-4.
3. Chitimia, L.; Lin, R. Q.; Cosoroaba, I.; Braila, P.; Song, H. Q.; Zhu, X. Q. Molecular Characterization of Hard and Soft Ticks from Romania by Sequences of the Internal Transcribed Spacers of Ribosomal DNA. *Parasitol. Res.* **2009**, *105*, 907–911. https://doi.org/10.1007/S00436-009-1474-1.
