# Supplementary material for: Jingmen Tick Virus in Ticks from Kenya
Source: Viruses. 2022 May 13;14(5):1041. doi: 10.3390/v14051041 (PMC9147648; doi:10.3390/v14051041)
Supplement: Supplementary file 1 [file viruses-14-01041-s001.zip › viruses-1707077-supplementary/Table S2.pdf]

Table S2. Sequences generated in this study submitted to GenBank.

>MT326_ON220154
TCCTAATCCAACATCGAGGTCGCAAACTATTTTGTCTATAAGAACTATCAAAAATTATTACGCTGTTA
TCCCTAGAGTATTTTTCCATATGACCGTTAATAATGGTTCGTATTTTTTTTTAAATAAAGTTTAATAT
ATTTATTAATCGCCCCAATTAAAAATAAATAATTATAAATTTAAATTATTTTTTAAAAAATTCTTAGG
GTCTTCTTGTCCTTAATTTTTATTGTTGTTTCTTCACAAACAAAAATAACTTTAAATTTTAATTTTAA
AAAAGTAGTTTTCTGTGGGTCCATTCTCTTAGCACTC

>MT324_ON220155
TCCTAATCCAACATCGAGGTCGCAAACTATTTTGTCTATAAGAACTATCAAAAATTATTACGCTGTTA
TCCCTAGAGTATTTTTCCATATGACCATTAATAATGGTTCATATTTTTTTTTAAATAAAGTTTAATAT
ATTTATTAATCGCCCCAATTAAAAATAAATAATTATAAATTTAAATTATCTTTTAAAAAATTCTTAGG
GTCTTCTTGTCCTTAATTTTTATTGTTGTTTCTTCACAAACAAAAATAACTTTAAATTTTAATTTTAA
AAAAGTAGTTTTCTGTGGGTCCATTCTCTTAGCACTC

>MT298_ON220156
TCCTAATCCAACATCGAGGTCGCAAACTATTTTGTCTATAAGAACTATCAAAAATTATTACGCTGTTA
TCCCTAGAGTATTTTTCCATATGACCATTAATAATGGTTCGTATTTTTTTTTAAATAAAGTTTAATAT
ATTTATTAATCGCCCCAATTAAAAATAAATAATTATAAATTTAAATTATCTTTTAAAAAATTCTTAGG
GTCTTCTTGTCCTTAATTTTTATTGTTGTTTCTTCACAAACAAAAATAACTTTAAATTTTAATTTTAA
AAAAGTAGTTTTCTGTGGGTCCATTCTCTTAGCACTC

>MT299_ON220157
TCCTAATCCAACATCGAGGTCGCAAACTATTTTGTCTATAAGAACTATCAAAAATTATTACGCTGTTA
TCCCTAGAGTATTTTTCCATATGACCATTAATAATGGTTCGTATTTTTTTTTAAATAAAGTTTAATAT
ATTTATTAATCGCCCCAATTAAAAATAAATAATTATAAATTTAAATTATCTTTTAAAAAATTCTTAGG
GTCTTCTTGTCCTTAATTTTTATTGTTGTTTCTTCACAAACAAAAATAACTTTAAATTTTAATTTTAA
AAAAGTAGTTTTCTGTGGGTCCATTCTCTTAGCACTC

>MT331_ON220158
TCCTAATCCAACATCGAGGTCGCAAACTATTTTGTCTATAAGAACTATCAAAAATTATTACGCTGTTA
TCCCTAGAGTATTTTTCCATATGACCATTAATAATGGTTCGTATTTTTTTTTAAATAAAGTTTAATAT
ATTTATTAATCGCCCCAATTAAAAATAAATAATTATAAATTTAAATTATCTTTTAAAAAATTCTTAGG
GTCTTCTTGTCCTTAATTTTTATTGTTGTTTCTTCACAAACAAAAATAACTTTAAATTTTAATTTTAA
AAAAGTAGTTTTCTGTGGGTCCATTCTCTTAGCACTC

>MT297_ON220159
TCCTAATCCAACATCGAGGTCGCAAACTATTTTGTCTATAAGAACTATCAAAAATTATTACGCTGTTA
TCCCTAGAGTATTTTTCCATATGACCATTAATAATGGTTCGTATTTTTTTTTAAATAAAGTTTAATAT
ATTTATTAATCGCCCCAATTAAAAATAAATAATTATAAATTTAAATTATCTTTTAAAAAATTCTTAGG
GTCTTCTTGTCCTTAATTTTTATTGTTGTTTCTTCACAAACAAAAATAACTTTAAATTTTAATTTTAA
AAAAGTAGTTTTCTGTGGGTCCATTCTCTTAGCACTC

>MT326_ON212401
CGGCAAAACGGACGGCTCAAAGGAACGCTGAAGATCCCTCGAAAGACTTTGCGAAAGAGGAGGAGAGT
GGGTTGAACCCTTCGGCCGCGCGAAAGCGGCAGAGTCGGGTCGCCCTGCGCTGACGTAACGTGGTCCA
GTGGCTGTCGAGCCATATAACGTCCAGTCTCTGAATGGCGACCGCCGCAGGAAGGGATTACCTGCAGG

GAAAGAGCGGTCCCAAGGAGAGAAGAGGCACGGGGCGGGTGGCAAAGCGCTGCCTGCTGGACCGCGGA
GAAGTATCCAAACAGCTGCGAACCGCAGAGACGGGAGCGCCCTGTCCAGGCACGCCTGCGCGAGAAGA
AGGGAGATCTCGCGCGGGAAGGTGACGGGCGTGAACGCGGTGAGCGCGGCAGTTCGGAGGCGCAGTGG
CGCGTCGCGACGCGGCAAAGAAGGGATCCTCCGGACCAGAGGAAAAATGGTCCGCCTCCCTGAATGGG
CGCGAAGTCGAGTACAGGTTGAACGGATGCAGTTTCGAGCGCAGTCCGCCGTCGGTCTAAGTGCTTCG
CAGTCTCTGCCCCGATAAAAACTGGGCCACTCCAGTTGGGGCGGGGGCGACGCAAAACTGTTGACGAT
GCCCCTCCGCCAGGTCGAGGCCGTCCTCTCGAGGGAAAGAGGGACGGCAGGGAATTATTCGGCCGCGG
CGGTCGGGGGCCCTACTCAACGCTTGTTATCGCGCAAACGCAGGCGCGCTGTCCTGAGCGCCGGCCAG
CGGTGCCTTCCTGTCTCGCTCCAGTCGCCGAGGGTGTTGCCGGCTGAGAGCGGGGAATGGGCGGAAGG
TGTGGGCCGGAGATGGGGAAGGACACACGCGCGCTCGACCGCGCTGGAAACGAGCGAGAGAATGCATA
TCTGTCGACCTCAGATCAGGCGAGACAACCCGCTGAATTTAAGCATATCACTAAGCGGAGGAAAAGAA
ACCAACA

>MT298_ON212402
CGGCAAAACGGACGGCTCAAAGGAACGCTGAAGATCCCTCGAAAGACTTTGCGAAAGAGGAGGAGAGT
GGGTTGAACCCTTCGGCCGCGCGAAAGCGGCAGAGTCGGGTCGCCCTGCGCTGACGTAACGTGGTCCA
GTGGCTGTCGAGCCATATAACGTCCAGTCTCTGAATGGCGACCGCCGCAGGAAGGGATTACCTGCAGG
GAAAGAGCGGTCCCAAGGAGAGAAGAGGCACGGGGCGGGTGGCAAAGCGCTGCCTGCTGGACCGCGGA
GAAGTATCCAAACAGCTGCGAACCGCAGAGACGGGAGCGCCCTGTCCAGGCACGCCTGCGCGAGAAGA
AGGGAGATCTCGCGCGGGAAGGTGACGGGCGTGAACGCGGTGAGCGCGGCAGTTCGGAGGCGCAGTGG
CGCGTCGCGACGCGGCAAAGAAGGGATCCTCCGGACCAGAGGAAAAATGGTCCGCCTCCCTGAATGGG
CGCGAAGTCGAGTACAGGTTGAACGGATGCAGTTTCGAGCGCAGTCCGCCGTCGGTCTAAGTGCTTCG
CAGTCTCTGCCCCGATAAAAACTGGGCCACTCCAGTTGGGGCGGGGGCGACGCAAAACTGTTGACGAT
GCCCCTCCGCCAGGTCGAGGCCGTCCTCTCGAGGGAAAGAGGGACGGCAGGGAATTATTCGGCCGCGG
CGGTCGGGGGCCCTACTCAACGCTTGTTATCGCGCAAACGCAGGCGCGCTGTCCTGAGCGCCGGCCAG
CGGTGCCTTCCTGTCTCGCTCCAGTCGCCGAGGGTGTTGCCGGCTGAGAGCGGGGAATGGGCGGAAGG
TGTGGGCCGGAGATGGGGAAGGACACACGCGCGCTCGACCGCGCTGGAAACGAGCGAGAGAATGCATA
TCTGTCGACCTCAGATCAGGCGAGACAACCCGCTGAATTTAAGCATATCACTAAGCGGAGGAAAAGAA
ACCAACA

>MT297_ON212403
GGCAAAACGGACGGCTCAAAGGAACGCTGAAGATCCCTCGAAAGACTTTGCGAAAGAGGAGGAGAGTG
GGTTGAACCCTTCGGCCGCGCGAAAGCGGCAGAGTCGGGTCGCCCTGCGCTGACGTAACGTGGTCCAG
TGGCTGTCGAGCCATATAACGTCCAGTCTCTGAATGGCGACCGCCGCAGGAAGGGATTACCTGCAGGG
AAAGAGCGGTCCCAAGGAGAGAAGAGGCACGGGGCGGGTGGCAAAGCGCTGCCTGCTGGACCGCGGAG
AAGTATCCAAACAGCTGCGAACCGCAGAGACGGGAGCGCCCTGTCCAGGCACGCCTGCGCGAGAAGAA
GGGAGATCTCGCGCGGGAAGGTGACGGGCGTGAACGCGGTGAGCGCGGCAGTTCGGAGGCGCAGTGGC
GCGTCGCGACGCGGCAAAGAAGGGATCCTCCGGACCAGAGGAAAAATGGTCCGCCTCCCTGAATGGGC
GCGAAGTCGAGTACAGGTTGAACGGATGCAGTTTCGAGCGCAGTCCGCCGTCGGTCTAAGTGCTTCGC
AGTCTCTGCCCCGATAAAAACTGGGCCACTCCAGTTGGGGCGGGGGCGACGCAAAACTGTTGACGATG
CCCCTCCGCCAGGTCGAGGCCGTCCTCTCGAGGGAAAGAGGGACGGCAGGGAATTATTCGGCCGCGGC
GGTCGGGGGCCCTACTCAACGCTTGTTACCGCGCAAACGCAGGCGCGCTGTCCTGAGCGCCGGCCAGC
GGTGCCTTCCTGTCTCGCTCCAGTCGCCGAGGGTGTTGCCGGCTGAGAGCGGGGAATGGGCGGAAGGT
GTGGGCCGGAGATGGGGAAGGACACACGCGCGCTCGACCGCGCTGGAAACGAGCGAGAGAATGCATAT
CTGTCGACCTCAGATCAGGCGAGACAACCCGCTGAATTTAAGCATATCACTAAGCGGAGGAAAAGAAA
CCAACA

>MT299_ON212404

CGGCAAAACGGACGGCTCAAAGGAACGCTGAAGATCCCTCGAAAGACTTTGCGAAAGAGGAGGAGAGT
GGGTTGAACCCTTCGGCCGCGCGAAAGCGGCAGAGTCGGGTCGCCCTGCGCTGACGTAACGTGGTCCA
GTGGCTGTCGAGCCATATAACGTCCAGTCTCTGAATGGCGACCGCCGCAGGAAGGGATTACCTGCAGG
GAAAGAGCGGTCCCAAGGAGAGAAGAGGCACGGGGCGGGTGGCAAAGCGCTGCCTGCTGGACCGCGGA
GAAGTATCCAAACAGCTGCGAACCGCAGAGACGGGAGCGCCCTGTCCAGGCACGCCTGCGCGAGAAGA
AGGGAGATCTCGCGCGGGAAGGTGACGGGCGTGAACGCGGTGAGCGCGGCAGTTCGGAGGCGCAGTGG
CGCGTCGCGACGCGGCAAAGAAGGGATCCTCCGGACCAGAGGAAAAATGGTCCGCCTCCCTGAATGGG
CGCGAAGTCGAGTACAGGTTGAACGGATGCAGTTTCGAGCGCAGTCCGCCGTCGGTCTAAGTGCTTCG
CAGTCTCTGCCCCGATAAAAACTGGGCCACTCCAGTTGGGGCGGGGGCGACGCAAAACTGTTGACGAT
GCCCCTCCGCCAGGTCGAGGCCGTCCTCTCGAGGGAAAGAGGGACGGCAGGGAATTATTCGGCCGCGG
CGGTCGGGGGCCCTACTCAACGCTTGTTAYCGCGCAAACGCAGGCGCGCTGTCCTGAGCGCCGGCCAG
CGGTGCCTTCCTGTCTCGCTCCAGTCGCCGAGGGTGTTGCCGGCTGAGAGCGGGGAATGGGCGGAAGG
TGTGGGCCGGAGATGGGGAAGGACACACGCGCGCTCGACCGCGCTGGAAACGAGCGAGAGAATGCATA
TCTGTCGACCTCAGATCAGGCGAGACAACCCGCTGAATTTAAGCATATCACTAAGCGGAGGAAAAGAA
ACCAACA

>MT324_ON212405
GGCAAAACGGACGGCTCAAAGGAACGCTGAAGATCCCTCGAAAGACTTTGCGAAAGAGGAGGAGAGTG
GGTTGAACCCTTCGGCCGCGCGAAAGCGGCAGAGTCGGGTCGCCCTGCGCTGACGTAACGTGGTCCAG
TGGCTGTCGAGCCATATAACGTCCAGTCTCTGAATGGCGACCGCCGCAGGAAGGGATTACCTGCAGGG
AAAGAGCGGTCCCAAGGAGAGAAGAGGCACGGGGCGGGTGGCAAAGCGCTGCCTGCTGGACCGCGGAG
AAGTATCCAAACAGCTGCGAACCGCAGAGACGGGAGCGCCCTGTCCAGGCACGCCTGCGCGAGAAGAA
GGGAGATCTCGCGCGGGAAGGTGACGGGCGTGAACGCGGTGAGCGCGGCAGTTCGGAGGCGCAGTGGC
GCGTCGCGACGCGGCAAAGAAGGGATCCTCCGGACCAGAGGAAAAATGGTCCGCCTCCCTGAATGGGC
GCGAAGTCGAGTACAGGTTGAACGGATGCAGTTTCGAGCGCAGTCCGCCGTCGGTCTAAGTGCTTCGC
AGTCTCTGCCCCGATAAAAACTGGGCCACTCCAGTTGGGGCGGGGGCGACGCAAAACTGTTGACGATG
CCCCTCCGCCAGGTCGAGGCCGTCCTCTCGAGGGAAAGAGGGACGGCAGGGAATTATTCGGCCGCGGC
GGTCGGGGGCCCTACTCAACGCTTGTTACCGCGCAAACGCAGGCGCGCTGTCCTGAGCGCCGGCCAGC
GGTGCCTTCCTGTCTCGCTCCAGTCGCCGAGGGTGTTGCCGGCTGAGAGCGGGGAATGGGCGGAAGGT
GTGGGCCGGAGATGGGGAAGGACACACGCGCGCTCGACCGCGCTGGAAACGAGCGAGAGAATGCATAT
CTGTCGACCTCAGATCAGGCGAGACAACCCGCTGAATTTAAGCATATCACTAAGCGGAGGAAAAGAAA
CCAACA

>T3_ON158817
GAGATCGAGTAAAGAAGTCAATGGACGCTACCGGCGCCGCTATAGCGGAAGACGTGGCGGGCTGGGAC
ACAAAGATCTCCAAAGGACTGCTCACCCTGGAGTCCCACATGTTCACCAAGCTGGCGGAGGATGAAGA
AATGGCGCGGGAGATCCACCACCTCTACCGACTCTACGCAGATCCACACATGGTGGTCCAGAGGGAGA
TTGAAGGTGAAGTTCATGACGTACTCCTTAGGGGGAGGGGGCAGGTCTCTTCCGGGAGGCAGCCCACC
TACGCGGCCAACACCATCACCAACTTCATAACGACAACATATGGAATGGCCGTGACCCTTGGTATACC
TGAGGCCGACTGGCCCCGACTGGTAAGGGACCTCACAGATGAGAGGGGAAACAGGCGTCTCCTCGTCT
CGGGGGACGACAAGGTTCTTTTCTTAAGAGGAGATGAGGCTAGGGCTTATGCCTCATCGGCCTACCGG
ATAAGTAACGAC

>MT335_ON158818
GAGATCGAGTAAAGAAGTCAATGGACGCTACCGGCGCCGCTATAGCGGAAGACGTGGCGGGCTGGGAC
ACAAAGATCTCCAAAGGACTGCTCACCCTGGAGTCCCACATGTTCACCAAGCTGGCGGAGGATGAAGA
AATGGCGCGGGAGATCCACCACCTCTACCGACTCTACGCAGATCCACACATGGTGGTCCAGAGGGAGA
TTGAAGGTGAAGTTCATGACGTACTCCTTAGGGGGAGGGGGCAGGTCTCTTCCGGGAGGCAGCCCACC

TACGCGGCCAACACCATCACCAACTTCATAACGACAACATATGGAATGGCCGTGACCCTTGGTATACC
TGAGGCCGACTGGCCCCGACTGGTAAGGGACCTCACAGATGAGAGGGGAAACAGGCGTCTCCTCGTCT
CGGGGGACGACAAGGTTCTTTTCTTAAGAGGAGATGAGGCTAGGGCTTATGCCTCATCGGCCTACCGG
ATAAGTAACGAC

>MT334_ON158819
GAGATCGAGTAAAGAAGTCAATGGACGCTACCGGCGCCGCTATAGCGGAAGACGTGGCGGGCTGGGAC
ACAAAGATCTCCAAAGGACTGCTCACCCTGGAGTCCCACATGTTCACCAAGCTGGCGGAGGATGAAGA
AATGGCGCGGGAGATCCACCACCTCTACCGACTCTACGCAGATCCACACATGGTGGTCCAGAGGGAGA
TTGAAGGTGAAGTTCATGACGTACTCCTTAGGGGGAGGGGGCAGGTCTCTTCCGGGAGGCAGCCCACC
TACGCGGCCAACACCATCACCAACTTCATAACGACAACATATGGAATGGCCGTGACCCTTGGTATACC
TGAGGCCGACTGGCCCCGACTGGTAAGGGACCTCACAGATGAGAGGGGAAACAGGCGTCTCCTCGTCT
CGGGGGACGACAAGGTTCTTTTCTTAAGAGGAGATGAGGCTAGGGCTTATGCCTCATCGGCCTACCGG
ATAAGTAACGAC

>MT328_ON158820
GAGATCGAGTAAAGAAGTCAATGGACGCTACCGGCGCCGCTATAGCGGAAGACGTGGCGGGCTGGGAC
ACAAAGATCTCCAAAGGACTGCTCACCCTGGAGTCCCACATGTTCACCAAGCTGGCGGAGGATGAAGA
AATGGCGCGGGAGATCCACCACCTCTACCGACTCTACGCAGATCCACACATGGTGGTCCAGAGGGAGA
TTGAAGGTGAAGTTCATGACGTACTCCTTAGGGGGAGGGGGCAGGTCTCTTCCGGGAGGCAGCCCACC
TACGCGGCCAACACCATCACCAACTTCATAACGACAACATATGGAATGGCCGTGACCCTTGGTATACC
TGAGGCCGACTGGCCCCGACTGGTAAGGGACCTCACAGATGAGAGGGGAAACAGGCGTCTCCTCGTCT
CGGGGGACGACAAGGTTCTTTTCTTAAGAGGAGATGAGGCTAGGGCTTATGCCTCATCGGCCTACCGG
ATAAGTAACGAC

>MT323_ON158821
GAGATCGAGTAAAGAAGTCAATGGACGCTACCGGCGCCGCTATAGCGGAAGACGTGGCGGGCTGGGAC
ACAAAGATCTCCAAAGGACTGCTCACCCTGGAGTCCCACATGTTCACCAAGCTGGCGGAGGATGAAGA
AATGGCGCGGGAGATCCACCACCTCTACCGACTCTACGCAGATCCACACATGGTGGTCCAGAGGGAGA
TTGAAGGTGAAGTTCATGACGTACTCCTTAGGGGGAGGGGGCAGGTCTCTTCCGGGAGGCAGCCCACC
TACGCGGCCAACACCATCACCAACTTCATAACGACAACATATGGAATGGCCGTGACCCTTGGTATACC
TGAGGCCGACTGGCCCCGACTGGTAAGGGACCTCACAGATGAGAGGGGAAACAGGCGTCTCCTCGTCT
CGGGGGACGACAAGGTTCTTTTCTTAAGAGGAGATGAGGCTAGGGCTTATGCCTCATCGGCCTACCGG
ATAAGTAACGAC

>MT299_ON158822
GAGATCGAGTAAAGAAGTCAATGGACGCTACCGGCGCCGCTATAGCGGAAGACGTGGCGGGCTGGGAC
ACAAAGATCTCCAAAGGACTGCTCACCCTGGAGTCCCACATGTTCACCAAGCTGGCGGAGGATGAAGA
AATGGCGCGGGAGATCCACCACCTCTACCGACTCTACGCAGATCCACACATGGTGGTCCAGAGGGAGA
TTGAAGGTGAAGTTCATGACGTACTCCTTAGGGGGAGGGGGCAGGTCTCTTCCGGGAGGCAGCCCACC
TACGCGGCCAACACCATCACCAACTTCATAACGACAACATATGGAATGGCCGTGACCCTTGGTATACC
TGAGGCCGACTGGCCCCGACTGGTAAGGGACCTCACAGATGAGAGGGGAAACAGGCGTCTCCTCGTCT
CGGGGGACGACAAGGTTCTTTTCTTAAGAGGAGATGAGGCTAGGGCTTATGCCTCATCGGCCTACCGG
ATAAGTAACGAC

>MT331_ON158823
GAGATCGAGTAAAGAAGTCAATGGACGCTACCGGCGCCGCTATAGCGGAAGACGTGGCGGGCTGGGAC
ACAAAGATCTCCAAAGGACTGCTCACCCTGGAGTCCCACATGTTCACCAAGCTGGCGGAGGATGAAGA

AATGGCGCGGGAGATCCACCACCTCTACCGACTCTACGCAGATCCACACATGGTGGTCCAGAGGGAGA
TTGAAGGTGAAGTTCATGACGTACTCCTTAGGGGGAGGGGGCAGGTCTCTTCCGGGAGGCAGCCCACC
TACGCGGCCAACACCATCACCAACTTCATAACGACAACATATGGAATGGCCGTGACCCTTGGTATACC
TGAGGCCGACTGGCCCCGACTGGTAAGGGACCTCACAGATGAGAGGGGAAACAGGCGTCTCCTCGTCT
CGGGGGACGACAAGGTTCTTTTCTTAAGAGGAGATGAGGCTAGGGCTTATGCCTCATCGGCCTACCGG
ATAAGTAACGAC

>MT332_ON158824
GAGATCGAGTAAAGAAGTCAATGGACGCTACCGGCGCCGCTATAGCGGAAGACGTGGCGGGCTGGGAC
ACAAAGATCTCCAAAGGACTGCTCACCCTGGAGTCCCACATGTTCACCAAGCTGGCGGAGGATGAAGA
AATGGCGCGGGAGATCCACCACCTCTACCGACTCTACGCAGATCCACACATGGTGGTCCAGAGGGAGA
TTGAAGGTGAAGTTCATGACGTACTCCTTAGGGGGAGGGGGCAGGTCTCTTCCGGGAGGCAGCCCACC
TACGCGGCCAACACCATCACCAACTTCATAACGACAACATATGGAATGGCCGTGACCCTTGGTATACC
TGAGGCCGACTGGCCCCGACTGGTAAGGGACCTCACAGATGAGAGGGGAAACAGGCGTCTCCTCGTCT
CGGGGGACGACAAGGTTCTTTTCTTAAGAGGAGATGAGGCTAGGGCTTATGCCTCATCGGCCTACCGG
ATAAGCAACGAC

>MT333_ON158825
GAGATCGAGTAAAGAAGTCAATGGACGCTACCGGCGCCGCTATAGCGGAAGACGTGGCGGGCTGGGAC
ACAAAGATCTCCAAAGGACTGCTCACCCCGGAGTCCCACATGTTCACCAAGCCGGCGGAGGATGAAGA
AATGGCGCGGGAGATCCACCACCTCTACCGACTCTACGCAGATCCACACATGGTGGTCCAGAGGGAGA
TTGAAGGTGAAGTTCATGACGTACTCCTTAGGGGGAGGGGGCAGGTCTCTTCCGGGAGGCAGCCCACC
TACGCGGCCAACACCATCACCAACTTCATAACGACAACATATGGAATGGCCGTGACCCTTGGTATACC
TGAGGCCGACTGGCCCCGACTGGTAAGGGACCTCACAGATGAGAGGGGAAACAGGCGTCTCCTCGTCT
CGGGGGACGACAAGGTTCTTTTCTTAAGAGGAGATGAGGCTAGGGCTTATGCCTCATCGGCCTACCGG
ATAAGTAACGAC

>MT330_ON158826
GAGATCGAGTAAAGAAGTCAATGGACGCAACCGGCGCCGCTATAGCGGAAGACGTGGCGGGCTGGGAC
ACAAAGATCTCCAAAGGACTGCTCACCCCGGAGTCCCACATGTTCACCAAGCCGGCGGAGGATGAAGA
AATGGCGCGGGAGATCCACCACCTCTACCGACTCTACGCAGATCCACACATGGTGGTCCAGAGGGAGA
TTGAAGGTGAAGTTCATGACGTACTCCTTAGGGGGAGGGGGCAGGTCTCTTCCGGGAGGCAGCCCACC
TACGCGGCCAACACCATCACCAACTTCATAACGACAACATATGGAATGGCCGTGACCCTTGGTATACC
TGAGGCCGACTGGCCCCGACTGGTAAGGGACCTCACAGATGAGAGGGGAAACAGGCGTCTCCTCGTCT
CGGGGGACGACAAGGTTCTTTTCTTAAGAGGAGATGAGGCTAGGGCTTATGCCTCATCGGCCTACCGG
ATAAGTAACGAC

>MT319_ON158827
GAGAYCGAGTAAAGAAGTCAATGGACGCCACCGGCGCCGCTATAGCGGAAGACGTGGCGGGCTGGGAC
ACAAAGATCTCCAAAGGACTGCTCACCCTGGAGTCCCATATGTTCACCAAGCTGGCGGAGGATGAAGA
AATGGCGCGGGAGATCCACCACCTCTACCGACTCTACGCAGATCCACACATGGTGGTCCAGAGGGAGA
TTGAAGGTGAGGTTCATGACGTACTCCTTAGGGGGAGGGGGCAGGTCTCTTCCGGGAGGCAGCCCACC
TACGCGGCCAACACCATCACCAACTTCATAACGACAACATATGGAATGGCCGTGACCCTTGGTATACC
TGAGGCCGACTGGCCCCGACTGATAAGGGACCTCACAGATGAGAGGGGAAACAGGCGTCTCCTCGTCT
CGGGGGACGACAAGGTCCTTTTCTTAAGAGGAGATGAGGCTAGGGCTTATGCCTCATCGGCCTACCGG
ATAAGTAACGAC

>MT336_ON158828

GAGATCGAGTAAAGAAGTCAATGGACGCCACCGGCGCCGCTATAGCGGAAGACGTGGCGGGCTGGGAC
ACCAAGATCTCCAAAGGACTGCTCACCCTGGAGTCCCATATGTTCACCAAGCTGGCGGAGGATGAAGA
AATGGCGCGGGAGATCCACCACCTCTACCGACTCTACGCAGATCCACACATGGTGGTCCAGAGGGAGA
TTGAAGGTGAGGTCCATGACGTACTCCTTAGGGGGAGGGGGCAGGTCTCCTCCGGGAGGCAGCCCACC
TACGCGGCCAACACCATCACCAACTTCATAACGACAACATATGGAATGGCCGTGACCCTTGGTATACC
TGAGGCCGACTGGCCCCGACTGATAAGGGACCTCACAGATGAGAGGGGAAACAGGCGTCTCCTCGTCT
CGGGGGACGACAAGGTCCTTTTCTTAAGGGGAGATGAGGCTAGGGTTTACGCCTCATCGGCCTACCGG
ATAAGTAACGAC

>MT314_ON158829
GAGATCGAGTAAAGAAGTCAATGGACGCCACCGGCGCCGCTATAGCGGAAGACGTGGCGGGCTGGGAC
ACCAAGATCTCCAAAGGACTGCTCACCCCGGAGTCCCATATGTTCACCAAGCTGGCGGAGGATGAAGA
AATGGCGCGGGAGATCCACCACCTCTACCGACTCTACGCAGATCCACACATGGTGGTCCAGAGGGAGA
TTGAAGGTGAGGTCCATGACGTACTCCTTAGGGGGAGGGGGCAGGTCTCCTCCGGGAGGCAGCCCACC
TACGCGGCCAACACCATCACCAACTTCATAACGACAACATATGGAATGGCCGTGACCCTTGGTATACC
TGAGGCCGACTGGCCCCGACTGATAAGGGACCTCACAGATGAGAGGGGAAACAGGCGTCTCCTCGTCT
CGGGGGACGACAAGGTCCTTTTCTTAAGGGGAGATGAGGCTAGGGTTTACGCCTCATCGGCCTACCGG
ATAAGTAACGAC

>MT324_ON158830
GAGATCGAGTAAAGAAGTCAATGGACGCCACCGGCGCCGCTATAGCGGAAGACGTGGCGGGCTGGGAC
ACCAAGATCTCCAAAGGACTGCTCACCCTGGAGTCCCATATGTTCACCAAGCTAGCGGAGGATGAAGA
AATGGCGCGGGAGATCCACCACCTCTACCGACTCTACGCAGATCCACACATGGTGGTCCAGAGGGAGA
TTGAAGGTGAGGTCCATGACGTACTCCTTAGGGGGAGGGGGCAGGTCTCCTCCGGGAGGCAGCCCACC
TACGCGGCCAACACCATCACCAACTTCATAACGACAACATATGGAATGGCCGTGACCCTTGGTATACC
TGAGGCCGACTGGCCCCGACTGATAAGGGACCTCACAGATGAGAGGGGAAACAGGCGTCTCCTCGTCT
CGGGGGACGACAAGGTCCTTTTCTTAAGGGGAGATGAGGCTAGGGTTTACGCCTCGTCGGCCTACCGG
ATAAGTAACGAC

>MT292_ON158831
GAGATCGAGTAAAGAAGTCAATGGACGCCACCGGCGCCGCTATAGCGGAAGACGTGGCGGGCTGGGAC
ACCAAGATCTCCAAAGGACTGCTCACCCTGGAGTCCCATATGTTCACCAAGCTAGCGGAGGATGAAGA
AATGGCGCGGGAGATCCACCACCTCTACCGACTCTACGCAGATCCACACATGGTGGTCCAGAGGGAGA
TTGAAGGTGAGGTCCATGACGTACTCCTTAGGGGGAGGGGGCAGGTCTCCTCCGGGAGGCAGCCCACC
TACGCGGCCAACACCATCACCAACTTCATAACGACAACATATGGAATGGCCGTGACCCTTGGTATACC
TGAGGCCGACTGGCCCCGACTGATAAGGGACCTCACAGATGAGAGGGGAAACAGGCGTCTCCTCGTCT
CGGGGGACGACAAGGTCCTTTTCTTAAGGGGAGATGAGGCTAGGGTTTACGCCTCGTCGGCCTACCGG
ATAAGTAACGAC

>MT320_ON158832
GAGATCGAGTAAAGAAGTCAATGGACGCCACCGGCGCCGCTATAGCGGAAGACGTGGCGGGCTGGGAC
ACCAAGATCTCCAAAGGACTGCTCACCCTGGAGTCCCATATGTTCACCAAGCTAGCGGAGGATGAAGA
AATGGCGCGGGAGATCCACCACCTCTACCGACTCTACGCAGATCCACACATGGTGGTCCAGAGGGAGA
TTGAAGGTGAGGTCCATGACGTACTCCTTAGGGGGAGGGGGCAGGTCTCCTCCGGGAGGCAGCCCACC
TACGCGGCCAACACCATCACCAACTTCATAACGACAACATATGGAATGGCCGTGACCCTTGGTATACC
TGAGGCCGACTGGCCCCGACTGATAAGGGACCTCACAGATGAGAGGGGAAACAGGCGTCTCCTCGTCT
CGGGGGACGACAAGGTCCTTTTCTTAAGGGGAGATGAGGCTAGGGTTTACGCCTCGTCGGCCTACCGG
ATAAGTAACGAC

>MT317_ON158833
GAGATCGAGTAAAGAAGTCAATGGACGCCACCGGCGCCGCTATAGCGGAAGACGTGGCGGGCTGGGAC
ACCAAGATCTCCAAAGGACTGCTCACCCTGGAGTCCCATATGTTCACCAAGCTAGCGGAGGATGAAGA
AATGGCGCGGGAGATCCACCACCTCTACCGACTCTACGCAGATCCACACATGGTGGTCCAGAGGGAGA
TTGAAGGTGAGGTCCATGACGTACTCCTTAGGGGGAGGGGGCAGGTCTCCTCCGGGAGGCAGCCCACC
TACGCGGCCAACACCATCACCAACTTCATAACGACAACATATGGAATGGCCGTGACCCTTGGTATACC
TGAGGCCGACTGGCCCCGACTGATAAGGGACCTCACAGATGAGAGGGGAAACAGGCGTCTCCTCGTCT
CGGGGGACGACAAGGTCCTTTTCTTAAGGGGAGATGAGGCTAGGGTTTACGCCTCGTCGGCCTACCGG
ATAAGTAACGAC

>MT302_ON158834
GAGATCGAGTAAAGAAGTCAATGGACGCCACCGGCGCCGCTATAGCGGAAGACGTGGCGGGCTGGGAC
ACCAAGATCTCCAAAGGACTGCTCACCCTGGAGTCCCATATGTTCACCAAGCTAGCGGAGGATGAAGA
AATGGCGCGGGAGATCCACCACCTCTACCGACTCTACGCAGATCCACACATGGTGGTCCAGAGGGAGA
TTGAAGGTGAGGTCCATGACGTACTCCTTAGGGGGAGGGGGCAGGTCTCCTCCGGGAGGCAGCCCACC
TACGCGGCCAACACCATCACCAACTTCATAACGACAACATATGGAATGGCCGTGACCCTTGGTATACC
TGAGGCCGACTGGCCCCGACTGATAAGGGACCTCACAGATGAGAGGGGAAACAGGCGTCTCCTCGTCT
CGGGGGACGACAAGGTCCTTTTCTTAAGGGGAGATGAGGCTAGGGTTTACGCCTCGTCGGCCTACCGG
ATAAGTAACGAC

>MT297_ON158835 -
GAGATCGAGTAAAGAAGTCAATGGACGCCACCGGCGCCGCTATAGCGGAAGACGTGGCGGGCTGGGAC
ACCAAGATCTCCAAAGGACTGCTCACCCTGGAGTCCCATATGTTCACCAAGCTAGCGGAGGATGAAGA
AATGGCGCGGGAGATCCACCACCTCTACCGACTCTACGCAGATCCACACATGGTGGTCCAGAGGGAGA
TTGAAGGTGAGGTCCATGACGTACTCCTTAGGGGGAGGGGGCAGGTCTCCTCCGGGAGGCAGCCCACC
TACGCGGCCAACACCATCACCAACTTCATAACGACAACATATGGAATGGCCGTGACCCTTGGTATACC
TGAGGCCGACTGGCCCCGACTGATAAGGGACCTCACAGATGAGAGGGGAAACAGGCGTCTCCTCGTCT
CGGGGGACGACAAGGTCCTTTTCTTAAGGGGAGATGAGGCTAGGGTTTACGCCTCGTCGGCCTACCGG
ATAAGTAACGAC

>MT306_ON158836
GAGATCGAGTAAAGAAGTCAATGGACGCCACCGGCGCCGCTATAGCGGAAGACGTGGCGGGCTGGGAC
ACCAAGATCTCCAAAGGACTGCTCACCCTGGAGTCCCATATGTTCACCAAGCTAGCGGAGGATGAAGA
AATGGCGCGGGAGATCCACCACCTCTACCGACTCTACGCAGATCCACACATGGTGGTCCAGAGGGAGA
TTGAAGGTGAGGTCCATGACGTACTCCTTAGGGGGAGGGGGCAGGTCTCCTCCGGGAGGCAGCCCACC
TACGCGGCCAACACCATCACCAACTTCATAACGACAACATATGGAATGGCCGTGACCCTTGGTATACC
TGAGGCCGACTGGCCCCGACTGATAAGGGACCTCACAGATGAGAGGGGAAACAGGCGTCTCCTCGTCT
CGGGGGACGACAAGGTCCTTTTCTTAAGGGGAGATGAGGCTAGGGTTTACGCCTCGTCGGCCTACCGG
ATAAGTAACGAC

>MT294_ON158837
GAGATCGAGTAAAGAAGTCAATGGACGCCACCGGCGCCGCTATAGCGGAAGACGTGGCGGGCTGGGAC
ACCAAGATCTCCAAAGGACTGCTCACCCTGGAGTCCCATATGTTCACCAAGCTRGCGGAGGATGAAGA
AATGGCGCGGGAGATCCACCACCTCTACCGACTCTACGCAGATCCACACATGGTGGTCCAGAGGGAGA
TTGAAGGTGAGGTCCATGACGTACTCCTTAGGGGGAGGGGGCAGGTCTCCTCCGGGAGGCAGCCCACC
TACGCGGCCAACACCATCACCAACTTCATAACGACAACATATGGAATGGCCGTGACCCTTGGTATACC
TGAGGCCGACTGGCCCCGACTGATAAGGGACCTCACAGATGAGAGGGGAAACAGGCGTCTCCTCGTCT

CGGGGGACGACAAGGTCCTTTTCTTAAGGGGAGATGAGGCTAGGGTTTACGCCTCGTCGGCCTACCGG
ATAAGTAACGAC

>MT308_ON158838
GAGATCGAGTAAAGAAGTCAATGGACGCCACCGGCGCCGCTATAGCGGAAGACGTGGCGGGCTGGGAC
ACCAAGATCTCCAAAGGACTGCTCACCCCGGAGTCCCATATGTTCACCAAGCTAGCGGAGGATGAAGA
AATGGCGCGGGAGATCCACCACCTCTACCGACTCTACGCAGATCCACACATGGTGGTCCAGAGGGAGA
TTGAAGGTGAGGTCCATGACGTACTCCTTAGGGGGAGGGGGCAGGTCTCCTCCGGGAGGCAGCCCACC
TACGCGGCCAACACCATCACCAACTTCATAACGACAACATATGGAATGGCCGTGACCCTTGGTATACC
TGAGGCCGACTGGCCCCGACTGATAAGGGACCTCACAGATGAGAGGGGAAACAGGCGTCTCCTCGTCT
CGGGGGACGACAAGGTCCTTTTCTTAAGGGGAGATGAGGCTAGGGTTTACGCCTCGTCGGCCTACCGG
ATAAGTAACGAC

>MT304_ON158839
GAGATCGAGTAAAGAAGTCAATGGACGCCACGGGCGCCGCTATAGCGGAAGACGTGGCGGGCTGGGAC
ACCAAGATCTCCAAAGGACTGCTCACCCTGGAGTCCCATATGTTCACCAAGCTAGCGGAGGATGAAGA
AATGGCGCGGGAGATCCACCACCTCTACCGACTCTACGCAGATCCACACATGGTGGTCCAGAGGGAGA
TTGAAGGTGAGGTCCATGACGTACTCCTTAGGGGGAGGGGGCAGGTCTCCTCCGGGAGGCAGCCCACC
TACGCGGCCAACACCATCACCAACTTCATAACGACAACATATGGAATGGCCGTGACCCTTGGTATACC
TGAGGCCGACTGGCCCCGACTGATAAGGGACCTCACAGATGAGAGGGGAAACAGGCGTCTCCTCGTCT
CGGGGGACGACAAGGTCCTTTTCTTAAGGGGAGATGAGGCTAGGGTTTACGCCTCGTCGGCCTACCGG
ATAAGTAACGAC

>MT303_ON158840
GAGATCGAGTAAAGAAGTCAATGGACGCCACGGGCGCCGCTATAGCGGAAGACGTGGCGGGCTGGGAC
ACCAAGATCTCCAAAGGACTGCTCACCCTGGAGTCCCATATGTTCACCAAGCTAGCGGAGGATGAAGA
AATGGCGCGGGAGATCCACCACCTCTACCGACTCTACGCAGATCCACACATGGTGGTCCAGAGGGAGA
TTGAAGGTGAGGTCCATGACGTACTCCTTAGGGGGAGGGGGCAGGTCTCCTCCGGGAGGCAGCCCACC
TACGCGGCCAACACCATCACCAACTTCATAACGACAACATATGGAATGGCCGTGACCCTTGGTATACC
TGAGGCCGACTGGCCCCGACTGATAAGGGACCTCACAGATGAGAGGGGAAACAGGCGTCTCCTCGTCT
CGGGGGACGACAAGGTCCTTTTCTTAAGGGGAGATGAGGCTAGGGTTTACGCCTCGTCGGCCTACCGG
ATAAGTAACGAC

>MT298_ON158841
GAGATCGAGTAAAGAAGTCAATGGACGCCACGGGCGCCGCTATAGCGGAAGACGTGGCGGGCTGGGAC
ACCAAGATCTCCAAAGGACTGCTCACCCTGGAGTCCCATATGTTCACCAAGCTAGCGGAGGATGAAGA
AATGGCGCGGGAGATCCACCACCTCTACCGACTCTACGCAGATCCACACATGGTGGTCCAGAGGGAGA
TTGAAGGTGAGGTCCATGACGTACTCCTTAGGGGGAGGGGGCAGGTCTCCTCCGGGAGGCAGCCCACC
TACGCGGCCAACACCATCACCAACTTCATAACGACAACATATGGAATGGCCGTGACCCTTGGTATACC
TGAGGCCGACTGGCCCCGACTGATAAGGGACCTCACAGATGAGAGGGGAAACAGGCGTCTCCTCGTCT
CGGGGGACGACAAGGTCCTTTTCTTAAGGGGAGATGAGGCTAGGGTTTACGCCTCGTCGGCCTACCGG
ATAAGTAACGAC

>MT300_ON158842
GAGATCGAGTAAAGAAGTCAATGGACGCCACGGGCGCCGCTATAGCGGAAGACGTGGCGGGCTGGGAC
ACCAAGATCTCCAAAGGACTGCTCACCCCGGAGTCCCATATGTTCACCAAGCTAGCGGAGGATGAAGA
AATGGCGCGGGAGATCCACCACCTCTACCGACTCTACGCAGATCCACACATGGTGGTCCAGAGGGAGA
TTGAAGGTGAGGTCCATGACGTACTCCTTAGGGGGAGGGGGCAGGTCTCCTCCGGGAGGCAGCCCACC

TACGCGGCCAACACCATCACCAACTTCATAACGACAACATATGGAATGGCCGTGACCCTTGGTATACC
TGAGGCCGACTGGCCCCGACTGATAAGGGACCTCACAGATGAGAGGGGAAACAGGCGTCTCCTCGTCT
CGGGGGACGACAAGGTCCTTTTCTTAAGGGGAGATGAGGCTAGGGTTTACGCCTCGTCGGCCTACCGG
ATAAGTAACGAC

>MT305_ON158843
GAGATCGAGTAAAGAAGTCAATGGACGCCACAGGCGCCGCTATAGCGGAAGACGTGGCGGGCTGGGAC
ACCAAGATCTCCAAAGGACTGCTCACCCCGGAGTCCCATATGTTCACCAAGCTAGCGGAGGATGAAGA
AATGGCGCGGGAGATCCACCACCTCTACCGACTCTACGCAGATCCACACATGGTGGTCCAGAGGGAGA
TTGAAGGTGAGGTCCATGACGTACTCCTTAGGGGGAGGGGGCAGGTCTCCTCCGGGAGGCAGCCCACC
TACGCGGCCAACACCATCACCAACTTCATAACGACAACATATGGAATGGCCGTGACCCTTGGTATACC
TGAGGCCGACTGGCCCCGACTGATAAGGGACCTCACAGATGAGAGGGGAAACAGGCGTCTCCTCGTCT
CGGGGGACGACAAGGTCCTTTTCTTAAGGGGAGATGAGGCTAGGGTTTACGCCTCGTCGGCCTACCGG
ATAAGTAACGAC

>MT290_ON158844
GAGATCGAGTAAAGAAGTCAATGGACGCCACCGGCGCCGCTATAGCGGAAGACGTGGCGGGCTGGGAC
ACCAAGATCTCCAAAGGACTGCTCACCCCGGAGTCCCATATGTTCACCCAGCTAGCGGAGGATGAAGA
AATGGCGCGGGAGATCCACCACCTCTACCGACTCTACGCAGATCCACACATGGTGGTCCAGAGGGAGA
TTGAAGGTGAGGTCCATGACGTACTCCTTAGGGGGAGGGGGCAGGTCTCCTCCGGGAGGCAGCCCACC
TACGCGGCCAACACCATCACCAACTTCATAACGACAACATATGGAATGGCCGTGACCCTTGGTATACC
TGAGGCCGACTGGCCCCGACTGATAAGGGACCTCACAGATGAGAGGGGAAACAGGCGTCTCCTCGTCT
CGGGGGACGACAAGGTCCTTTTCTTAAGGGGAGATGAGGCTAGGGTTTACGCCTCGTCGGCCTACCGG
ATAAGTAACGAC

>MT295_ON158845
TAAAGAAGTCAATGGACGCCACCGGCGCCGCTATAGCGGAAGACGTGGCGGGCTGGGACACCAAGATC
TCCAAAGGACTGCTCACCCTGGAGTCCCATATGTTCACCAAGCTGGCGGAGGATGAAGAAATGGCGCG
GGAGATCCACCACCTCTACCGACTCTACGCAGATCCACACATGGTGGTCCAGAGGGAGATTGAAGGTG
AGGTCCATGACGTACTCCTTAGGGGGAGGGGGCAGGTCTCCTCCGGGAGGCAGCCCACCTACGCGGCC
AACACCATCACCAACTTCATAACGACAACATATGGAATGGCCGTGACCCTTGGTATACCCGAGGCCGA
CTGGCCCCGACTGATAAGGGACCTCACAGATGAGAGGGGAAACAGGCGCCTCCTCGTCTCGGGGGACG
ACAAGGTCCTTTTCTTAAGGGGAGATGAGGCTAGGGTTTACGCCTCGTCGGCCTACCGGATAAGTAAC
GAC

>MT293_ON158846
GAGACCGGGTAAAGAAGTCAATGGACGCCACCGGCGCCGCTATAGCGGAAGACGTGGCGGGCTGGGAC
ACTAAGATCTCCAAAGGACTACTCACCCTGGAGTCCCATATGTTCACCAAGCTGGCGGAGGATGAAGA
AATGGCGCGGGAGATCCACCACCTCTACCGACTCTACGCAGATCCACACATGGTGGTCCAGAGGGAGA
TTGAAGGTGAGGTCCATGACGTACTCCTTAGGGGGAGGGGGCAGGTCTCCTCCGGGAGGCAGCCCACC
TACGCGGCCAACACCATCACCAACTTCATAACGACAACATATGGAATGGCCGTGACCCTTGGTATACC
CGAGGCCGACTGGCCCCGACTGATAAGGGACCTCACAGATGAGAGGGGAAACAGGCGCCTCCTCGTCT
CGGGGGACGACAAGGTCCTTTTCCTAAGAGGAGATGAGGCTAGGGTTTACGCCTCATCGGCCTACCGG
ATAAGTAACGAC

>MT291_ON158847
GAGACCGGGTAAAGAAGTCAATGGACGCCACCGGCGCCGCTATAGCGGAAGACGTGGCGGGCTGGGAC
ACTAAGATCTCCAAAGGACTACTCACCCTGGAGTCCCATATGTTCACCAAGCTGGCGGAGGATGAAGA

AATGGCGCGGGAGATCCACCACCTCTACCGACTCTACGCAGATCCACACATGGTGGTCCAGAGGGAGA
TTGAAGGTGAGGTCCATGACGTACTCCTTAGGGGGAGGGGGCAGGTCTCCTCCGGGAGGCAGCCCACC
TACGCGGCCAACACCATCACCAACTTCATAACGACAACATATGGAATGGCCGTGACCCTTGGTATACC
CGAGGCCGACTGGCCCCGACTGATAAGGGACCTCACAGATGAGAGGGGAAACAGGCGCCTCCTCGTCT
CGGGGGACGACAAGGTCCTTTTCCTAAGAGGAGATGAGGCTAGGGTTTACGCCTCATCGGCCTACCGG
ATAAGTAACGAC

>MT327_ON158848
GAGACCGGGTAAAGAAGTCAATGGACGCCACCGGCGCCGCTATAGCGGAAGACGTGGCGGGCTGGGAC
ACTAAGATCTCCAAAGGACTACTCACCCTGGAGTCCCATATGTTCACCAAGCTGGCGGAGGATGAAGA
AATGGCGCGGGAGATCCACCACCTCTACCGACTCTACGCAGATCCACACATGGTGGTCCAGAGGGAGA
TTGAAGGTGAGGTCCATGACGTACTCCTTAGGGGGAGGGGGCAGGTCTCCTCCGGGAGGCAGCCCACC
TACGCGGCCAACACCATCACCAACTTCATAACGACAACATATGGAATGGCCGTGACCCTTGGTATACC
CGAGGCCGACTGGCCCCGACTGATAAGGGACCTCACAGATGAGAGGGGAAACAGGCGCCTCCTCGTCT
CGGGGGACGACAAGGTCCTTTTCCTAAGAGGAGATGAGGCTAGGGTTTACGCCTCATCGGCCTACCGG
ATAAGTAACGAC

>MT307_ON158849
GAGATCGAGTAAAGAAGTCAATGGACGCCACCGGCGCCGCTATAGCGGAAGACGTGGCGGGCTGGGAC
ACCAAGATCTCCAAAGGACTGCTCACCCTGGAGTCCCATATGTTCACCAAGCTAGCGGAGGATGAAGA
AATGGCGCGGGAGATCCACCACCTCTACCGACTCTACGCAGATCCACACATGGTGGTCCAGAGGGAGA
TTGAAGGTGAGGTCCATGACGTACTCCTTAGGGGGAGGGGGCAGGTCTCCTCCGGGAGGCAGCCCACC
TACGCGGCCAACACCATCACCAACTTCATAACGACAACATATGGAATGGCCGTGACCCTTGGTATACC
TGAGGCCGACTGGCCCCGACTGATAAGGGACCTCACAGATGAGAGGGGAAACAGGCGTCTCCTCGTCT
CGGGGGACGACAAGGTCCTTTTCTTAAGGGGAGATGAGGCTAGGGTTTACGCCTCGTCGGCCTACCGG
ATAAGTAACGAC

>MT161_ON158850
GAGACCGAGTAAAGAAGTCAATGGACGCTACCGGCGCCGCTATAGCGGAAGACGTGGCGGGCTGGGAC
ACAAAGATCTCCAAAGGACTGCTCACCCTGGAGTCCCATATGTTCACCAAGCTGGCGGAGGATGAAGA
AATGGCACGGGAGATCCACCACCTCTACCGACTCTACGCAGATCCACACATGGTGGTCCAGAGGGAGA
TCGAAGGTGAAGTCCATGACGTACTCCTTAGGGGGAGGGGACAGGTCTCCTCCGGGAGGCAGCCCACC
TACGCGGCCAACACCATCACCAACTTCATAACGACAACATATGGAATGGCCGTGACCCTTGGTATACC
TGAGGCCGACTGGCCCCGACTGATAAGGGACCTCACAGATGAGAGGGGAAACAGGCGTCTCCTCGTCT
CGGGGGACGACAAGGTCCTTTTCTTAAGGGGAGATGAGGCTAGGGTTTACGCCTCATCGGCCTACCGG
ATAAGTAACGAC

>MT156_ON158851
GAGACCGAGTAAAGAAGTCAATGGACGCTACCGGCGCCGCTATAGCGGAAGACGTGGCGGGCTGGGAC
ACAAAGATCTCCAAAGGACTGCTCACCCTGGAGTCCCATATGTTCACCAAGCTGGCGGAGGATGAAGA
AATGGCACGGGAGATCCACCACCTCTACCGACTCTACGCAGATCCACACATGGTGGTCCAGAGGGAGA
TCGAAGGTGAAGTCCATGACGTACTCCTTAGGGGGAGGGGACAGGTCTCCTCCGGGAGGCAGCCCACC
TACGCGGCCAACACCATCACCAACTTCATAACGACAACATATGGAATGGCCGTGACCCTTGGTATACC
TGAGGCCGACTGGCCCCGACTGATAAGGGACCTCACAGATGAGAGGGGAAACAGGCGTCTCCTCGTCT
CGGGGGACGACAAGGTCCTTTTCTTAAGGGGAGATGAGGCTAGGGTTTACGCCTCATCGGCCTACCGG
ATAAGTAACGAC

>MT150_ON158852

GAGACCGAGTAAAGAAGTCAATGGACGCTACCGGCGCCGCTATAGCGGAAGACGTGGCGGGCTGGGAC
ACAAAGATCTCCAAAGGACTGCTCACCCTGGAGTCCCATATGTTCACCAAGCTGGCGGAGGATGAAGA
AATGGCACGGGAGATCCACCACCTCTACCGACTCTACGCAGATCCACACATGGTGGTCCAGAGGGAGA
TCGAAGGTGAAGTCCATGACGTACTCCTTAGGGGGAGGGGACAGGTCTCCTCCGGGAGGCAGCCCACC
TACGCGGCCAACACCATCACCAACTTCATAACGACAACATATGGAATGGCCGTGACCCTTGGTATACC
TGAGGCCGACTGGCCCCGACTGATAAGGGACCTCACAGATGAGAGGGGAAACAGGCGTCTCCTCGTCT
CGGGGGACGACAAGGTCCTTTTCTTAAGGGGAGATGAGGCTAGGGTTTACGCCTCATCGGCCTACCGG
ATAAGTAACGAC

>MT152_ON158853
GAGACCGAGTAAAGAAGTCAATGGACGCTACCGGCGCCGCTATAGCGGAAGACGTGGCGGGCTGGGAC
ACAAAGATCTCCAAAGGACTGCTCACCCTGGAGTCCCATATGTTCACCAAGCTGGCGGAGGATGAAGA
AATGGCACGGGAGATCCACCACCTCTACCGACTCTACGCAGATCCACACATGGTGGTCCAGAGGGAGA
TCGAAGGTGAAGTCCATGACGTACTCCTTAGGGGGAGGGGACAGGTCTCCTCCGGGAGGCAGCCCACC
TACGCGGCCAACACCATCACCAACTTCATAACGACAACATATGGAATGGCCGTGACCCTTGGTATACC
TGAGGCCGACTGGCCCCGACTGATAAGGGACCTCACAGATGAGAGGGGAAACAGGCGTCTCCTCGTCT
CGGGGGACGACAAGGTCCTTTTCTTAAGGGGAGATGAGGCTAGGGTTTACGCCTCATCGGCCTACCGG
ATAAGTAACGAC

>MT148_ON158854
GAGACCGAGTAAAGAAGTCAATGGACGCTACCGGCGCCGCTATAGCGGAAGACGTGGCGGGCTGGGAC
ACAAAGATCTCCAAAGGACTGCTCACCCTGGAGTCCCATATGTTCACCAAGCTGGCGGAGGATGAAGA
AATGGCACGGGAGATCCACCACCTCTACCGACTCTACGCAGATCCACACATGGTGGTCCAGAGGGAGA
TCGAAGGTGAAGTCCATGACGTACTCCTTAGGGGGAGGGGACAGGTCTCCTCCGGGAGGCAGCCCACC
TACGCGGCCAACACCATCACCAACTTCATAACGACAACATATGGAATGGCCGTGACCCTTGGTATACC
TGAGGCCGACTGGCCCCGACTGATAAGGGACCTCACAGATGAGAGGGGAAACAGGCGTCTCCTCGTCT
CGGGGGACGACAAGGTCCTTTTCTTAAGGGGAGATGAGGCTAGGGTTTACGCCTCATCGGCCTACCGG
ATAAGTAACGAC

>MT157_ON158855
GAGACCGAGTAAAGAAGTCAATGGACGCTACCGGCGCCGCTATAGCGGAAGACGTGGCGGGCTGGGAC
ACAAAGATCTCCAAAGGACTGCTCACCCTGGAGTCCCATATGTTCACCAAGCTGGCGGAGGATGAAGA
AATGGCACGGGAGATCCACCACCTCTACCGACTCTACGCAGATCCACACATGGTGGTCCAGAGGGAGA
TCGAAGGTGAAGTCCATGACGTACTCCTTAGGGGGAGGGGACAGGTCTCCTCCGGGAGGCAGCCCACC
TACGCGGCCAACACCATCACCAACTTCATAACGACAACATATGGAATGGCCGTGACCCTTGGTATACC
TGAGGCCGACTGGCCCCGACTGATAAGGGACCTCACAGATGAGAGGGGAAACAGGCGTCTCCTCGTCT
CGGGGGACGACAAGGTCCTTTTCTTAAGGGGAGATGAGGCTAGGGTTTACGCCTCATCGGCCTACCGG
ATAAGTAACGAC

>MT153_ON158856
GAGACCGAGTAAAGAAGTCAATGGACGCTACCGGCGCCGCTATAGCGGAAGACGTGGCGGGCTGGGAC
ACAAAGATCTCCAAAGGACTGCTCACCCTGGAGTCCCATATGTTCACCAAGCTGGCGGAGGATGAAGA
AATGGCACGGGAGATCCACCACCTCTACCGACTCTACGCAGATCCACACATGGTGGTCCAGAGGGAGA
TCGAAGGTGAAGTCCATGACGTACTCCTTAGGGGGAGGGGACAGGTCTCCTCCGGGAGGCAGCCCACC
TACGCGGCCAACACCATCACCAACTTCATAACGACAACATATGGAATGGCCGTGACCCTTGGTATACC
TGAGGCCGACTGGCCCCGACTGATAAGGGACCTCACAGATGAGAGGGGAAACAGGCGTCTCCTCGTCT
CGGGGGACGACAAGGTCCTTTTCTTAAGGGGAGATGAGGCTAGGGTTTACGCCTCATCGGCCTACCGG
ATAAGTAACGAC

>MT147_ON158857
GAGACCGAGTAAAGAAGTCAATGGACGCTACCGGCGCCGCTATAGCGGAAGACGTGGCGGGCTGGGAC
ACAAAGATCTCCAAAGGACTGCTCACCCTGGAGTCCCATATGTTCACCAAGCTGGCGGAGGATGAAGA
AATGGCACGGGAGATCCACCACCTCTACCGACTCTACGCAGATCCACACATGGTGGTCCAGAGGGAGA
TCGAAGGTGAAGTCCATGACGTACTCCTTAGGGGGAGGGGACAGGTCTCCTCCGGGAGGCAGCCCACC
TACGCGGCCAACACCATCACCAACTTCATAACGACAACATATGGAATGGCCGTGACCCTTGGTATACC
TGAGGCCGACTGGCCCCGACTGATAAGGGACCTCACAGATGAGAGGGGAAACAGGCGTCTCCTCGTCT
CGGGGGACGACAAGGTCCTTTTCTTAAGGGGAGATGAGGCTAGGGTTTACGCCTCATCGGCCTACCGG
ATAAGTAACGAC

>MT146_ON158858
GAGACCGAGTAAAGAAGTCAATGGACGCTACCGGCGCCGCTATAGCGGAAGACGTGGCGGGCTGGGAC
ACAAAGATCTCCAAAGGACTGCTCACCCTGGAGTCCCATATGTTCACCAAGCTGGCGGAGGATGAAGA
AATGGCACGGGAGATCCACCACCTCTACCGACTCTACGCAGATCCACACATGGTGGTCCAGAGGGAGA
TCGAAGGTGAAGTCCATGACGTACTCCTTAGGGGGAGGGGACAGGTCTCCTCCGGGAGGCAGCCCACC
TACGCGGCCAACACCATCACCAACTTCATAACGACAACATATGGAATGGCCGTGACCCTTGGTATACC
TGAGGCCGACTGGCCCCGACTGATAAGGGACCTCACAGATGAGAGGGGAAACAGGCGTCTCCTCGTCT
CGGGGGACGACAAGGTCCTTTTCTTAAGGGGAGATGAGGCTAGGGTTTACGCCTCATCGGCCTACCGG
ATAAGTAACGAC

>MT155_ON158859
GAGACCGAGTAAAGAAGTCAATGGACGCTACCGGCGCCGCTATAGCGGAAGACGTGGCGGGCTGGGAC
ACAAAGATCTCCAAAGGACTGCTCACCCTGGAGTCCCATATGTTCACCAAGCTGGCGGAGGATGAAGA
AATGGCACGGGAGATCCACCACCTCTACCGACTCTACGCAGATCCACACATGGTGGTCCAGAGGGAGA
TCGAAGGTGAAGTCCATGACGTACTCCTTAGGGGGAGGGGACAGGTCTCCTCCGGGAGGCAGCCCACC
TACGCGGCCAACACCATCACCAACTTCATAACGACAACATATGGAATGGCCGTGACCCTTGGTATACC
TGAGGCCGACTGGCCCCGACTGATAAGGGACCTCACAGATGAGAGGGGAAACAGGCGTCTCCTCGTCT
CGGGGGACGACAAGGTCCTTTTCTTAAGGGGAGATGAGGCTAGGGTTTACGCCTCATCGGCCTACCGG
ATAAGTAACGAC

>MT154_ON158860
GAGACCGAGTAAAGAAGTCAATGGACGCTACCGGCGCCGCTATAGCGGAAGACGTGGCGGGCTGGGAC
ACAAAGATCTCCAAAGGACTGCTCACCCTGGAGTCCCATATGTTCACCAAGCTGGCGGAGGATGAAGA
AATGGCACGGGAGATCCACCACCTCTACCGACTCTACGCAGATCCACACATGGTGGTCCAGAGGGAGA
TCGAAGGTGAAGTCCATGACGTACTCCTTAGGGGGAGGGGACAGGTCTCCTCCGGGAGGCAGCCCACC
TACGCGGCCAACACCATCACCAACTTCATAACGACAACATATGGAATGGCCGTGACCCTTGGTATACC
TGAGGCCGACTGGCCCCGACTGATAAGGGACCTCACAGATGAGAGGGGAAACAGGCGTCTCCTCGTCT
CGGGGGACGACAAGGTCCTTTTCTTAAGGGGAGATGAGGCTAGGGTTTACGCCTCATCGGCCTACCGG
ATAAGTAACGAC

>MT159_ON158861
GAGACCGAGTAAAGAAGTCAATGGACGCTACCGGCGCCGCTATAGCGGAAGACGTGGCGGGCTGGGAC
ACAAAGATCTCCAAAGGACTGCTCACCCTGGAGTCCCATATGTTCACCAAGCTGGCGGAGGATGAAGA
AATGGCACGGGAGATCCACCACCTCTACCGACTCTACGCAGATCCACACATGGTGGTCCAGAGGGAGA
TCGAAGGTGAAGTCCATGACGTACTCCTTAGGGGGAGGGGACAGGTCTCCTCCGGGAGGCAGCCCACC
TACGCGGCCAACACCATCACCAACTTCATAACGACAACATATGGAATGGCCGTGACCCTTGGTATACC
TGAGGCCGACTGGCCCCGACTGATAAGGGACCTCACAGATGAGAGGGGAAACAGGCGTCTCCTCGTCT

CGGGGGACGACAAGGTCCTTTTCTTAAGGGGAGATGAGGCTAGGGTTTACGCCTCATCGGCCTACCGG
ATAAGTAACGAC

>MT151_ON158862
GAGACCGAGTAAAGAAGTCAATGGACGCTACGGGCGCCGCTATAGCGGAAGACGTGGCGGGCTGGGAC
ACAAAGATCTCCAAAGGACTGCTCACCCTGGAGTCCCATATGTTCACCAAGCTGGCGGAGGATGAAGA
AATGGCACGGGAGATCCACCACCTCTACCGACTCTACGCAGATCCACACATGGTGGTCCAGAGGGAGA
TCGAAGGTGAAGTCCATGACGTACTCCTTAGGGGGAGGGGACAGGTCTCCTCCGGGAGGCAGCCCACC
TACGCGGCCAACACCATCACCAACTTCATAACGACAACATATGGAATGGCCGTGACCCTTGGTATACC
TGAGGCCGACTGGCCCCGACTGATAAGGGACCTCACAGATGAGAGGGGAAACAGGCGTCTCCTCGTCT
CGGGGGACGACAAGGTCCTTTTCTTAAGGGGAGATGAGGCTAGGGTTTACGCCTCATCGGCCTACCGG
ATAAGTAACGAC

>MT160_ON158863
GAGACCGAGTAAAGAAGTCAATGGACGCTACGGGCGCCGCTATAGCGGAAGACGTGGCGGGCTGGGAC
ACAAAGATCTCCAAAGGACTGCTCACCCTGGAGTCCCATATGTTCACCAAGCTGGCGGAGGATGAAGA
AATGGCACGGGAGATCCACCACCTCTACCGACTCTACGCAGATCCACACATGGTGGTCCAGAGGGAGA
TCGAAGGTGAAGTCCATGACGTACTCCTTAGGGGGAGGGGACAGGTCTCCTCCGGGAGGCAGCCCACC
TACGCGGCCAACACCATCACCAACTTCATAACGACAACATATGGAATGGCCGTGACCCTTGGTATACC
TGAGGCCGACTGGCCCCGACTGATAAGGGACCTCACAGATGAGAGGGGAAACAGGCGTCTCCTCGTCT
CGGGGGACGACAAGGTCCTTTTCTTAAGGGGAGATGAGGCTAGGGTTTACGCCTCATCGGCCTACCGG
ATAAGTAACGAC

>MT149_ON158864
GAGACCGAGTAAAGAAGTCAATGGACGCTACGGGCGCCGCTATAGCGGAAGACGTGGCGGGCTGGGAC
ACAAAGATCTCCAAAGGACTGCTCACCCTGGAGTCCCATATGTTCACCAAGCTGGCGGAGGATGAAGA
AATGGCACGGGAGATCCACCACCTCTACCGACTCTACGCAGATCCACACATGGTGGTCCAGAGGGAGA
TCGAAGGTGAAGTCCATGACGTACTCCTTAGGGGGAGGGGACAGGTCTCCTCCGGGAGGCAGCCCACC
TACGCGGCCAACACCATCACCAACTTCATAACGACAACATATGGAATGGCCGTGACCCTTGGTATACC
TGAGGCCGACTGGCCCCGACTGATAAGGGACCTCACAGATGAGAGGGGAAACAGGCGTCTCCTCGTCT
CGGGGGACGACAAGGTCCTTTTCTTAAGGGGAGATGAGGCTAGGGTTTACGCCTCATCGGCCTACCGG
ATAAGTAACGAC

>MT158_ON158865
GAGACCGAGTAAAGAAGTCAATGGACGCAACCGGCGCCGCTATAGCGGAAGACGTGGCGGGCTGGGAC
ACAAAGATCTCCAAAGGACTGCTCACCCTGGAGTCCCATATGTTCACCAAGCTGGCGGAGGATGAAGA
AATGGCACGGGAGATCCACCACCTCTACCGACTCTACGCAGATCCACACATGGTGGTCCAGAGGGAGA
TCGAAGGTGAAGTCCATGACGTACTCCTTAGGGGGAGGGGACAGGTCTCCTCCGGGAGGCAGCCCACC
TACGCGGCCAACACCATCACCAACTTCATAACGACAACATATGGAATGGCCGTGACCCTTGGTATACC
TGAGGCCGACTGGCCCCGACTGATAAGGGACCTCACAGATGAGAGGGGAAACAGGCGTCTCCTCGTCT
CGGGGGACGACAAGGTCCTTTTCTTAAGGGGAGATGAGGCTAGGGTTTACGCCTCATCGGCCTACCGG
ATAAGTAACGAC

>MT309_ON158866
GAGACCGAGTAAAGAAGTCAATGGACGCCACGGGCGCCGCTATAGCGGAAGACGTGGCGGGCTGGGAC
ACAAAGATCTCCAAAGGACTGCTCACCCCGGAGTCCCACATGTTCACCAAGTGGGCGGAGGATGAAGA
AATGGCGCGGGAGATCCACCACCTCTACCGACTCTACGCAGATCCACACATGGTGGTCCAGAGGGAGA
TTGAAGGTGAAGTCCATGACGTACTCCTTAGGGGGAGGGGGCAGGTCTCCTCCGGGAGGCAGCCCACC

TACGCGGCCAACACCATCACCAACTTCATAACGACAACATATGGAATGGCCGTGACCCTTGGTATACC
TGAGGCCGACTGGCCCCGACTGATAAGGGACCTCACAGATGAGAGGGGAAACAGGCGTCTCCTCGTCT
CGGGGGACGACAAGGTCCTTTTCTTAAGGGGAGATGAGGCTAGGATCTACGCCTCATCGGCCTACCGG
ATAAGTAACGAC

>MT329_ON158867
GAGATCGAGTAAAGAAGTCAATGGACGCTACCGGCGCCGCTATAGCGGAAGACGTGGCGGGCTGGGAC
ACAAAGATCTCCAAAGGACTGCTCACCCTGGAGTCCCACATGTTCACCAAGCTGGCGGAGGATGAAGA
AATGGCGCGGGAGATCCACCACCTCTACCGACTCTACGCAGATCCACACATGGTGGTCCAGAGGGAGA
TTGAAGGTGAAGTTCATGACGTACTCCTTAGGGGGAGGGGGCAGGTCTCTTCCGGGAGGCAGCCCACC
TACGCGGCCAACACCATCACCAACTTCATAACGACAACATATGGAATGGCCGTGACCCTTGGTATACC
TGAGGCCGACTGGCCCCGACTGGTAAGGGACCTCACAGATGAGAGGGGAAACAGGCGTCTCCTCGTCT
CGGGGGACGACAAGGTTCTTTTCTTAAGGGGAGATGAGGCTAGGGCTTATGCCTCATCGGCCTACCGG
ATAAGTAACGAC

>MT328_ON186499
TTAAGGAATCCGCCTCAGACTCAAACGCAGACGAAACAACAACAAGACAGATAATCGTAAGAAATACA
ATGGACACAGACACCGTGATCGCTGGATTGGCCATGGTGATGCTTATTCTGCTGGCTACGCTCCTGCA
GATGGCATCACACTACAGAGAACATGCCACCTTCATCTACACCCCAGGAGGAAAGCTGGAGGAGTATA
AAGGAGATGGGCTCAAAGTATGGAGGTTAGAGCCCACGTTGGGGGATCCGACCGAACTGGGTAGGCAG
GCAAAGAAAGAGATGAACGCCATGACCTATGAGGAATTCGATAGAATGAAGTTCAGGGGAGTGGTGGC
CGAAGTGTTCGCAGGAGATAAACCATCAAAGGGCTACGATAAACTCAGGATACTACTAGACATAATGG
ACAGACCCAAGTTAGGAACGACGGTTGACCTTTGCGCGGGCCGAGGGGGCTGGAGCCAGCTAGTCAGA
GAGCTAGAGGGACCAGGCGACATCACCGCTGTCTCCCTATGGGAGAGAGGTAGAGAGGAATGGATGGC
TGACCCCGCCATAAGGAGGATCAATGCCAATGTGAAGAATGTCAAGCCCTGGCGTGTAGATACGTTAC
TATTCGACGGGGGGGAGACCTTCAAAAGGGACCAAAACATCATGAAGGAAGAGAGCTACAACGACGCC
CTACTGGACGCTGTGGACGCATGGATGATGCAGGCCACACCTCCCACAAACTTCGTGATAAAAATACA
GGTTCCCTATACACAAAAAGCGATGAGACTCATAGAAAAGTGGCAAGTGAAAACCAAGAAGGGAAGAC
TGGTTAGGCTGGCGGGAGACAGGCTGTCTAATACCGTGATGTACTTCATATCAGATAGGCTGGAAACA
CAGATTCGGGGAAGAATAACCAGCTTCCTCAGGGAGCTCCGGGAGCGAAGAGCGGACAGATCTCTGAC
GTCTGACCCGGCCCTGCAATACGAACGCATAACGCCAGAGTGGACAGCAGAGGCTGTGATCGAGGGGT
GTGCCCCACTCACACCGCTGAACATGACTAGATCTATAGCAGAGATGCATATGGAACACCCACCCTTG
GGGATAACCAGATTCTTCAAAGAGCTCGGCTACAAGATTGCAAAAAAGAAGGGCTCAGAGGGCACGCG
AAGAAACAGGTTCGTGGGCCAGCTCATAGAGCCCCTAAGACGAGTGCTAGAACGACATCACCTCTTCG
GAGCATGGCAACTCACTTCGACAACACCTCGAGCCGTGTTCAACATCTTCAGAAGCAAGGTGGACAGG
GCTCCGGTGGAGCTCCACTCGCACTATCCGGGACTAAGAAAGATGTACGACATTCTCGCTGACCTCTG
GCTGGAGAGGTACGGAAGCATGAAGCGATTAACCGAAGATGAGATGGCCTCAGCGATAAACAGGAGAG
GTGCCATGGGTTACCAGATGGACAACAGGAACTACGGAGACCTCGGGGCCTACTGGACTCGGGGGAC
TGGAGACAAGATGTCAACACGTTCAGGAGGGCTCTGCTCAGTGGAACACCGACTCACGCTGTTTACAA
TACCACCGCAAAGAAGGAAAAAACAAAGAATTTGACCAGGCAGGTGAACAAAGGAAGCCGCATAATCC
AGTACTTACCTGCGGACGCCCGGCTCTACGAGCTGAAAGTGCTGGGCGGGCTGCACAAGTATCTGGAG
AAGTGCGGATGGAGTGTGGCGGGACAGGGCCTGTACAAGTACGGAGATCGAGTAAAGAAGTCAATGGA
CGCTACCGGCGCCGCTATAGCGGAAGACGTGGCGGGCTGGGACACAAAGATCTCCAAAGGACTGCTCA
CCCTGGAGTCCCACATGTTCACCAAGCTGGCGGAGGATGAAGAAATGGCGCGGGAGATCCACCACCTC
TACCGACTCTACGCAGATCCACACATGGTGGTCCAGAGGGAGATTGAAGGTGAAGTTCATGACGTACT
CCTTAGGGGGAGGGGGCAGGTCTCTTCCGGGAGGCAGCCCACCTACGCGGCCAACACCATCACCAACT
TCATAACGACAACATATGGAATGGCCGTGACCCTTGGTATACCTGAGGCCGACTGGCCCCGACTGGTA

AGGGACCTCACAGATGAGAGGGGAAACAGGCGTCTCCTCGTCTCGGGGGACGACAAGGTTCTTTTCTT
AAGAGGAGATGAGGCTAGGGCTTATGCCTCATCGGCCTACCGGATAAGTAACGACATGGACTGGTGA
GAAAGGACATGGCCCTGGAACAGGAATCTGAGATCATTGTGGACGTGAAAGAAATATCCTTCTGCTCC
CACAGGTACTGGCCGGTGAAGTACGGCAATGAGACTCACTACATGCCGGTCAGGGACGTGGGGGAGAT
CTTCGCCAAGGCCACCATGGCCTTGGGCGTCTACAAGGATGACATGACCCAGGAGGCGTGGGCTAGAG
TGCAAGGCCTTAACATGCTGGTGAACTACCATCACATCCCAGAATGCAGAATGTTGGCTCTGGCCATC
CTCTCGGTGACACGGATCGGCCTAAACCTGAAAGGGATCACCAAGGGCTGGATGATGAGCACCGAGTG
GCTTAGAGACGACTTAGCACCAGACACCATCCACGCTCTCATCACCGAAGGGAGAACCTCGGGATGGG
ACCAATTAGGCTACGTGGATTTTAAGGATAGGAAGGGTATCCTACTACGACCCGATGCTAGCTACAAG
AACTGGAGGAGAGACCTCCCTGGAAAAGTGCGACAACTCAGGGAGGATGGACAATACAAGGATTGGCT
ACAGAGGATGGCAGTGTTCGGTTAGAGACTAACCACACCGACACGAAACACACACAAACACACTAGAG
TAGAGCGCCCTGGAAAAACCAGGTTGCGGTGTTAGCGCCACCGTACATAAATACGACAAAACTGAATT
GGGGGAAAAAGCAAAATCTGCGGTATCTGCAGGCAGCGACTAAGGG

>MT308_ON186500
TTAAGGAATCCGCCTCAGACTCAAACGCAGACAAAACAACAACAAGACAGACAATCGTAAGAAATACA
ATGGACACAGACACCGTGATCGCTGGGTTGGCCATGGTGATGCTTATTCTGCTGGCTACGCTCCTGCA
GATGGCATCACACTACAGAGAACATGCCACCTTCATCTACACCCCAGGAGGAAAGCTGGAGGAGTATA
AAGGAGATGGGCTCAAAGTGTGGAGGTTAGAGCCCACGTTGGGGGATCCGACCGAACTGGGTAGGCAG
GCAAAGAAAGAGATGAACGCCATGACCTATGAGGAATTTGACAGAATGAAGTTCAGGGGAGTGGTGGC
CGAAGTGTACGCAGGAGATAAACCATCAAAGGGCTACGATAAACTTAGGATACTACTAGACATAATGG
ACAGACCCAAGTTGGGAACGACGGTTGACCTTTGCGCGGGCCGAGGGGGCTGGAGCCAGCTAGTTAGA
GAGCTAGAGGGACCAGGCGAAATCACTGCTGTCTCCCTATGGGAGAGAGGTAGAGAGGAATGGATGGC
TGACCCCGCTATAAGGAGAATCAATGCCAATGTGAAGAATGTCAAGCCCTGGCGTGTAGACACGTTAC
TATTCGACGGGGGGGAGACCTTCAAAAGGGACCAAAACATCATGAAGGAAGAGAGCTACAACGACGCC
CTACTGGATGTTGTGGACGCATGGATGATGCAGGCCACACCTCCCACAAACTTTGTGATAAAAATACA
GGTTCCCTATACACAAAAAGCGATGAGACTCATAGAAAAGTGGCAAGTGAAAACCAAGAAGGGAAGAC
TGGTTAGGCTGGCGGGAGACAGGCTGTCCAACACTGTGATGTACTTTATATCAGATAGGCTGGAAACG
CAGATTCGGGGAAGAATAACCAGCTTCCTCAGGGAGCTCCGGGAGCGAAGAGCGGACAGATCTCTGAC
GTCTGACCCGGCCCTGCAATACGAACGCATAGCGCCGGAGTGGACAGCAGAGGCTGTGATTGAGGGGT
GCGCCCCACTCACACCGCTGAACATGACCAGATCTATAGCAGAGATGCATATGGAATACCCACCTTTG
GGGATAACCAGATTCTTCAAAGAGCTCGGTTACAAGATCGCAAAAAAGAAGGGCTCAGAAGGCACGCG
AAGAAACAGGTTCGTGGGCCAGCTCATAGAGCCCCTAAGACGAGTGCTAGAACGACACCACCTCTTCG
GAGCATGGCAACTCACTTCGACAACACCTCGAGCCGTGTTCAATATCTTCAGAAGTAAAGTGGACAGG
GCTCCGGTGGAGCTCCACTCGCACTATCCGGGACTAAGAAAGATGTACGACATTCTCGCTGACCTCTG
GCTGGAAAGGTACGGAAGCATGAAGCGACTAACCGAAGATGAGATGGCCTCAGCGATAAACAGGAGAG
GTGCCATGGGTTACCAGATGGACAACAGGAACTACGGAGACCTCGGGGCCTACTGGGACTCGGGGGAC
TGGAGACAAGACGTCAACACGTTCAGGAGGGCTCTGCTCAGTGGAACACCGACTCACGCTGTTTACAA
CACCACCGCAAAGAAGGAAAAAACAAAGAATTTGACCAGACAGGTGAACAAAGGAAGCCGCATAATCC
AGTACTTACCTGCAGACGCCCGGCTCTATGAGCTGAAAGTGCTGGGCGGGCTGCACAAGTATCTGGAG
AAGTGCGGATGGAGTGTGGCGGGACAGGGCCTGTACAAGTACGGAGATCGAGTAAAGAAGTCAATGGA
CGCCACCGGCGCCGCTATAGCGGAAGACGTGGCGGGCTGGGACACCAAGATCTCCAAAGGACTGCTCA
CCCTGGAGTCCCATATGTTCACCAAGCTAGCGGAGGATGAAGAAATGGCGCGGGAGATCCACCACCTC
TACCGACTCTACGCAGATCCACACATGGTGGTCCAGAGGGAGATTGAAGGTGAGGTCCATGACGTACT
CCTTAGGGGGAGGGGGCAGGTCTCCTCCGGGAGGCAGCCCACCTACGCGGCCAACACCATCACCAACT
TCATAACGACAACATATGGAATGGCCGTGACCCTTGGTATACCTGAGGCCGACTGGCCCCGACTGATA
AGGGACCTCACAGATGAGAGGGGAAACAGGCGTCTCCTCGTCTCGGGGGACGACAAGGTCCTTTTCTT
AAGGGGAGATGAGGCTAGGGTTTACGCCTCGTCGGCCTACCGGATAAGTAACGACATGGACTGGTGA

GAAAGGACATGGCCCTGGAACAGGAATCTGAGATCATTGTGGACGTGAAAGAAATATCCTTCTGCTCC
CACAGGTACTGGCCGGTGAAGTACGGCAATGAGATTCACTACATGCCGGTCAGGGACGTGGGGGAGAT
CTTTGCCAAGGCCACCATGGCCTTGGGCGTCTACAAGGACGACATGACCCAGGAGGCGTGGGCTAGAG
TGCAAGGCCTTAACATGCTGGTGAACTACCATCACATTCCAGAATGCAGAATGTTGGCTCTGGCCATC
CTCTCAGTGACACGGATCGGCCTAAACCTGAAAGGGATCACCAAGGGCTGGATGATGAGCACCGAGTG
GCTTAGAGACGACTTAGCACCAGACACCATCCACGCTCTCATCACCGAAGGGAGAACCTCGGGGTGGG
ACCAATTAGGCTACGTGGATTTCAAGGATAGGAAGGGTATCCTACTACGACCTGATGCCAGCTACAAG
AACTGGAGGAGAGACCTCCCTGAAAAAGTGCGACAACTCAGGGAGGATGGACAATACAAGGACTGGTT
ACAGAGGATGGCAGTGTTTGGTTAGAGACCGATCACACCGACACGAAACACACACAAACACACTAGAG
TAGAGCGCCCTGGAAAAACCAGGTTGCGGTGTTAGCGCCACCGTGCATGAATACGACAAAACTGAATT
GGGGGAAAAAGTAAAATCTGCGGTATCTGCAGGCAGCGACTAAGGG

>MT297_ON186501
TTAAGGAATCCGCCTCAGACTCAAACGCAGACAAAACAACAACAAGACAGACAATCGTAAGAAATACA
ATGGACACAGACACCGTGATCGCTGGGTTGGCCATGGTGATGCTTATTCTGCTGGCTACGCTCCTGCA
GATGGCATCACACTACAGAGAACATGCCACCTTCATCTACACCCCAGGAGGAAAGCTGGAGGAGTATA
AAGGAGATGGGCTCAAAGTGTGGAGGTTAGAGCCCACGTTGGGGGATCCGACCGAACTGGGTAGGCAG
GCAAAGAAAGAGATGAACGCCATGACCTATGAGGAATTTGACAGAATGAAGTTCAGGGGAGTGGTGGC
CGAAGTGTACGCAGGAGATAAACCATCAAAGGGCTACGATAAACTTAGGATACTACTAGACATAATGG
ACAGACCCAAGTTGGGAACGACGGTTGACCTTTGCGCGGGCCGAGGGGGCTGGAGCCAGCTAGTTAGA
GAGCTAGAGGGACCAGGCGAAATCACTGCTGTCTCCCTATGGGAGAGAGGTAGAGAGGAATGGATGGC
TGACCCCGCTATAAGGAGAATCAATGCCAATGTGAAGAATGTCAAGCCCTGGCGTGTAGACACGTTAC
TATTCGACGGGGGGGAGACCTTCAAAAGGGACCAAAACATCATGAAGGAAGAGAGCTACAACGACGCC
CTACTGGATGTTGTGGACGCATGGATGATGCAGGCCACACCTCCCACAAACTTTGTGATAAAAATACA
GGTTCCCTATACACAAAAAGCGATGAGACTCATAGAAAAGTGGCAAGTGAAAACCAAGAAGGGAAGAC
TGGTTAGGCTGGCGGGAGACAGGCTGTCCAACACTGTGATGTACTTTATATCAGATAGGCTGGAAACG
CAGATTCGGGGAAGAATAACCAGCTTCCTCAGGGAGCTCCGGGAGCGAAGAGCGGACAGATCTCTGAC
GTCTGACCCGGCCCTGCAATACGAACGCATAGCGCCGGAGTGGACAGCAGAGGCTGTGATTGAGGGGT
GCGCCCCACTCACACCGCTGAACATGACCAGATCTATAGCAGAGATGCATATGGAATACCCACCTTTG
GGGATAACCAGATTCTTCAAAGAGCTCGGTTACAAGATCGCAAAAAAGAAGGGCTCAGAAGGCACGCG
AAGAAACAGGTTCGTGGGCCAGCTCATAGAGCCCCTAAGACGAGTGCTAGAACGACACCACCTCTTCG
GAGCATGGCAACTCACTTCGACAACACCTCGAGCCGTGTTCAATATCTTCAGAAGTAAAGTGGACAGG
GCTCCGGTGGAGCTCCACTCGCACTATCCGGGACTAAGAAAGATGTACGACATTCTCGCTGACCTCTG
GCTGGAAAGGTACGGAAGCATGAAGCGACTAACCGAAGATGAGATGGCCTCAGCGATAAACAGGAGAG
GTGCCATGGGTTACCAGATGGACAACAGGAACTACGGAGACCTCGGGGCCTACTGGGACTCGGGGGAC
TGGAGACAAGACGTCAACACGTTCAGGAGGGCTCTGCTCAGTGGAACACCGACTCACGCTGTTTACAA
CACCACCGCAAAGAAGGAAAAAACAAAGAATTTGACCAGACAGGTGAACAAAGGAAGCCGCATAATCC
AGTACTTACCTGCAGACGCCCGGCTCTATGAGCTGAAAGTGCTGGGCGGGCTGCACAAGTATCTGGAG
AAGTGCGGATGGAGTGTGGCGGGACAGGGCCTGTACAAGTACGGAGATCGAGTAAAGAAGTCAATGGA
CGCCACCGGCGCCGCTATAGCGGAAGACGTGGCGGGCTGGGACACCAAGATCTCCAAAGGACTGCTCA
CCCTGGAGTCCCATATGTTCACCAAGCTAGCGGAGGATGAAGAAATGGCGCGGGAGATCCACCACCTC
TACCGACTCTACGCAGATCCACACATGGTGGTCCAGAGGGAGATTGAAGGTGAGGTCCATGACGTACT
CCTTAGGGGGAGGGGGCAGGTCTCCTCCGGGAGGCAGCCCACCTACGCGGCCAACACCATCACCAACT
TCATAACGACAACATATGGAATGGCCGTGACCCTTGGTATACCTGAGGCCGACTGGCCCCGACTGATA
AGGGACCTCACAGATGAGAGGGGAAACAGGCGTCTCCTCGTCTCGGGGGACGACAAGGTCCTTTTCTT
AAGGGGAGATGAGGCTAGGGTTTACGCCTCGTCGGCCTACCGGATAAGTAACGACATGGGACTGGTGA
GAAAGGACATGGCCCTGGAACAGGAATCTGAGATCATTGTGGACGTGAAAGAAATATCCTTCTGCTCC
CACAGGTACTGGCCGGTGAAGTACGGCAATGAGATTCACTACATGCCGGTCAGGGACGTGGGGGAGAT

CTTTGCCAAGGCCACCATGGCCTTGGGCGTCTACAAGGACGACATGACCCAGGAGGCGTGGGCTAGAG
TGCAAGGCCTTAACATGCTGGTGAACTACCATCACATTCCAGAATGCAGAATGTTGGCTCTGGCCATC
CTCTCAGTGACACGGATCGGCCTAAACCTGAAAGGGATCACCAAGGGCTGGATGATGAGCACCGAGTG
GCTTAGAGACGACTTAGCACCAGACACCATCCACGCTCTCATCACCGAAGGGAGAACCTCGGGGTGGG
ACCAATTAGGCTACGTGGATTTCAAGGATAGGAAGGGTATCCTACTACGACCTGATGCCAGCTACAAG
AACTGGAGGAGAGACCTCCCTGAAAAAGTGCGACAACTCAGGGAGGATGGACAATACAAGGACTGGTT
ACAGAGGATGGCAGTGTTTGGTTAGAGACCGATCACACCGACACGAAACACACACAAACACACTAGAG
TAGAGCGCCCTGGAAAAACCAGGTTGCGGTGTTAGCGCCACCGTGCATGAATACGACAAAACTGAATT
GGGGGAAAAAGTAAAATCTGCGGTATCTGCAGGCAGCGACTAAGGG

>MT290_ON186502
TTAAGGAATCCGCCTCAGACTCAAACGCAGACAAAACAACAACAAGACAGACAATCGTAAGAAATACA
ATGGACACAGACACCGTGATCGCTGGGTTGGCCATGGTGATGCTTATTCTGCTGGCTACGCTCCTGCA
GATGGCATCACACTACAGAGAACATGCCACCTTCATCTACACCCCAGGAGGAAAGCTGGAGGAGTATA
AAGGAGATGGGCTCAAAGTGTGGAGGTTAGAGCCCACGTTGGGGGATCCGACCGAACTGGGTAGGCAG
GCAAAGAAAGAGATGAACGCCATGACCTATGAGGAATTTGACAGAATGAAGTTCAGGGGAGTGGTGGC
CGAAGTGTACGCAGGAGATAAACCATCAAAGGGCTACGATAAACTTAGGATACTACTAGACATAATGG
ACAGACCCAAGTTGGGAACGACGGTTGACCTTTGCGCGGGCCGAGGGGGCTGGAGCCAGCTAGTTAGA
GAGCTAGAGGGACCAGGCGAAATCACTGCTGTCTCCCTATGGGAGAGAGGTAGAGAGGAATGGATGGC
TGACCCCGCTATAAGGAGAATCAATGCCAATGTGAAGAATGTCAAGCCCTGGCGTGTAGACACGTTAC
TATTCGACGGGGGGGAGACCTTCAAAAGGGACCAAAACATCATGAAGGAAGAGAGCTACAACGACGCC
CTACTGGATGTTGTGGACGCATGGATGATGCAGGCCACACCTCCCACAAACTTTGTGATAAAAATACA
GGTTCCCTATACACAAAAAGCGATGAGACTCATAGAAAAGTGGCAAGTGAAAACCAAGAAGGGAAGAC
TGGTTAGGCTGGCGGGAGACAGGCTGTCCAACACTGTGATGTACTTTATATCAGATAGGCTGGAAACG
CAGATTCGGGGAAGAATAACCAGCTTCCTCAGGGAGCTCCGGGAGCGAAGAGCGGACAGATCTCTGAC
GTCTGACCCGGCCCTGCAATACGAACGCATAGCGCCGGAGTGGACAGCAGAGGCTGTGATTGAGGGGT
GCGCCCCACTCACACCGCTGAACATGACCAGATCTATAGCAGAGATGCATATGGAATACCCACCTTTG
GGGATAACCAGATTCTTCAAAGAGCTCGGTTACAAGATCGCAAAAAAGAAGGGCTCAGAAGGCACGCG
AAGAAACAGGTTCGTGGGCCAGCTCATAGAGCCCCTAAGACGAGTGCTAGAACGACACCACCTCTTCG
GAGCATGGCAACTCACTTCGACAACACCTCGAGCCGTGTTCAATATCTTCAGAAGTAAAGTGGACAGG
GCTCCGGTGGAGCTCCACTCGCACTATCCGGGACTAAGAAAGATGTACGACATTCTCGCTGACCTCTG
GCTGGAAAGGTACGGAAGCATGAAGCGACTAACCGAAGATGAGATGGCCTCAGCGATAAACAGGAGAG
GTGCCATGGGTTACCAGATGGACAACAGGAACTACGGAGACCTCGGGGCCTACTGGGACTCGGGGGAC
TGGAGACAAGACGTCAACACGTTCAGGAGGGCTCTGCTCAGTGGAACACCGACTCACGCTGTTTACAA
CACCACCGCAAAGAAGGAAAAAACAAAGAATTTGACCAGACAGGTGAACAAAGGAAGCCGCATAATCC
AGTACTTACCTGCAGACGCCCGGCTCTATGAGCTGAAAGTGCTGGGCGGGCTGCACAAGTATCTGGAG
AAGTGCGGATGGAGTGTGGCGGGACAGGGCCTGTACAAGTACGGAGATCGAGTAAAGAAGTCAATGGA
CGCCACCGGCGCCGCTATAGCGGAAGACGTGGCGGGCTGGGACACCAAGATCTCCAAAGGACTGCTCA
CCCTGGAGTCCCATATGTTCACCAAGCTAGCGGAGGATGAAGAAATGGCGCGGGAGATCCACCACCTC
TACCGACTCTACGCAGATCCACACATGGTGGTCCAGAGGGAGATTGAAGGTGAGGTCCATGACGTACT
CCTTAGGGGGAGGGGGCAGGTCTCCTCCGGGAGGCAGCCCACCTACGCGGCCAACACCATCACCAACT
TCATAACGACAACATATGGAATGGCCGTGACCCTTGGTATACCTGAGGCCGACTGGCCCCGACTGATA
AGGGACCTCACAGATGAGAGGGGAAACAGGCGTCTCCTCGTCTCGGGGGACGACAAGGTCCTTTTCTT
AAGGGGAGATGAGGCTAGGGTTTACGCCTCGTCGGCCTACCGGATAAGTAACGACATGGGACTGGTGA
GAAAGGACATGGCCCTGGAACAGGAATCTGAGATCATTGTGGACGTGAAAGAAATATCCTTCTGCTCC
CACAGGTACTGGCCGGTGAAGTACGGCAATGAGATTCACTACATGCCGGTCAGGGACGTGGGGGAGAT
CTTTGCCAAGGCCACCATGGCCTTGGGCGTCTACAAGGACGACATGACCCAGGAGGCGTGGGCTAGAG
TGCAAGGCCTTAACATGCTGGTGAACTACCATCACATTCCAGAATGCAGAATGTTGGCTCTGGCCATC

CTCTCAGTGACACGGATCGGCCTAAACCTGAAAGGGATCACCAAGGGCTGGATGATGAGCACCGAGTG
GCTTAGAGACGACTTAGCACCAGACACCATCCACGCTCTCATCACCGAAGGGAGAACCTCGGGGTGGG
ACCAATTAGGCTACGTGGATTTCAAGGATAGGAAGGGTATCCTACTACGACCTGATGCCAGCTACAAG
AACTGGAGGAGAGACCTCCCTGAAAAAGTGCGACAACTCAGGGAGGATGGACAATACAAGGACTGGTT
ACAGAGGATGGCAGTGTTTGGTTAGAGACCGATCACACCGACACGAAACACACACAAACACACTAGAG
TAGAGCGCCCTGGAAAAACCAGGTTGCGGTGTTAGCGCCACCGTGCATGAATACGACAAAACTGAATT
GGGGGAAAAAGTAAAATCTGCGGTATCTGCAGGCAGCGACTAAGGG

>MT304_ON186503
TTAAGGAATCCGCCTCAGACTCAAACGCAGACAAAACAACAACAAGACAGACAATCGTAAGAAATACA
ATGGACACAGACACCGTGATCGCTGGGTTGGCCATGGTGATGCTTATTCTGCTGGCTACGCTCCTGCA
GATGGCATCACACTACAGAGAACATGCCACCTTCATCTACACCCCAGGAGGAAAGCTGGAGGAGTATA
AAGGAGATGGGCTCAAAGTGTGGAGGTTAGAGCCCACGTTGGGGGATCCGACCGAACTGGGTAGGCAG
GCAAAGAAAGAGATGAACGCCATGACCTATGAGGAATTTGACAGAATGAAGTTCAGGGGAGTGGTGGC
CGAAGTGTACGCAGGAGATAAACCATCAAAGGGCTACGATAAACTTAGGATACTACTAGACATAATGG
ACAGACCCAAGTTGGGAACGACGGTTGACCTTTGCGCGGGCCGAGGGGGCTGGAGCCAGCTAGTTAGA
GAGCTAGAGGGACCAGGCGAAATCACTGCTGTCTCCCTATGGGAGAGAGGTAGAGAGGAATGGATGGC
TGACCCCGCTATAAGGAGAATCAATGCCAATGTGAAGAATGTCAAGCCCTGGCGTGTAGACACGTTAC
TATTCGACGGGGGGGAGACCTTCAAAAGGGACCAAAACATCATGAAGGAAGAGAGCTACAACGACGCC
CTACTGGATGTTGTGGACGCATGGATGATGCAGGCCACACCTCCCACAAACTTTGTGATAAAAATACA
GGTTCCCTATACACAAAAAGCGATGAGACTCATAGAAAAGTGGCAAGTGAAAACCAAGAAGGGAAGAC
TGGTTAGGCTGGCGGGAGACAGGCTGTCCAACACTGTGATGTACTTTATATCAGATAGGCTGGAAACG
CAGATTCGGGGAAGAATAACCAGCTTCCTCAGGGAGCTCCGGGAGCGAAGAGCGGACAGATCTCTGAC
GTCTGACCCGGCCCTGCAATACGAACGCATAGCGCCGGAGTGGACAGCAGAGGCTGTGATTGAGGGGT
GCGCCCCACTCACACCGCTGAACATGACCAGATCTATAGCAGAGATGCATATGGAATACCCACCTTTG
GGGATAACCAGATTCTTCAAAGAGCTCGGTTACAAGATCGCAAAAAAGAAGGGCTCAGAAGGCACGCG
AAGAAACAGGTTCGTGGGCCAGCTCATAGAGCCCCTAAGACGAGTGCTAGAACGACACCACCTCTTCG
GAGCATGGCAACTCACTTCGACAACACCTCGAGCCGTGTTCAATATCTTCAGAAGTAAAGTGGACAGG
GCTCCGGTGGAGCTCCACTCGCACTATCCGGGACTAAGAAAGATGTACGACATTCTCGCTGACCTCTG
GCTGGAAAGGTACGGAAGCATGAAGCGACTAACCGAAGATGAGATGGCCTCAGCGATAAACAGGAGAG
GTGCCATGGGTTACCAGATGGACAACAGGAACTACGGAGACCTCGGGGCCTACTGGACTCGGGGGAC
TGGAGACAAGACGTCAACACGTTCAGGAGGGCTCTGCTCAGTGGAACACCGACTCACGCTGTTTACAA
CACCACCGCAAAGAAGGAAAAAACAAAGAATTTGACCAGACAGGTGAACAAAGGAAGCCGCATAATCC
AGTACTTACCTGCAGACGCCCGGCTCTATGAGCTGAAAGTGCTGGGCGGGCTGCACAAGTATCTGGAG
AAGTGCGGATGGAGTGTGGCGGGACAGGGCCTGTACAAGTACGGAGATCGAGTAAAGAAGTCAATGGA
CGCCACCGGCGCCGCTATAGCGGAAGACGTGGCGGGCTGGGACACCAAGATCTCCAAAGGACTGCTCA
CCCTGGAGTCCCATATGTTCACCAAGCTAGCGGAGGATGAAGAAATGGCGCGGGAGATCCACCACCTC
TACCGACTCTACGCAGATCCACACATGGTGGTCCAGAGGGAGATTGAAGGTGAGGTCCATGACGTACT
CCTTAGGGGGAGGGGGCAGGTCTCCTCCGGGAGGCAGCCCACCTACGCGGCCAACACCATCACCAACT
TCATAACGACAACATATGGAATGGCCGTGACCCTTGGTATACCTGAGGCCGACTGGCCCCGACTGATA
AGGGACCTCACAGATGAGAGGGGAAACAGGCGTCTCCTCGTCTCGGGGGACGACAAGGTCCTTTTCTT
AAGGGGAGATGAGGCTAGGGTTTACGCCTCGTCGGCCTACCGGATAAGTAACGACATGGACTGGTGA
GAAAGGACATGGCCCTGGAACAGGAATCTGAGATCATTGTGGACGTGAAAGAAATATCCTTCTGCTCC
CACAGGTACTGGCCGGTGAAGTACGGCAATGAGATTCACTACATGCCGGTCAGGGACGTGGGGGAGAT
CTTTGCCAAGGCCACCATGGCCTTGGGCGTCTACAAGGACGACATGACCCAGGAGGCGTGGGCTAGAG
TGCAAGGCCTTAACATGCTGGTGAACTACCATCACATTCCAGAATGCAGAATGTTGGCTCTGGCCATC
CTCTCAGTGACACGGATCGGCCTAAACCTGAAAGGGATCACCAAGGGCTGGATGATGAGCACCGAGTG
GCTTAGAGACGACTTAGCACCAGACACCATCCACGCTCTCATCACCGAAGGGAGAACCTCGGGGTGGG

ACCAATTAGGCTACGTGGATTTCAAGGATAGGAAGGGTATCCTACTACGACCTGATGCCAGCTACAAG
AACTGGAGGAGAGACCTCCCTGAAAAAGTGCGACAACTCAGGGAGGATGGACAATACAAGGACTGGTT
ACAGAGGATGGCAGTGTTTGGTTAGAGACCGATCACACCGACACGAAACACACACAAACACACTAGAG
TAGAGCGCCCTGGAAAAACCAGGTTGCGGTGTTAGCGCCACCGTGCATGAATACGACAAAACTGAATT
GGGGGAAAAAGTAAAATCTGCGGTATCTGCAGGCAGCGACTAAGGG

>MT293_ON186504
TTAAGGAATCCGCCTCAGACTCAAACGCAGACAAAACAACAACAAGACAGACAATCGTAAGAAATACA
ATGGACACAGACACCGTGATCGCTGGGTTGGCCATGGTGATGCTTATTCTGCTGGCTATGCTCCTGCA
GATGGCATCACACTACAGAGAACATGCCACCTTCATCTACACCCCAGGAGGAAAGCTGGAGGAGTATA
AAGGAGATGGGCTCAAAGTGTGGAGGTTAGAGCCCACGTTGGGGGATCCGACCGAACTGGGTAGGCAG
GCAAAGAAAGAGATGAACGCCATGACCTATGAGGAATTTGACAGAATGAAGTTCAGGGGAGTGGTGGC
CGAAGTGTACGCAGGAGATAAACCATCAAAGGGCTACGATAAACTCAGGATACTACTAGACATAATGG
ACAGACCCAAGTTGGGAACGACGGTTGACCTTTGCGCGGGCCGAGGGGGCTGGAGCCAGCTAGTTAGA
GAGCTAGAGGGACCAGGCGACATCACTGCTGTCTCCCTATGGGAGAGAGGTAGAGAGGAATGGATGGC
TGACCCCGCTATAAGGAGGATCAATGCCAATGTGAAGAATGTCAAGCCCTGGCGTGTAGACACGTTAC
TATTCGACGGGGGGGAGACCTTCAAAAGGGACCAAAACATCATGAAGGAAGAGAGCTACAACGACGCC
CTACTGGATGTTGTGGACGCATGGATGATGCAGGCCACACCTCCCACAAACTTTGTGATAAAAATACA
GGTTCCCTATACACAAAAAGCGATGAGACTCATAGAAAAGTGGCAAGTGAAAACCAAGAAGGAAGAC
TGGTTAGGCTGGCGGGAGACAGGCTGTCCAACACCGTGATGTACTTTATATCAGATAGGCTGGAAACG
CAGATTCGGGGAAGAATAACCAGCTTCCTCAGGGAGCTCCGGGAGCGAAGAGCGGACAGATCTCTGAC
GTCTGACCCGGCCCTGCAATACGAACGCATAGCGCCGGAGTGGACAGCAGAGGCTGTGATTGAGGGGT
GCGCCCCACTCACACCGCTGAACATGACCAGATCTATAGCAGAGATGCATATGGAACACCCACCTTTG
GGGATAACCAGATTCTTCAAAGAGCTCGGTTACAAGATCGCAAAAAAGAAGGGCTCAGAAGGCACGCG
AAGAAACAGGTTCGTGGGCCAGCTCATAGAGCCCCTAAGACGAGTGCTAGAACGACACCACCTCTTCG
GAGCATGGCAACTCACTTCGACAACACCTCGAGCCGTGTTCAACATCTTCAGAAGTAAAGTGGACAGG
GCTCCGGTGGAGCTCCACTCGCACTATCCGGGACTAAGAAAGATGTACGACATTCTCGCTGACCTCTG
GCTGGAGAGGTACGGAAGCATGAAGCGACTAACCGAAGATGAGATGGCCTCAGCGATAAACAGGAGAG
GTGCCATGGGTTACCAGATGGACAACAGGAACTACGGAGACCTCGGGGCCTACTGGGACTCGGGGGAT
TGGAGACAAGACGTCAACACGTTCAGGAGGGCTCTGCTCAGCGGAACACCGACTCACGCTGTTTACAA
CACCACCGCAAAGAAGGAAAAAACAAAGAATCTGACCAGACAGGTGAACAAAGGAAGCCGCATAATCC
AGTACTTACCTGCAGACGCCCGACTCTATGAGCTGAAAGTGCTGGGCGGGCTGCACAAGTATCTGGAG
AAGTGCGGATGGAGTGTGGCGGGACAGGGCCTGTACAAGTACGGAGACCGGGTAAAGAAGTCAATGGA
CGCCACCGGCGCCGCTATAGCGGAAGACGTGGCGGGCTGGGACACTAAGATCTCCAAAGGACTACTCA
CCCTGGAGTCCCATATGTTCACCAAGCTGGCGGAGGATGAAGAAATGGCGCGGGAGATCCACCACCTC
TACCGACTCTACGCAGATCCACACATGGTGGTCCAGAGGGAGATTGAAGGTGAGGTCCATGACGTACT
CCTTAGGGGGAGGGGGCAGGTCTCCTCCGGGAGGCAGCCCACCTACGCGGCCAACACCATCACCAACT
TCATAACGACAACATATGGAATGGCCGTGACCCTTGGTATACCCGAGGCCGACTGGCCCCGACTGATA
AGGGACCTCACAGATGAGAGGGGAAACAGGCGCCTCCTCGTCTCGGGGGACGACAAGGTCCTTTTCCT
AAGAGGAGATGAGGCTAGGGTTTACGCCTCATCGGCCTACCGGATAAGTAACGACATGGGACTGGTGA
GAAAGGACATGGCCCTGGAACAGGAATCTGAGATCATTGTGGACGTGAAAGAAATATCCTTCTGCTCC
CACAGGTACTGGCCGGTGAAGTACGGCAATGAGATTCACCACATGCCGGTCAGGGACGTGGGGGAGAT
CTTTGCCAAGGCCACCATGGCCTTGGGCGTCTACAAGGATGACATGACCCAGGAGGCGTGGGCTAGAG
TGCAAGGCCTTAACATGCTGGTGAACTACCATCACATTCCAGAATGCAGAATGTTGGCTCTGGCCATC
CTCTCGGTGACACGGATCGGCCTAAACCTGAAAGGGATCACCAAGGGCTGGATGATGAGCACCGAGTG
GCTTAGAGACGACTTAGCACCAGACACCATCCACGCTCTCATCACCGAAGGGAGAACCTCGGGATGGG
ACCAATTAGGCTACGTGGATTTCAAGGATAGAAAGGGTATCCTACTACGACCTGATGCCAGCTACAAG
AACTGGAGGAGAGACCTCCCTGAAAAAGTGCGACAACTCAGGGAAGATGGACAATACAAGGACTGGCT

ACAGAGGATGGCAGTGTTTGGTTAGAGACTGATCACACCGACACGAAACACACACAAACACACTAGAG
TAGAGCGCCCTGGAAAAACCAGGTTGCGGTGTTAGCGCCACCGTACATGAATACGACAAAACTGAATT
GGGGGAAAAAGTAAAATCTGCGGTATCTGCAGGCAGCGACTAAGGG

>MT299_ON186505
TTAAGGAATCCGCCTCAGACTCAAACGCAGACAAAACAACAACAAGACAGACAATCGTAAGAAATACA
ATGGACACAGACACCGTGATCGCTGGGTTGGCCATGGTGATGCTTATTCTGCTGGCTATGCTCCTGCA
GATGGCATCACACTACAGAGAACATGCCACCTTCATCTACACCCCAGGAGGAAAGCTGGAGGAGTATA
AAGGAGATGGGCTCAAAGTGTGGAGGTTAGAGCCCACGTTGGGGGATCCGACCGAACTGGGTAGGCAG
GCAAAGAAAGAGATGAACGCCATGACCTATGAGGAATTTGACAGAATGAAGTTCAGGGGAGTGGTGGC
CGAAGTGTACGCAGGAGATAAACCATCAAAGGGCTACGATAAACTCAGGATACTACTAGACATAATGG
ACAGACCCAAGTTGGGAACGACGGTTGACCTTTGCGCGGGCCGAGGGGGCTGGAGCCAGCTAGTTAGA
GAGCTAGAGGGACCAGGCGACATCACTGCTGTCTCCCTATGGGAGAGAGGTAGAGAGGAATGGATGGC
TGACCCCGCTATAAGGAGGATCAATGCCAATGTGAAGAATGTCAAGCCCTGGCGTGTAGACACGTTAC
TATTCGACGGGGGGGAGACCTTCAAAAGGGACCAAAACATCATGAAGGAAGAGAGCTACAACGACGCC
CTACTGGATGTTGTGGACGCATGGATGATGCAGGCCACACCTCCCACAAACTTTGTGATAAAAATACA
GGTTCCCTATACACAAAAAGCGATGAGACTCATAGAAAAGTGGCAAGTGAAAACCAAGAAGGGAAGAC
TGGTTAGGCTGGCGGGAGACAGGCTGTCCAACACCGTGATGTACTTTATATCAGATAGGCTGGAAACG
CAGATTCGGGGAAGAATAACCAGCTTCCTCAGGGAGCTCCGGGAGCGAAGAGCGGACAGATCTCTGAC
GTCTGACCCGGCCCTGCAATACGAACGCATAGCGCCGGAGTGGACAGCAGAGGCTGTGATTGAGGGGT
GCGCCCCACTCACACCGCTGAACATGACCAGATCTATAGCAGAGATGCATATGGAACACCCACCTTTG
GGGATAACCAGATTCTTCAAAGAGCTCGGTTACAAGATCGCAAAAAAGAAGGGCTCAGAAGGCACGCG
AAGAAACAGGTTCGTGGGCCAGCTCATAGAGCCCCTAAGACGAGTGCTAGAACGACACCACCTCTTCG
GAGCATGGCAACTCACTTCGACAACACCTCGAGCCGTGTTCAACATCTTCAGAAGTAAAGTGGACAGG
GCTCCGGTGGAGCTCCACTCGCACTATCCGGGACTAAGAAAGATGTACGACATTCTCGCTGACCTCTG
GCTGGAGAGGTACGGAAGCATGAAGCGACTAACCGAAGATGAGATGGCCTCAGCGATAAACAGGAGAG
GTGCCATGGGTTACCAGATGGACAACAGGAACTACGGAGACCTCGGGGCCTACTGGGACTCGGGGGAT
TGGAGACAAGACGTCAACACGTTCAGGAGGGCTCTGCTCAGCGGAACACCGACTCACGCTGTTTACAA
CACCACCGCAAAGAAGGAAAAAACAAAGAATCTGACCAGACAGGTGAACAAAGGAAGCCGCATAATCC
AGTACTTACCTGCAGACGCCCGACTCTATGAGCTGAAAGTGCTGGGCGGGCTGCACAAGTATCTGGAG
AAGTGCGGATGGAGTGTGGCGGGACAGGGCCTGTACAAGTACGGAGACCGGGTAAAGAAGTCAATGGA
CGCCACCGGCGCCGCTATAGCGGAAGACGTGGCGGGCTGGGACACTAAGATCTCCAAAGGACTACTCA
CCCTGGAGTCCCATATGTTCACCAAGCTGGCGGAGGATGAAGAAATGGCGCGGGAGATCCACCACCTC
TACCGACTCTACGCAGATCCACACATGGTGGTCCAGAGGGAGATTGAAGGTGAGGTCCATGACGTACT
CCTTAGGGGGAGGGGGCAGGTCTCCTCCGGGAGGCAGCCCACCTACGCGGCCAACACCATCACCAACT
TCATAACGACAACATATGGAATGGCCGTGACCCTTGGTATACCCGAGGCCGACTGGCCCCGACTGATA
AGGGACCTCACAGATGAGAGGGGAAACAGGCGCCTCCTCGTCTCGGGGGACGACAAGGTCCTTTTCCT
AAGAGGAGATGAGGCTAGGGTTTACGCCTCATCGGCCTACCGGATAAGTAACGACATGGGACTGGTGA
GAAAGGACATGGCCCTGGAACAGGAATCTGAGATCATTGTGGACGTGAAAGAAATATCCTTCTGCTCC
CACAGGTACTGGCCGGTGAAGTACGGCAATGAGATTCACCACATGCCGGTCAGGGACGTGGGGGAGAT
CTTTGCCAAGGCCACCATGGCCTTGGGCGTCTACAAGGATGACATGACCCAGGAGGCGTGGGCTAGAG
TGCAAGGCCTTAACATGCTGGTGAACTACCATCACATTCCAGAATGCAGAATGTTGGCTCTGGCCATC
CTCTCGGTGACACGGATCGGCCTAAACCTGAAAGGGATCACCAAGGGCTGGATGATGAGCACCGAGTG
GCTTAGAGACGACTTAGCACCAGACACCATCCACGCTCTCATCACCGAAGGGAGAACCTCGGGATGGG
ACCAATTAGGCTACGTGGATTTCAAGGATAGAAAGGGTATCCTACTACGACCTGATGCCAGCTACAAG
AACTGGAGGAGAGACCTCCCTGAAAAAGTGCGACAACTCAGGGAAGATGGACAATACAAGGACTGGCT
ACAGAGGATGGCAGTGTTTGGTTAGAGACTGATCACACCGACACGAAACACACACAAACACACTAGAG

TAGAGCGCCCTGGAAAAACCAGGTTGCGGTGTTAGCGCCACCGTACATGAATACGACAAAACTGAATT
GGGGGAAAAAGTAAAATCTGCGGTATCTGCAGGCAGCGACTAAGGG

>MT328_ON186506
CGTCCTGAAACAAGTTGGGGAAAGGAAAGAATAACAAACAGAAAAGAACAAAAAAATAGAGAAAGAAA
CTGCTAAGACAAAACAAGAATGGCAAATGACACCATTGCCAACACCATCAACAATGCTGCAGAGAAGG
TTCAGGCAGTGGTCCAGAAGCTTCTGGGCGGTTCATCCTTTAACACTGTTTACTTGCTCGTGCTTGCT
GCTGCTTGGTTCTTCACAGGAGACGTCTATATCACCATCTTCAGCGCCATCACCGCTGTGGGACCGGC
TTCTAGGGCGGACGCAGCGCTCGTCCCACTCATCGCCGCAATCATCATTCGCAGACAAAACCGCATTC
TTTCGCGCGCAGCTCTCGCGCTCGCGGCAGCTTGCTATGTCGGGCTCATGCCGTCACTGGCAGCTTAG
TTCATCTGTGGGCTCCACGAGCGAGCCTGGCCCCCTCTGGGACCAGGTTCTCGAGTGGAGATTCTGGG
TGGTCCCTGAGTATGGGGTCCCCTACGGCAAAGTCGTGACCGGAATAAAGGACATCGGGCCCTGGCCG
GACCACTCTGAACCCACGGGTGTGCGATTCTGTGAGGATGCGAAATATGCCCTGCACATGAACACGAC
TTTCTACTGCTCTGACGAGAAGGCTTACGGATGGGACTGCGACGAAAAGACTCAAAACTTCCTCTCCA
CATATGAGTACAGCTTGAATGTTGATAGGAGCAGGGCCATCTGGAAGTACTCCATCTACACTAGACCG
GTGGGGCTCCAATTGGGAAACTGCACTGTCATTGACAGAGTGTATGTTGGAACCATGTGGGGCAGTCA
CAAGGAGTTGTACCCCCTGGTTGAGTTCGACGAGGACCCGCTGCCGGATGGCAGCAACAGGTATAGTG
TTAGGGTGCTTCCCAACGGGAGATGTCAACTTCTGCCAAATCAGCACGCCTCATTGGCCCTCAAGGCC
TCTGAGGTGCAAGAGCCCAAAGTGAAATATCGAGCACCATTTCACGTCTATGACCAGCTAACCCCTCA
TGGCCCCTCCATCGGGTGGTTTGACCAGGTGTGGCCCGACCCATATGCCTGCATCAGGGAAGGGCAGT
GCCAGAGCTACCCCATGAGTAGGGAGCCGTGCAGCGGAGAGAATGACGTCCAAGTGAGGCAGACTCTG
GGAGTGACTAGGGGAGGCGTGAAGCACAGGCTCACCATCAGAGTCACGGGAAGGAAAGGGCCTTGCGA
TCCAATCATAACAGCTGACAGTGGCTACCATGAGGTCACTCGGCGCCATGAGGGGAATACCCTCGTGG
TGGTTTGCGACCTCCCTCTACATCAGACTGTGGTCACAGTGAGGGCTGGAGACACCTGGAAGGAACAT
CGAATTGCCAAGGATGCTTATCAGACTATTCACGAACAGGAAGACGATTTTGGCCACATCCCTCCGCA
GTGGGTGGCTTGGCTGAAGAACTGGTTCGGGGGACTTACTTCGGGGTTAGCCCGGGCCTTTGGCCTGA
TCAGCCGTCTAGTGGATTTAGTGGTCAACAACTTGCCAACAGTGCTTGTGGCCGTTGTGCTGGCTACC
CCATTGGGTTCGGCCTTGTCCCCTGCTCTCCGGGTATTTGTCATCGTTTTAACATACACGGTCACCAG
AGTTAAGGCAGACGGTGGAACATCCGACGAGGACGTCTTGAGCGTACCTTTAGAACTCTTCTTGCACG
TGGCCATTGCCATGTTCCAGGGACGAGATGTCGCCAACCCTGTTTCGCTTGCTCTGACGGCCCTATCC
AACCTCATGCCCATGCCCATGCCACTTGTCTTCTTCACTCTGACGCATCTCCTCATGATCAACAGAGA
CTGGACCCTAGCGCTGGCAGGGGCCCTGGCCCACCGGGGGGGGCTGCCCGGCGTTGCTCTCTACCACA
TGGCCAACAGTGGCCGCCTTCGCAGGTGGGCCCAGGATCTGGCAAAAAAGGCTGAGGCGGAGGCTGTG
GAGCAGCTCCCGGAGAGTCACCCATGGTTTGACCCACCCATCCGAGGCCTTTGGCAGGATCTGGTGGG
GCTGCAGTACCACTCTCAAGGGTACGTAGCATCCACACTGCAGGCGTTCCTCAGCGCCAGCAGGGCCA
AGAGAGAGAGAAAAAGTACCGTAGGGTTGCATTCGTGAGAAAGGGAGACATCAGGTCCTTCAAAAACTTT
TTTCTTTATCCGGGAGAGTATCACAGGCTCATGCCACACCTCCTGGCCATGTCACCATCGCAGCGTAA
AGTGTTCAGAAACAACATACCTACCATCCTCACTCGTAGAGAGGGTGAGTATGGTCCACTACTTCACG
GCTTAAAAATAGCTGGATACGTGGGAGACCTAGCCGCCGCCAGCTAGGCTCCTTACACAGAGGCGGGA
ACGGAGTCTGACCCAGGAAAGGTACCGGACACTCGAACACGGAACGAATTTCCTCCTCATTTATTTTT
CCTTGTTCTATTCTTTTTTTGTTTAATTTCTTTTCCCCTCTTCCCCGCCTACCGGCTTGGGGGTTTAA
GAGGGAGCTTAATCTTATGCTATGACG

>MT308_ON186507
CGTCCTGAAACAAGTTGGGGAAAGGAAAGAATAACAAACAGAAAAGAACAAAAAAATAGAGAAAGAAA
CTGCTAAGACAAAACAAGAATGGCAAATGACACCATTGCCAATACCATCAACAATGCTGCAGAGAAGG
TTCAGGCAGTGGTCCAGAAGCTTCTGGGTGGTTCATCCTTTAACACTGTTTACCTGCTCGTGCTTGCT
GCTGCTTGGTTCTTCACAGGAGACGTCTATATCACCATCTTCAGCGCCATCACCGCTGTGGGACCGGC

TTCTAGGGCGGACGCGGCGCTCGTCCCACTCATCGCCGCAATCATCATTCGCAGACAAAACCGCATTC
TTTCGCGCGCAGCTCTCGCGCTCGCGGCAGCTTGCTATGTCGGGCTCATGCCGTCACTGGCAGCTTAG
TTCATCTGTGGGCTCCGCGAGCGAACCTGGCCCCCTCTGGGACCAGGTTCTCGAGTGGAGATTCTGGG
TGGTCCCTGAGTATGGGGTCCCCTACGGCAAAGTCGTGACCGGAATAAAGGACATCGGGCCCTGGCCG
GACCACTCTGAACCCACGGGTGTGCGATTCTGTGAGGATGCAAAATATGCCCTGCACATGAACACGAC
TTTCTACTGCTCTGACGAGAAGGCTTACGGATGGGACTGCGACGAAAAGACTCAAAACTTCCTCTCCA
CATATGAGTACAGCTTGAATGTTGATAGGAGCAGGGCCATCTGGAAGTACTCCATCTACACTAGACCG
GTGGGGCTCCAACTGGGAAACTGCACTGTCATTGACAGAGTGTATGTTGGAACCATGTGGGGCAGCCA
CAAGGAGTTGTACCCCCTGGTTGAGTTCGACGAGGACCCGCTGCCGGATGGCAGCAACAGGTATAGTG
TTAGGGTGCTTCCCAACGGGAGATGTCAACTTCTGCCAAACCAGCACGCCTCATTGGCCCTCAAGGCC
TCTGAGGTGCAAGAGCCCAAAGTGAAATATCGAGCACCATTTCATGTCTATGACCAGCTAACCCCTCA
TGGCCCCTCCATCGGGTGGTTTGACCAGGTGTGGCCCGACCCATATGCTTGCATTAGGGAAGGGCAGT
GCCAGAGCTACCCCATGAGTAGGGAGCCGTGCAGCGGAGAGAATGATGTCCAAATGAGGCAGACTCTG
GGAGTGACTAGGGGAGGCGTGAAGCACAGGCTTACCATCAGAGTCACGGGAAGGAGAGGGCCTTGCGA
TCCAATCATAACAGCTGACAGTGGCTACCATGAGGTCACCCGGCGCCATGAAGGGAACACCCTCGTGG
TGATTTGCGACCTCCCTCTACATCAGACTGTGGTCACAGTGAGGGCCGGAGACACCTGGAAGGAACAT
CGAATTGCCAAGGATGCTTATCAGACTATTCACGAACAGGAAGACGATTTTGGCCACATCCCTCCGCA
GTGGGTGGCTTGGCTGAAGAACTGGTTCGGGGGACTCACCTCGGGGCTAGCCCGGGCCTTTGGCCTGA
TCAGCCGTCTAGCGGATTTAGTGGTCAACAACTTGCCAACAGTGCTTGTGGCCGTGGTGCTGGCTACC
CCATTGGGTTCGGCCTTGTCCCCTGCTCTCCGGGTCTTTGTCATCGTCCTAACATACACGGTCACCAG
AGCCAAGGCAGACGGTGGAACATCCGACGAGGACGTCTTGAGCGTACCTCTAGAACTCTTCTTGCACG
TGGCCATTGCCATGTTCCAGGGACGAGATGTCGCCAACCCTGTTTCGCTTGCTTTGACGGCCCTATCC
AACCTCGTGCCCATGCCCATGCCACTTGTCTTCTTCACTCTGACGCATCTCCTCATGATCAACAGAGA
CTGGACCCTAGCGTTGGCAGGGGCCCTGGCCCACCGGGGGGGGCTGCCCGGCGTTGCTCTCTACCACA
TGGTCAACAGTGGCCGCCTTCGCAGGTGGGCCCAGGATCTGGCAAAAAAGGCTGAGGCGGAGGCTGTG
GAGCAGCTCCCGGAGAGCCACCCATGGTTTGACCCACCCATCCGAGGCCTTTGGCAAGATCTGGTGGG
GCTGCAGTACCATTCCCAAGGGTACGTAGCATCCACACTGCAGGCGTTCCTCAGCGCCAGCAGGGCCA
AGAGAGAAAAAAAATACCGTAGGGTCGCATTCGTGAGAAAGGGAGACATCAGGTCCTTCAAAAACTTT
TTTCTTTATCCGGGAGAGTATCACAGGCTCATGCCACACCTCCTGGCCATGTCACCATCGCAGCGTAA
AGTGTTCAGAAATAACATACCCACCATCCTCACTCGTAGAGAGGGTGAGTATGGTCCACTACTTCACG
GCTTAAAAATAGCTGGATATGTGGGAGACCTAGCCGCCGCCAGCTAGGCTCCTTACACAGAGGCGGGA
ACGGAGTCTGACCTAGGAAAGGTACCGGACACTCGAACACGGAACGAATTTCCTCCTCATTTATTTTC
CCTTGTTCTATTCTTTTTTGTTTAATTTCTTTTCCCCTCTTCCCCGCCTACCGGCTTGGGGGTTTAAG
AGGGAGCTCAATCTTATGCTATGACG

>MT304_ON186508
CGTCCTGAAACAAGTTGGGGAAAGGAAAGAATAACAAACAGAAAAGAACAAAAAAATAGAGAAAGAAA
CTGCTAAGACAAAACAAGAATGGCAAATGACACCATTGCCAATACCATCAACAATGCTGCAGAGAAGG
TTCAGGCAGTGGTCCAGAAGCTTCTGGGTGGTTCATCCTTTAACACTGTTTACCTGCTCGTGCTTGCT
GCTGCTTGGTTCTTCACAGGAGACGTCTATATCACCATCTTCAGCGCCATCACCGCTGTGGGACCGGC
TTCTAGGGCGGACGCGGCGCTCGTCCCACTCATCGCCGCAATCATCATTCGCAGACAAAACCGCATTC
TTTCGCGCGCAGCTCTCGCGCTCGCGGCAGCTTGCTATGTCGGGCTCATGCCGTCACTGGCAGCTTAG
TTCATCTGTGGGCTCCGCGAGCGAACCTGGCCCCCTCTGGGACCAGGTTCTCGAGTGGAGATTCTGGG
TGGTCCCTGAGTATGGGGTCCCCTACGGCAAAGTCGTGACCGGAATAAAGGACATCGGGCCCTGGCCG
GACCACTCTGAACCCACGGGTGTGCGATTCTGTGAGGATGCAAAATATGCCCTGCACATGAACACGAC
TTTCTACTGCTCTGACGAGAAGGCTTACGGATGGGACTGCGACGAAAAGACTCAAAACTTCCTCTCCA
CATATGAGTACAGCTTGAATGTTGATAGGAGCAGGGCCATCTGGAAGTACTCCATCTACACTAGACCG
GTGGGGCTCCAACTGGGAAACTGCACTGTCATTGACAGAGTGTATGTTGGAACCATGTGGGGCAGCCA

CAAGGAGTTGTACCCCCTGGTTGAGTTCGACGAGGACCCGCTGCCGGATGGCAGCAACAGGTATAGTG
TTAGGGTGCTTCCCAACGGGAGATGTCAACTTCTGCCAAACCAGCACGCCTCATTGGCCCTCAAGGCC
TCTGAGGTGCAAGAGCCCAAAGTGAAATATCGAGCACCATTTCATGTCTATGACCAGCTAACCCCTCA
TGGCCCCTCCATCGGGTGGTTTGACCAGGTGTGGCCCGACCCATATGCTTGCATTAGGGAAGGGCAGT
GCCAGAGCTACCCCATGAGTAGGGAGCCGTGCAGCGGAGAGAATGATGTCCAAATGAGGCAGACTCTG
GGAGTGACTAGGGGAGGCGTGAAGCACAGGCTTACCATCAGAGTCACGGGAAGGAGAGGGCCTTGCGA
TCCAATCATAACAGCTGACAGTGGCTACCATGAGGTCACCCGGCGCCATGAAGGGAACACCCTCGTGG
TGATTTGCGACCTCCCTCTACATCAGACTGTGGTCACAGTGAGGGCCGGAGACACCTGGAAGGAACAT
CGAATTGCCAAGGATGCTTATCAGACTATTCACGAACAGGAAGACGATTTTGGCCACATCCCTCCGCA
GTGGGTGGCTTGGCTGAAGAACTGGTTCGGGGGACTCACCTCGGGGCTAGCCCGGGCCTTTGGCCTGA
TCAGCCGTCTAGCGGATTTAGTGGTCAACAACTTGCCAACAGTGCTTGTGGCCGTGGTGCTGGCTACC
CCATTGGGTTCGGCCTTGTCCCCTGCTCTCCGGGTCTTTGTCATCGTCCTAACATACACGGTCACCAG
AGCCAAGGCAGACGGTGGAACATCCGACGAGGACGTCTTGAGCGTACCTCTAGAACTCTTCTTGCACG
TGGCCATTGCCATGTTCCAGGGACGAGATGTCGCCAACCCTGTTTCGCTTGCTTTGACGGCCCTATCC
AACCTCGTGCCCATGCCCATGCCACTTGTCTTCTTCACTCTGACGCATCTCCTCATGATCAACAGAGA
CTGGACCCTAGCGTTGGCAGGGGCCCTGGCCCACCGGGGGGGGCTGCCCGGCGTTGCTCTCTACCACA
TGGTCAACAGTGGCCGCCTTCGCAGGTGGGCCCAGGATCTGGCAAAAAAGGCTGAGGCGGAGGCTGTG
GAGCAGCTCCCGGAGAGCCACCCATGGTTTGACCCACCCATCCGAGGCCTTTGGCAAGATCTGGTGGG
GCTGCAGTACCATTCCCAAGGGTACGTAGCATCCACACTGCAGGCGTTCCTCAGCGCCAGCAGGGCCA
AGAGAGAAAAAAAATACCGTAGGGTCGCATTCGTGAGAAAGGGAGACATCAGGTCCTTCAAAAACTTT
TTTCTTTATCCGGGAGAGTATCACAGGCTCATGCCACACCTCCTGGCCATGTCACCATCGCAGCGTAA
AGTGTTCAGAAATAACATACCCACCATCCTCACTCGTAGAGAGGGTGAGTATGGTCCACTACTTCACG
GCTTAAAAATAGCTGGATATGTGGGAGACCTAGCCGCCGCCAGCTAGGCTCCTTACACAGAGGCGGGA
ACGGAGTCTGACCTAGGAAAGGTACCGGACACTCGAACACGGAACGAATTTCCTCCTCATTTATTTTC
CCTTGTTCTATTCTTTTTTGTTTAATTTCTTTTCCCCTCTTCCCCGCCTACCGGCTTGGGGGTTTAAG
AGGGAGCTCAATCTTATGCTATGACG

>MT297_ON186509
CGTCCTGAAACAAGTTGGGGAAAGGAAAGAATAACAAACAGAAAAGAACAAAAAAATAGAGAAAGAAA
CTGCTAAGACAAAACAAGAATGGCAAATGACACCATTGCCAATACCATCAACAATGCTGCAGAGAAGG
TTCAGGCAGTGGTCCAGAAGCTTCTGGGTGGTTCATCCTTTAACACTGTTTACCTGCTCGTGCTTGCT
GCTGCTTGGTTCTTCACAGGAGACGTCTATATCACCATCTTCAGCGCCATCACCGCTGTGGGACCGGC
TTCTAGGGCGGACGCGGCGCTCGTCCCACTCATCGCCGCAATCATCATTCGCAGACAAAACCGCATTC
TTTCGCGCGCAGCTCTCGCGCTCGCGGCAGCTTGCTATGTCGGGCTCATGCCGTCACTGGCAGCTTAG
TTCATCTGTGGGCTCCGCGAGCGAACCTGGCCCCCTCTGGGACCAGGTTCTCGAGTGGAGATTCTGGG
TGGTCCCTGAGTATGGGGTCCCCTACGGCAAAGTCGTGACCGGAATAAAGGACATCGGGCCCTGGCCG
GACCACTCTGAACCCACGGGTGTGCGATTCTGTGAGGATGCAAAATATGCCCTGCACATGAACACGAC
TTTCTACTGCTCTGACGAGAAGGCTTACGGATGGGACTGCGACGAAAAGACTCAAAACTTCCTCTCCA
CATATGAGTACAGCTTGAATGTTGATAGGAGCAGGGCCATCTGGAAGTACTCCATCTACACTAGACCG
GTGGGGCTCCAACTGGGAAACTGCACTGTCATTGACAGAGTGTATGTTGGAACCATGTGGGGCAGCCA
CAAGGAGTTGTACCCCCTGGTTGAGTTCGACGAGGACCCGCTGCCGGATGGCAGCAACAGGTATAGTG
TTAGGGTGCTTCCCAACGGGAGATGTCAACTTCTGCCAAACCAGCACGCCTCATTGGCCCTCAAGGCC
TCTGAGGTGCAAGAGCCCAAAGTGAAATATCGAGCACCATTTCATGTCTATGACCAGCTAACCCCTCA
TGGCCCCTCCATCGGGTGGTTTGACCAGGTGTGGCCCGACCCACATGCTTGCATTAGGGAAGGGCAGT
GCCAGAGCTACCCCATGAGTAGGGAGCCGTGCAGCGGAGAGAATGATGTCCAAATGAGGCAGACTCTG
GGAGTGACTAGGGGAGGCGTGAAGCACAGGCTTACCATCAGAGTCACGGGAAGGAGAGGGCCTTGCGA
TCCAATCATAACAGCTGACAGTGGCTACCATGAGGTCACCCGGCGCCATGAAGGGAACACCCTCGTGG
TGATTTGCGACCTCCCTCTACATCAGACTGTGGTCACAGTGAGGGCCGGAGACACCTGGAAGGAACAT

CGAATTGCCAAGGATGCTTATCAGACTATTCACGAACAGGAAGACGATTTTGGCCACATCCCTCCGCA
GTGGGTGGCTTGGCTGAAGAACTGGTTCGGGGGACTCACCTCGGGGCTAGCCCGGGCCTTTGGCCTGA
TCAGCCGTCTAGCGGATTTAGTGGTCAACAACTTGCCAACAGTGCTTGTGGCCGTGGTGCTGGCTACC
CCATTGGGTTCGGCCTTGTCCCCTGCTCTCCGGGTCTTTGTCATCGTCCTAACATACACGGTCACCAG
AGCCAAGGCAGACGGTGGAACATCCGACGAGGACGTCTTGAGCGTACCTCTAGAACTCTTCTTGCACG
TGGCCATTGCCATGTTCCAGGGACGAGATGTCGCCAACCCTGTTTCGCTTGCTTTGACGGCCCTATCC
AACCTCGTGCCCATGCCCATGCCACTTGTCTTCTTCACTCTGACGCATCTCCTCATGATCAACAGAGA
CTGGACCCTAGCGTTGGCAGGGGCCCTGGCCCACCGGGGGGGGCTGCCCGGCGTTGCTCTCTACCACA
TGGTCAACAGTGGCCGCCTTCGCAGGTGGGCCCAGGATCTGGCAAAAAAGGCTGAGGCGGAGGCTGTG
GAGCAGCTCCCGGAGAGCCACCCATGGTTTGACCCACCCATCCGAGGCCTTTGGCAAGATCTGGTGGG
GCTGCAGTACCATTCCCAAGGGTACGTAGCATCCACACTGCAGGCGTTCCTCAGCGCCAGCAGGGCCA
AGAGAGAAAAAAAATACCGTAGGGTCGCATTCGTGAGAAAGGGAGACATCAGGTCCTTCAAAAACTTT
TTTCTTTATCCGGGAGAGTATCACAGGCTCATGCCACACCTCCTGGCCATGTCACCATCGCAGCGTAA
AGTGTTCAGAAATAACATACCCACCATCCTCACTCGTAGAGAGGGTGAGTATGGTCCGCTACTTCACG
GCTTAAAAATAGCTGGATATGTGGGAGACCTAGCCGCCGCCAGCTAGGCTCCTTACACAGAGGCGGGA
ACGGAGTCTGACCTAGGAAAGGTACCGGACACTCGAACACGGAACGAATTTCCTCCTCATTTATTTTC
CCTTGTTCTATTCTTTTTTGTTTAATTTCTTTTCCCCTCTTCCCCGCCTACCGGCTTGGGGGTTTAAG
AGGGAGCTCAATCTTATGCTATGACG

>MT290_ON186510
CGTCCTGAAACAAGTTGGGGAAAGGAAAGAATAACAAACAGAAAAGAACAAAAAAATAGAGAAAGAAA
CTGCTAAGACAAAACAAGAATGGCAAATGACACCATTGCCAATACCATCAACAATGCTGCAGAGAAGG
TTCAGGCAGTGGTCCAGAAGCTTCTGGGTGGTTCATCCTTTAACACTGTTTACCTGCTCGTGCTTGCT
GCTGCTTGGTTCTTCACAGGAGACGTCTATATCACCATCTTCAGCGCCATCACCGCTGTGGGACCGGC
TTCTAGGGCGGACGCGGCGCTCGTCCCACTCATCGCCGCAATCATCATTCGCAGACAAAACCGCATTC
TTTCGCGCGCAGCTCTCGCGCTCGCGGCAGCTTGCTATGTCGGGCTCATGCCGTCACTGGCAGCTTAG
TTCATCTGTGGGCTCCGCGAGCGAACCTGGCCCCCTCTGGGACCAGGTTCTCGAGTGGAGATTCTGGG
TGGTCCCTGAGTATGGGGTCCCCTACGGCAAAGTCGTGACCGGAATAAAGGACATCGGGCCCTGGCCG
GACCACTCTGAACCCACGGGTGTGCGATTCTGTGAGGATGCAAAATATGCCCTGCACATGAACACGAC
TTTCTACTGCTCTGACGAGAAGGCTTACGGATGGGACTGCGACGAAAAGACTCAAAACTTCCTCTCCA
CATATGAGTACAGCTTGAATGTTGATAGGAGCAGGGCCATCTGGAAGTACTCCATCTACACTAGACCG
GTGGGGCTCCAACTGGGAAACTGCACTGTCATTGACAGAGTGTATGTTGGAACCATGTGGGGCAGCCA
CAAGGAGTTGTACCCCCTGGTTGAGTTCGACGAGGACCCGCTGCCGGATGGCAGCAACAGGTATAGTG
TTAGGGTGCTTCCCAACGGGAGATGTCAACTTCTGCCAAACCAGCACGCCTCATTGGCCCTCAAGGCC
TCTGAGGTGCAAGAGCCCAAAGTGAAATATCGAGCACCATTTCATGTCTATGACCAGCTAACCCCTCA
TGGCCCCTCCATCGGGTGGTTTGACCAGGTGTGGCCCGACCCATATGCTTGCATTAGGGAAGGGCAGT
GCCAGAGCTACCCCATGAGTAGGGAGCCGTGCAGCGGAGAGAATGATGTCCAAATGAGGCAGACTCTG
GGAGTGACTAGGGGAGGCGTGAAGCACAGGCTTACCATCAGAGTCACGGGAAGGAGAGGGCCTTGCGA
TCCAATCATAACAGCTGACAGTGGCTACCATGAGGTCACCCGGCGCCATGAAGGGAACACCCTCGTGG
TGATTTGCGACCTCCCTCTACATCAGACTGTGGTCACAGTGAGGGCCGGAGACACCTGGAAGGAACAT
CGAATTGCCAAGGATGCTTATCAGACTATTCACGAACAGGAAGACGATTTTGGCCACATCCCTCCGCA
GTGGGTGGCTTGGCTGAAGAACTGGTTCGGGGGACTCACCTCGGGGCTAGCCCGGGCCTTTGGCCTGA
TCAGCCGTCTAGCGGATTTAGTGGTTAACAACTTGCCAACAGTGCTTGTGGCCGTGGTGCTGGCTACC
CCATTGGGTTCGGCCTTGTCCCCTGCTCTCCGGGTCTTTGTCATCGTCCTAACATACACGGTCACCAG
AGCCAAGGCAGACGGTGGAACATCCGACGAGGACGTCTTGAGCGTACCTCTAGAACTCTTCTTGCACG
TGGCCATTGCCATGTTCCAGGGACGAGATGTCGCCAACCCTGTTTCGCTTGCTTTGACGGCCCTATCC
AACCTCGTGCCCATGCCCATGCCACTTGTCTTCTTCACTCTGACGCATCTCCTCATGATCAACAGAGA
CTGGACCCTAGCGTTGGCAGGGGCCCTGGCCCACCGGGGGGGGCTGCCCGGCGTTGCTCTCTACCACA

TGGTCAACAGTGGCCGCCTTCGCAGGTGGGCCCAGGATCTGGCAAAAAAGGCTGAGGCGGAGGCTGTG
GAGCAGCTCCCGGAGAGCCACCCATGGTTTGACCCACCCATCCGAGGCCTTTGGCAAGATCTGGTGGG
GCTGCAGTACCATTCCCAAGGGTACGTAGCATCCACACTGCAGGCGTTCCTCAGCGCCAGCAGGGCCA
AGAGAGAAAAAAAATACCGTAGGGTCGCATTCGTGAGAAAGGGAGACATCAGGTCCTTCAAAAACTTT
TTTCTTTATCCGGGAGAGTATCACAGGCTCATGCCACACCTCCTGGCCATGTCACCATCGCAGCGTAA
AGTGTTCAGAAATAACATACCCACCATCCTCACTCGTAGAGAGGGTGAGTATGGTCCACTACTTCACG
GCTTAAAAATAGCTGGATATGTGGGAGACCTAGCCGCCGCCAGCTAGGCTCCTTACACAGAGGCGGGA
ACGGAGTCTGACCTAGGAAAGGTACCGGACACTCGAACACGGAACGAATTTCCTCCTCATTTATTTTC
CCTTGTTCTATTCTTTTTTGTTTAATTTCTTTTCCCCTCTTCCCCGCCTACCGGCTTGGGGGTTTAAG
AGGGAGCTCAATCTTATGCTATGACG

>MT293_ON186511
CGTCCTGAAACAAGTTGGGGAAAGGAATAAATAACAAACAGAAAAGAACAAAAAAATAGAGAAAGAAA
CTGCTAAGACAAAACAAGAATGGCAAATGACACCATTGCCAATACCATCAACAATGCTGCAGAGAAGG
TGCAGGCAGTGGTCCAGAAGCTTCTGGGCGGTTCATCCTTTAACACTGTTTACCTACTCGTGCTTGCT
GCTGCTTGGTTCTTCACTGGAGACGTCTACATCATCATCTTCAGCGCCATCACCGCTGTGGGACCGGC
TTCTAGGGCGGACGCGGCGCTCGTCCCCCTCATCGCCGCAATCATCATTCGCAGACAAAACCGCATTC
TTTCGCGCGCAGCTCTCGCGCTCGCGGCAGCTTGCTACGTCGGGCTCATGCCGTCACTGGCAGCTTAG
TTCATCTGTGGGCTCCGCGAGTGAGCCTGGCCCCCTCTGGGACCAGGCTCTCGAGTGGAGGTTCTGGG
TGGTCCCTGAGTATGGGGTCCCCTACGGCAAAGTTGTGACCGGAATAAAAGACATCGGGCCCTGGCCG
GACTACTCTGAACCCACAGGCGTGCGATTCTGTGAGGATGCAAAATATGCCCTACACATGAACACGAC
TTTCCATTGCTCTGATGAGAAGGCCTATGGATGGGACTGTGACGAAAAGACTCAAACCTTCCTCTCCA
CATATGAGTACAGCTTGAATGTGGATAGGAGTAGGGCTATCTGGAAGTACTCCATCTACACCAGACCG
GTAGGGCTCCAACTGGGAAACTGCACTGTCACCGACAGAGTGTATGTTGGAACCATGTGGGGCAGCCA
CAAGGAATTGTACCCCCTGGTTGAGTTTGATGAGGACCCGCTGCCGGATGGCAGTAACAGATACAGTG
TTAGAGTGCTCCCCAACGGGAGATGCCAACTTCTGCCAAACCAGCATGCCTCGTTGGCTTTTAGGGCC
TCCGAGGTCCTAGAGCCCAAGGTGAAATATCGAGCACCATTCCATGTCTATGACCAGCTAACCCCTCA
TGGCCCCTCTATCGGGTGGTTCGACCAGGCGTGGCCCGACCCTTATGCTTGCATCAGGGAAGGGCAGT
GTCAGAGCTACCCCATGAGCAGGGAGCCGTGTAGCGGAGAGAACGAAGTCCAAATGAGGCAAACTCTG
GGAGTGACTAGGGGAGGTGTGAAGCATAGGCTCACCATCAGGGTCACGGGAAGGAGAGGGCCTTGCGA
TCCAATCATAACAGCTGACAGTGGCTACCATGAGGTCACCCGGCGCCATGAGGGGAACACCCTCGTGG
TGGTTTGTGACCTCCCTTTACATCAGACTGTGGTCACAGTGAGGGCCGGAGACACCTGGAGGGAACAC
CGAATCGTTAAGGATGTGTACCAGACTATCCACGAACAGGAAGACGATTTTGGCCATATCCCCCCGCA
ATGGGTGGCCTGGCTGAAGAACTGGTTTGGGGGGCTCACCTCGGGATTGGCCCGGGCATTCGGCCTAA
TCAGCCGTCTAGTGGATCTAGTGGTCAACAATTTACCAACAGTACTTGTGGCCGTGGTGCTGGCCACC
CCATTGGGTTCGGCCTTGTCCCCTGCTCTCAGGGTTTTCGTCATCGTCCTAACGTACACAGTTACCAG
GGCCAAGGCAGATGGGGGAACATCTGACGGGGACGTCTTGAGCGTACCCCTGGAACTCTTCTTGCACG
TGGCTATTGCTATGTTCCAGGGACGGGACGTCGCTAACCCTGTCTCGCTGGCTCTGACGGCCCTATCC
AACCTCATGCCCATGCCCATGCCACTTGTCTTCTTCACTCTGACGCATCTCCTCATAGTCAACAGAGA
CTGGACCCTAGCGCTGGCAGGGGCCCTGGCCCACCGGGGGGGGCTGCCCGGCGTCGCTCTCTACCACA
TGGTCAACAGTGGTCGCCTCCGCAGGCTGGGCCAGGATTTGGCAAAAAAGGCTGAGGCGGAGGCTGTG
GAACAGCTCCCAGAGAGCCACCCGTGGTTTGACCCACCTATTCGAGGCCTCTGGCAGGACCTGGTGGG
GCTGCAGTACCACTCTCAAGGGTATGTAGCATCCACACTGCAGGCGTTTCTCAGCGCCAGCAGGGCCA
AGAGAGAGAAAAAGTACCGCAGGGTCGCATTTGTGAGAAAGGGAGACATCAGGTCCTTTAAAAACTTT
TTTCTTTATCCGGGAGAGTACCACAGACTCATGCCACATCTCCTAGCTATGTCACCATCACAGCGTAA
AGTGTTCAGAAACAACATACCTACCATCCTCACTCGTAGAGAGGGTGAGTATGGTCCACTACTTCACG
GCCTAAAAATAGCTGGATACGTGGGAGACCTGGCCGCCGCCAGCTAGGCTCCTAACACAAAGGCGGGA
ACGGAACTCGACCCAGGAAAGGCACCAGACACTCGGACGCGGAACGGTCTTCCTCCTCATTTATTTTT

TCTTGTTCTATTCTTTTTTGTTTAACTTCTTTTCCCCTCTTCCCCACCTACTGGCTTGGGGGCTTAAG
AGGGAGCTTAGTCTTATGCTATGACG

>MT299_ON186512
CGTCCTGAAACAAGTTGGGGAAAGGAATAAATAACAAACAGAAAAGAACAAAAAAATAGAGAAAGAAA
CTGCTAAGACAAAACAAGAATGGCAAATGACACCATTGCCAATACCATCAACAATGCTGCAGAGAAGG
TGCAGGCAGTGGTCCAGAAGCTTCTGGGCGGTTCATCCTTTAACACTGTTTACCTACTCGTGCTTGCT
GCTGCTTGGTTCTTCACTGGAGACGTCTACATCATCATCTTCAGCGCCATCACCGCTGTGGGACCGGC
TTCTAGGGCGGACGCGGCGCTCGTCCCCCTCATCGCCGCAATCATCATTCGCAGACAAAACCGCATTC
TTTCGCGCGCAGCTCTCGCGCTCGCGGCAGCTTGCTACGTCGGGCTCATGCCGTCACTGGCAGCTTAG
TTCATCTGTGGGCTCCGCGAGTGAGCCTGGCCCCCTCTGGGACCAGGCTCTCGAGTGGAGGTTCTGGG
TGGTCCCTGAGTATGGGGTCCCCTACGGCAAAGTTGTGACCGGAATAAAAGACATCGGGCCCTGGCCG
GACTACTCTGAACCCACAGGCGTGCGATTCTGTGAGGATGCAAAATATGCCCTACACATGAACACGAC
TTTCCATTGCTCTGATGAGAAGGCCTATGGATGGGACTGTGACGAAAAGACTCAAACCTTCCTCTCCA
CATATGAGTACAGCTTGAATGTGGATAGGAGTAGGGCTATCTGGAAGTACTCCATCTACACCAGACCG
GTAGGGCTCCAACTGGGAAACTGCACTGTCACCGACAGAGTGTATGTTGGAACCATGTGGGGCAGCCA
CAAGGAATTGTACCCCCTGGTTGAGTTTGATGAGGACCCGCTGCCGGATGGCAGTAACAGATACAGTG
TTAGAGTGCTCCCCAACGGGAGATGCCAACTTCTGCCAAACCAGCATGCCTCGTTGGCTTTTAGGGCC
TCCGAGGTCCTAGAGCCCAAGGTGAAATATCGAGCACCATTCCATGTCTATGACCAGCTAACCCCTCA
TGGCCCCTCTATCGGGTGGTTCGACCAGGCGTGGCCCGACCCTTATGCTTGCATCAGGGAAGGGCAGT
GTCAGAGCTACCCCATGAGCAGGGAGCCGTGTAGCGGAGAGAACGAAGTCCAAATGAGGCAAACTCTG
GGAGTGACTAGGGGAGGTGTGAAGCATAGGCTCACCATCAGGGTCACGGGAAGGAGAGGGCCTTGCGA
TCCAATCATAACAGCTGACAGTGGCTACCATGAGGTCACCCGGCGCCATGAGGGGAACACCCTCGTGG
TGGTTTGTGACCTCCCTTTACATCAGACTGTGGTCACAGTGAGGGCCGGAGACACCTGGAGGGAACAC
CGAATCGTTAAGGATGTGTACCAGACTATCCACGAACAGGAAGACGATTTTGGCCATATCCCCCCGCA
ATGGGTGGCCTGGCTGAAGAACTGGTTTGGGGGGCTCACCTCGGGATTGGCCCGGGCATTCGGCCTAA
TCAGCCGTCTAGTGGATCTAGTGGTCAACAATTTACCAACAGTACTTGTGGCCGTGGTGCTGGCCACC
CCATTGGGTTCGGCCTTGTCCCCTGCTCTCAGGGTTTTCGTCATCGTCCTAACGTACACAGTTACCAG
GGCCAAGGCAGATGGGGGAACATCTGACGGGGACGTCTTGAGCGTACCCCTGGAACTCTTCTTGCACG
TGGCTATTGCTATGTTCCAGGGACGGGACGTCGCTAACCCTGTCTCGCTGGCTCTGACGGCCCTATCC
AACCTCATGCCCATGCCCATGCCACTTGTCTTCTTCACTCTGACGCATCTCCTCATAGTCAACAGAGA
CTGGACCCTAGCGCTGGCAGGGGCCCTGGCCCACCGGGGGGGGCTGCCCGGCGTCGCTCTCTACCACA
TGGTCAACAGTGGTCGCCTCCGCAGGCTGGGCCAGGATTTGGCAAAAAAGGCTGAGGCGGAGGCTGTG
GAACAGCTCCCAGAGAGCCACCCGTGGTTTGACCCACCTATTCGAGGCCTCTGGCAGGACCTGGTGGG
GCTGCAGTACCACTCTCAAGGGTATGTAGCATCCACACTGCAGGCGTTTCTCAGCGCCAGCAGGGCCA
AGAGAGAGAAAAAGTACCGCAGGGTCGCATTTGTGAGAAAGGGAGACATCAGGTCCTTTAAAAACTTT
TTTCTTTATCCGGGAGAGTACCACAGACTCATGCCACATCTCCTAGCTATGTCACCATCACAGCGTAA
AGTGTTCAGAAACAACATACCTACCATCCTCACTCGTAGAGAGGGTGAGTATGGTCCACTACTTCACG
GCCTAAAAATAGCTGGATACGTGGGAGACCTGGCCGCCGCCAGCTAGGCTCCTAACACAAAGGCGGGA
ACGGAACTCGACCCAGGAAAGGCACCAGACACTCGGACGCGGAACGGTCTTCCTCCTCATTTATTTTT
TCTTGTTCTATTCTTTTTTGTTTAACTTCTTTTCCCCTCTTCCCCACCTACTGGCTTGGGGGCTTAAG
AGGGAGCTTAGTCTTATGCTATGACG

>MT328_ON186513
AAACAGAAAAAACTCACAAGTGATTGTTGAGAACTTGACATGGCTACCCCAGTGGTAGCAGCCTTGAG
CTGCGTATTGGCCGCAGTGGACAAGGGACTGGCTCCCAATGTGGGGGCCAAGGGTCTCTTAGCTATGG
CGGCAGCATCCGCGTTGGCACCGGAATTGGTGCTGGCTGCAGCTTGGCTCTCCATACTGAAAGGAAAC
TCACCAGGTCGGGAAATGAACTACACACAAAGACACTTCATGGAGCTGCTGCCCACCATCGGGTGGCT

ATCTCTGGAATACGCGGCTCCACTGGATATCGTCCTAAACGAAGAAGACACACCACACCAGGAGGACA
GAAGACTATATGAGAAGGCACTGAACCTCTCCTACATAGGGAGAACCAGAAACTATCTGCTGACAGCG
ATAGTGGTGTTGACAATGGCGAGCGTGTTCTACAGCCCGTCTGCGGGCGTTCTGGGGGCCGTCGTCAT
TCTGACGGTATCCTTCCTGCCACGAGGAGATTTGGGGGGAAGAACGCTGGATGACAGTGCGCCAGTTG
GAGAGGCAGAGGGGATCTACCGGGTTTTTGAGCACATCGGACCATGGGCCTTCATGAAAGGTGTCGCT
ACTGTGACGGGGGGGAGCATCGTGTCCTCGCTTCATGTGACAGGGGATAGAGCAGTATGGATAGAGGA
CCGCAGGTATGAGCCCAGCGTGGTTCAACCTAGCGGCGACTTCATAGCTTGGGGGAGACCGCCGGTCA
TAAAACCTCTAAAGGAGGACGACGAGGTGGTGGCCCTGGCACTACACCCAATCACGGACACGGTGCTG
CCACTGAGATCGAGAACAGCCAGAATGCAGGGAAATGCTATCTACCGCATCAGCAAAACCTCACCAGG
AGTGAGCGGGTCACCGCTCTTTGTGGTTGACCTTAATGAGAATGGAGACCGCACCTTCGCACTAGCGG
GGACAATAGGGAGAAGTATACGTGCAGGACCCTACCATCAATATGAGATCCAATCACATCTGCCGTTG
CCTACCACACCATATGACACTATACTCAGAGCAGGCATAGTCCTCCAGCTATTTAGCCATCCGGGGGC
GGGAAAGACCCGTGCAATACCGGAGTACGTCAGGCAGCTTATGACCTGGTCCAACAAGGTCTATGTCG
CCGGCCCAACCCGGGTGGTTGCTAGAGAGATGCTGGAGGCGCTGGAGGGCACCCGGTGGGTGTGCGCC
ATGGTAAAGGGGCTTCCAAAACCCCACGCCTTGGCTAGAGTGGTCGTTACAACACACCAAACCTTACT
CCGCTACGCGCTCACCTCCGGGCTCTTGGCCTCGCGAGATGTGTCATATGTGCTGGATGAGACGCATG
TCGACTCAGCGCAGACCAAGGTTCTGCGAGCACTGGCACATCAGGCTGTGGGAAAGGACAAAAGCAAG
GCAGCTTGCATAGAGATGACTGCCACCGGGAGAGACTCCACCAGCGGAGAGATAAGAGTGGCGACGGA
TAGCAACTACCACATCGAAGAGCACGTCTATACCACACCTGTGGCCCAGGCAGTTAAACAGTATGCTG
ACACATACGGACCGAGGCGAATTGCGGTCTTCGTGCCAGGCCTGACGGGGAAGAGCGGAGCCTTACAG
GTAGCTAAACAGGTCAGGCAGCTCACCGACTACGCCACGGTGGTCCTGAGTAGGAAGACGTATGAAAA
GAACATCAAGCTGATCTTTAGGGGCTACCCTAGAGGGCTGTGCATAGTGACCACCTCCATCAGTGAAT
GTGGAGCCAACTATGACCTAGACGCAGTTTTTGACACATGCCAACAATACCACTACCTTGTTACGGCT
TCAGGAACCAAGGGAATGATAACCCCCTCCACCCAAGCCCAGACCTGCCAAAGGAGAGGCAGGGTTGG
AAGGAGAAAGGAGGGCCAGTACTACCGGCCAGCAAATTATGATCTGTTGCAAGCCCCTCCCCTGGATC
ACCCCGACTCGGTCACGCTGCTGGAGGCCAACATGTGCCTTAGGGCACTGGGGCTCCCAGAGGAGCCG
TGTGGAAGAGTTGTTGAGGAGGCGATGCTAAAAATGCAGCCCTCCAAGGACCAGGTGTATAGGTGGCT
GACCGAGGGAGACACGGAGACTCTAACAGAGACCATGGCCATGTACAATGTTGAAGGTGGAAGAAGAA
GCCGTGAGCAGGAGAGAACAGTTCGAAATAGGATGAAAACCTACTTCCATGACGTCAGATGGGAGAGA
GTGGAAGATCAAGAAGAAACACCACAACCAAGACCAGGAGAATACATCTGGGATGAAGAGGGCCCTGA
GATGATACAAGGAGCATCGTATGCTAGGGCCCCACCACCTCACAGAATACGAGTGAACGAAGCCACCT
ACCTCAGGGGCACAGTCATAGAGACCCTGAGAGAGGAGGCGGACAGAGAGCTGAGAGAGACGGTGGCT
GAGCAGCGGGAAGAATAGGCCGCGCTCACGTAGCCACACGGACGCGACTCGGACTGTCACACGGAACA
GAGGACAAGGATTACAAGTTTAAGACAAACACTGGCGCCCTAGCAAAGACTAGGTTGCGGCTAGCGCC
ACCGCAAACAAAAAAATAGAAAAAACGAAACACAAAAGTCTGTAGTTAAAGCG

>MT293_ON186514
AAACAGAAAAAACTCACAAGTGATTGTTGAGAACTTGACATGGCTACCCCAGTGGTAGCAGCCTTGAG
CTGCGTATTGGCCGCAGTGGACAAGGGATTGGCTCCCAATGTGGGGGCCAAGGGTCTCTTAGCTATGG
CGGCAGCATCTGCGTTGGCACCGGAATTGGTGCTGGCTGCGGCTTGGCTCTCCATACTGAAAGGAAAC
TCACCAGGTCGGGAAATGAACTACACACAAAGACACTTCATGGAGCTGCTGCCCACCATCGGGTGGCT
ATCTCTGGAATACGCGGCTCCACTGGATATCGTCCTAAACGAAGAAGACACACCACACCAGGAGGACA
GAAGACTATATGAGAAGGCACTGAACCTCTCCTACATAGGGAGAACCAGAAACTATTTGCTGACAGCG
ATAGTGGTGTTGACATTGGCGAGCGTGTTCTACAGCCCGTCTGCGGGCGTTCTGGGGGCCGTCGTCAT
TCTGACGGTATCCTTCCTGCCACGAGGAGATTTGGGGGGAAGAACGCTGGATGACAGTGCGCCAGTTG
GAGAGGCAGAGGGGATCTACCGGGTTTTTGAGCACATCGGACCATGGGCCTTCATGAAAGGTGTCGCT
ACTGTGACGGGGGGGAGCATCGTGTCCTCGCTTCATGTGACAGGGGATAGAGCAGTATGGATAGAGGA
CCGCAGGTATGAGCCCAGCGTGGTTCAACCTAGCGGCGACTTCATAGCTTGGGGGAGACCACCGGTCA

TAAAACCTCTAAAGGAGGACGACGAGGTGGTGGCCCTGGCACTACACCCAATCACGGACACGGTGCTG
CCACTGAGATCGAGAACAGCCAGAATGCAGGGGAATGCTATCTACCGCATCAGCAAAACCTCACCAGG
AGTGAGCGGGTCACCGCTCTTTGTGGTTGACCTTAATGAGAATGGAGACCGCACCTTCGCACTAGCGG
GGACAATAGGGAGAAGTATACGTGCAGGACCCTACCATCAATATGAGATCCAATCACATCTGCCGTTG
CCTACCACACCATATGACACTATACTCAGAGCAGGCATAGTCCTCCAGCTATTTAGCCATCCGGGGGC
GGGAAAGACCCGTGCAATACCGGAGTACGTCAGGCAGCTTATGACCTGGTCCAACAAGGTCTATGTCG
CCGGCCCAACCCGGGTGGTTGCTAGAGAGATGCTGGAGGCGCTGGAGGGCACCCGGTGGGTGTGCGCC
ATGGTAAAGGGGCTTCCAAAACCCCACGCCTTGGCCAGAGTGGTCGTTACAACACACCAAACCTTACT
CCGCTACGCGCTCACCTCCGGGCTCTTGGCCTCGCGAGATGTGTCATATGTGCTGGATGAGACGCATG
TCGACTCAGCGCAGACCAAGGTTCTGCGAGCACTGGTACATCAGGCTGTGGGAAAGGACAAAAGCAAG
GCAGCTTGCATAGAGATGACTGCCACCGGGAGAGACTCCACCAGCGGAGAGATAAGAGTGGCGACGGA
TAGCAACTACCACATCGAAGAGCACGTCTATACCACACCTGTGGCCCAGGCAGTTAAACAGTATGCTG
ACACATACGGACCGAGGCGAATTGCGGTCTTCGTGCCAGGCCTGACGGGGAAGAGCGGAGCCTTACAG
GTAGCTAAACAGGTCAGGCAGCTCACCGACTACGCCACGGTGGTCCTGAGTAGGAAGACGTATGAAAA
GAACATCAAGCTGATCTTTAGGGGCTACCCTAGAGGGCTGTGCATAGTGACCACCTCCATCAGTGAAT
GTGGAGCCAACTATGACCTAGACGCAGTTTTTGACACATGCCAACAATACCACTACCTTGTTACGGCT
TCAGGAACCAAGGGAATGATAACCCCCTCCACCCAAGCCCAGACCTGCCAAAGGAGAGGCAGGGTTGG
AAGGAGAAAGGAGGGCCAGTACTACCGGCCAGCAAATTATGATCTGTTGCAAGCCCCTCCCCTGGATC
ACCCCGACTCGGTCACGCTGCTGGAGGCCAACATGTGCCTTAGGGCACTGGGGCTCCCAGAGGAGCCG
TGTGGAAGAGTTGTTGAGGAGGCGATGCTAAAAATGCAGCCCTCCAAGGACCAGGTGTATAGGTGGCT
GACCGAGGGAGACACGGAGACTCTAACAGAGACCATGGCCATGTACAATGTTGAAGGTGGAAGAAGAA
GCCGTGAGCAGGAGAGAACAGTTCGAAATAGGATGAAAACCTACTTCCATGACGTCAGATGGAGAGA
GTGGAAGATCAAGAAGAAACACCACAACCAAGACCAGGAGAGTACATCTGGGATGAAGAGGGCCCTGA
GATGATACAAGGAGCATCGTATGCTAGGGCCCCACCACCTCACAGAATACGAGTGAACGAAGCCACCT
ACCTCAGGGGCACAGTCATAGAGACCCTGAGAGAGGAGGCGGACAGAGAGCTGAGAGAGACGGTGGCT
GAGCAGCGGGAAGAATAGGCCGCGCTCACGTAGCCACACGGACGCGACTCGGACTGTCACACGGAACA
GAGGACAAGGATCACAAGTTTAAGACAAACACTGGCGCCCTAGCAAAGACTAGGTTGCGGCTAGCGCC
ACCGCAAACAAAAAAATAGAAAAAACGAAACACAAAAGTCTGTAGTTAAAGCG

>MT299_ON186515
AAACAGAAAAAACTCACAAGTGATTGTTGAGAACTTGACATGGCTACCCCAGTGGTAGCAGCCTTGAG
CTGCGTATTGGCCGCAGTGGACAAGGGATTGGCTCCCAATGTGGGGGCCAAGGGTCTCTTAGCTATGG
CGGCAGCATCTGCGTTGGCACCGGAATTGGTGCTGGCTGCGGCTTGGCTCTCCATACTGAAAGGAAAC
TCACCAGGTCGGGAAATGAACTACACACAAAGACACTTCATGGAGCTGCTGCCCACCATCGGGTGGCT
ATCTCTGGAATACGCGGCTCCACTGGATATCGTCCTAAACGAAGAAGACACACCACACCAGGAGGACA
GAAGACTATATGAGAAGGCACTGAACCTCTCCTACATAGGGAGAACCAGAAACTATTTGCTGACAGCG
ATAGTGGTGTTGACATTGGCGAGCGTGTTCTACAGCCCGTCTGCGGGCGTTCTGGGGGCCGTCGTCAT
TCTGACGGTATCCTTCCTGCCACGAGGAGATTTGGGGGGAAGAACGCTGGATGACAGTGCGCCAGTTG
GAGAGGCAGAGGGGATCTACCGGGTTTTTGAGCACATCGGACCATGGGCCTTCATGAAAGGTGTCGCT
ACTGTGACGGGGGGGAGCATCGTGTCCTCGCTTCATGTGACAGGGGATAGAGCAGTATGGATAGAGGA
CCGCAGGTATGAGCCCAGCGTGGTTCAACCTAGCGGCGACTTCATAGCTTGGGGGAGACCACCGGTCA
TAAAACCTCTAAAGGAGGACGACGAGGTGGTGGCCCTGGCACTACACCCAATCACGGACACGGTGCTG
CCACTGAGATCGAGAACAGCCAGAATGCAGGGGAATGCTATCTACCGCATCAGCAAAACCTCACCAGG
AGTGAGCGGGTCACCGCTCTTTGTGGTTGACCTTAATGAGAATGGAGACCGCACCTTCGCACTAGCGG
GGACAATAGGGAGAAGTATACGTGCAGGACCCTACCATCAATATGAGATCCAATCACATCTGCCGTTG
CCTACCACACCATATGACACTATACTCAGAGCAGGCATAGTCCTCCAGCTATTTAGCCATCCGGGGGC
GGGAAAGACCCGTGCAATACCGGAGTACGTCAGGCAGCTTATGACCTGGTCCAACAAGGTCTATGTCG
CCGGCCCAACCCGGGTGGTTGCTAGAGAGATGCTGGAGGCGCTGGAGGGCACCCGGTGGGTGTGCGCC

ATGGTAAAGGGGCTTCCAAAACCCCACGCCTTGGCCAGAGTGGTCGTTACAACACACCAAACCTTACT
CCGCTACGCGCTCACCTCCGGGCTCTTGGCCTCGCGAGATGTGTCATATGTGCTGGATGAGACGCATG
TCGACTCAGCGCAGACCAAGGTTCTGCGAGCACTGGTACATCAGGCTGTGGGAAAGGACAAAAGCAAG
GCAGCTTGCATAGAGATGACTGCCACCGGGAGAGACTCCACCAGCGGAGAGATAAGAGTGGCGACGGA
TAGCAACTACCACATCGAAGAGCACGTCTATACCACACCTGTGGCCCAGGCAGTTAAACAGTATGCTG
ACACATACGGACCGAGGCGAATTGCGGTCTTCGTGCCAGGCCTGACGGGGAAGAGCGGAGCCTTACAG
GTAGCTAAACAGGTCAGGCAGCTCACCGACTACGCCACGGTGGTCCTGAGTAGGAAGACGTATGAAAA
GAACATCAAGCTGATCTTTAGGGGCTACCCTAGAGGGCTGTGCATAGTGACCACCTCCATCAGTGAAT
GTGGAGCCAACTATGACCTAGACGCAGTTTTTGACACATGCCAACAATACCACTACCTTGTTACGGCT
TCAGGAACCAAGGGAATGATAACCCCCTCCACCCAAGCCCAGACCTGCCAAAGGAGAGGCAGGGTTGG
AAGGAGAAAGGAGGGCCAGTACTACCGGCCAGCAAATTATGATCTGTTGCAAGCCCCTCCCCTGGATC
ACCCCGACTCGGTCACGCTGCTGGAGGCCAACATGTGCCTTAGGGCACTGGGGCTCCCAGAGGAGCCG
TGTGGAAGAGTTGTTGAGGAGGCGATGCTAAAAATGCAGCCCTCCAAGGACCAGGTGTATAGGTGGCT
GACCGAGGGAGACACGGAGACTCTAACAGAGACCATGGCCATGTACAATGTTGAAGGTGGAAGAAGAA
GCCGTGAGCAGGAGAGAACAGTTCGAAATAGGATGAAAACCTACTTCCATGACGTCAGATGGGAGAGA
GTGGAAGATCAAGAAGAAACACCACAACCAAGACCAGGAGAGTACATCTGGGATGAAGAGGGCCCTGA
GATGATACAAGGAGCATCGTATGCTAGGGCCCCACCACCTCACAGAATACGAGTGAACGAAGCCACCT
ACCTCAGGGGCACAGTCATAGAGACCCTGAGAGAGGAGGCGGACAGAGAGCTGAGAGAGACGGTGGCT
GAGCAGCGGGAAGAATAGGCCGCGCTCACGTAGCCACACGGACGCGACTCGGACTGTCACACGGAACA
GAGGACAAGGATCACAAGTTTAAGACAAACACTGGCGCCCTAGCAAAGACTAGGTTGCGGCTAGCGCC
ACCGCAAACAAAAAAATAGAAAAAACGAAACACAAAAGTCTGTAGTTAAAGCG

>MT308_ON186516
AAACAGAAAAAACTCACAAGTGATTGTTGAGAACTTGACATGGCTACCCCAGTGGTAGCAGCCTTGAG
CTGCGTATTGGCCGCAGTGGACAAGGGATTGGCTCCCAATGTGGGGGCCAAGGGTCTCTTAGCTATGG
CGGCAGCATCTGCGTTGGCACCGGAATTGGTGCTGGCTGCAGCTTGGCTCTCCATACTGAAAGGAAAC
TCACCAGGTCGGGAAATGAACTACACACAAAGACACTTCATGGAGCTGCTGCCCACCATCGGATGGCT
ATCTCTGGAATACGCGGCTCCACTGGATATCGTCCTAAACGAAGAAGACACACCACACCAGGAGGACA
GAAGACTATATGAGAAGGCACTGAGCCTCTCCTACATAGGGAGAACCAGAAACTATTTGCTGACAGCG
ATAGTGGTGTTGACATTGGCGAGCGTGTTCTACAGCCCGTCTGCGGGCGTTCTGGGGGCCGTTGTCAT
TCTGACGGTATCCTTCCTGCCACGAGGAGATCTGGGGGGAAGAACGCTGGATGACAGTGCGCCAGTTG
GAGAGGCAGAGGGGATCTACCGGGTTTTTGAGCACATCGGACCATGGGCCTTCATGAAAGGTGTCGCT
ACTGTGACGGGGGGGAGCATCGTGTCCTCGCTTCATGTGACAGGGGATAGAGCAGTATGGATAGAGGA
CCGCAGGTATGAGCCCAGCGTGGTTCAGCCTAGCGGCGACTTCATAGCTTGGGGGAGACCACCGGTCA
TAAAACCTCTAAAGGAGGACGACGAGGTGGTGGCCCTGGCACTACACCCAATCACGGACACGGTGCTG
CCACTGAGATCGAGAACAGCCAGAATGCAGGGAAATGCTATCTACCGCATCAGCAAAACCTCACCAGG
AGTGAGCGGGTCACCGCTCTTTGTGGTTGACCTTAATGAGAATGGAGACCGCACCTTCGCACTAGCGG
GGACAATAGGGAGAAGTATACGTGCAGGACCCTACCATCAATATGAGATCCAATCACATTTGCCGTTG
CCTACCACACCATATGACACTATACTCAGAGCAGGCATAGTCCTCCAGCTATTTAGCCATCCGGGGGC
GGGAAAGACCCGTGCAATACCGGAGTACGTCAGGCAGCTTATGACCTGGTCCAACAAGGTCTATGTCG
CCGGCCCAACCCGGGTGGTTGCTAGAGAGATGCTGGAGGCGCTGGAGGGCACCCGGTGGGTGTGCGCC
ATGGTAAAGGGGCTTCCAAAACCCCACGCCTTGGCCAGAGTGGTTGTTACAACACACCAAACCTTACT
CCGCTACGCGCTCACCTCCGGGCTCTTGGCCTCGCGAGATGTGTCATATGTGCTGGATGAGACGCATG
TCGACTCAGCGCAGACCAAGGTTCTGCGAGCACTGGTACATCAGGCTGTGGGAAAGGACAAAAGCAAG
GCAGCTTGCATAGAGATGACTGCCACCGGGAGAGACTCCACCAGCGGAGAGATAAGAGTGGCGACGGA
TAGCAACTACCACATCGAAGAGCACGTCTATACCACACCTGTGGCCCAGGCAGTTAAACAGTATGCTG
ACACATACGGACCGAGGCGAATTGCGGTCTTCGTGCCAGGCCTGACGGGGAAGAGCGGAGCCTTACAG
GTAGCTAAACAGGTCAGGCAGCTCACCGACTACGCCACGGTGGTCCTGAGTAGGAAGACGTATGAAAA

GAACATCAAGCTGATCTTTAGGGGCTACCCTAGAGGGCTGTGCATAGTGACCACCTCCATCAGTGAAT
GTGGAGCCAACTATGACCTAGACGCAGTTTTTGACACATGCCAACAATACCACTACCTTGTTACGGCT
TCAGGAACTAAGGGAATGATAACCCCCTCCACCCAAGCCCAGACCTGCCAAAGGAGAGGCAGGGTTGG
AAGGAGAAAGGAGGGCCAGTACTACCGGCCAGCAAATTATGATCTGCTGCAAGCCCCTCCCCTGGATC
ACCCCGACTCGGTCACTCTGCTGGAGGCCAACATGTGCCTTAGGGCACTGGGGCTCCCGGAGGAGCCG
TGTGGAAGAGTTGTTGAGGAGGCGATGCTAAAAATGCAGCCCTCCAAGGATCAGGTGTATAGGTGGCT
GACCGAGGGAGACACGGAGACTCTAACAGAGACCATGGCCATGTACAACGTTGAAGGTGGAAGAAGAA
GCCGTGAGCAGGAGAGAACAGTTCGAAATAGGATGAAAACCTACTTCCATGACGTCAGATGGGAGAGA
GTGGAAGATCAAGAAGAAACACCACAACCAAGACCAGGAGAATACATCTGGGATGAAGAGGGCCCTGA
GATGATACAAGGAGCATCGTATGCTAGGGCCCCACCACCTCACAGAATACGAGTGAACGAAGCCACCT
ACCTCAGGGGCACAGTCATAGAGACCCTGAGAGAGGAGGCGGACAGAGAGCTGAGAGAGACGGTGGCT
GAGCAGCGGGAAGAATAGGCCGCGCTCACGTAGCCACACGGACGCGACTCGGACTGTCACACGGAACA
GAGGACAAGGATCACAAGTTCAAGACAAACACTGGCGCCCTAGCAAAGACTAGGTTGCGGCTCGCGCC
ACCGCAAACAAAAAAATAGAAAAAACGAAACACAAAAGTCTGTAGTTAAAGCG

>MT290_ON186517
AAACAGAAAAAACTCACAAGTGATTGTTGAGAACTTGACATGGCTACCCCAGTGGTAGCAGCCTTGAG
CTGCGTATTGGCCGCAGTGGACAAGGGATTGGCTCCCAATGTGGGGGCCAAGGGTCTCTTAGCTATGG
CGGCAGCATCTGCGTTGGCACCGGAATTGGTGCTGGCTGCAGCTTGGCTCTCCATACTGAAAGGAAAC
TCACCAGGTCGGGAAATGAACTACACACAAAGACACTTCATGGAGCTGCTGCCCACCATCGGATGGCT
ATCTCTGGAATACGCGGCTCCACTGGATATCGTCCTAAACGAAGAAGACACACCACACCAGGAGGACA
GAAGACTATATGAGAAGGCACTGAGCCTCTCCTACATAGGGAGAACCAGAAACTATTTGCTGACAGCG
ATAGTGGTGTTGACATTGGCGAGCGTGTTCTACAGCCCGTCTGCGGGCGTTCTGGGGGCCGTTGTCAT
TCTGACGGTATCCTTCCTGCCACGAGGAGATCTGGGGGGAAGAACGCTGGATGACAGTGCGCCAGTTG
GAGAGGCAGAGGGGATCTACCGGGTTTTTGAGCACATCGGACCATGGGCCTTCATGAAAGGTGTCGCT
ACTGTGACGGGGGGGAGCATCGTGTCCTCGCTTCATGTGACAGGGGATAGAGCAGTATGGATAGAGGA
CCGCAGGTATGAGCCCAGCGTGGTTCAGCCTAGCGGCGACTTCATAGCTTGGGGGAGACCACCGGTCA
TAAAACCTCTAAAGGAGGACGACGAGGTGGTGGCCCTGGCACTACACCCAATCACGGACACGGTGCTG
CCACTGAGATCGAGAACAGCCAGAATGCAGGGAAATGCTATCTACCGCATCAGCAAAACCTCACCAGG
AGTGAGCGGGTCACCGCTCTTTGTGGTTGACCTTAATGAGAATGGAGACCGCACCTTCGCACTAGCGG
GGACAATAGGGAGAAGTATACGTGCAGGACCCTACCATCAATATGAGATCCAATCACATTTGCCGTTG
CCTACCACACCATATGACACTATACTCAGAGCAGGCATAGTCCTCCAGCTATTTAGCCATCCGGGGGC
GGGAAAGACCCGTGCAATACCGGAGTACGTCAGGCAGCTTATGACCTGGTCCAACAAGGTCTATGTCG
CCGGCCCAACCCGGGTGGTTGCTAGAGAGATGCTGGAGGCGCTGGAGGGCACCCGGTGGGTGTGCGCC
ATGGTAAAGGGGCTTCCAAAACCCCACGCCTTGGCCAGAGTGGTTGTTACAACACACCAAACCTTACT
CCGCTACGCGCTCACCTCCGGGCTCTTGGCCTCGCGAGATGTGTCATATGTGCTGGATGAGACGCATG
TCGACTCAGCGCAGACCAAGGTTCTGCGAGCACTGGTACATCAGGCTGTGGGAAAGGACAAAAGCAAG
GCAGCTTGCATAGAGATGACTGCCACCGGGAGAGACTCCACCAGCGGAGAGATAAGAGTGGCGACGGA
TAGCAACTACCACATCGAAGAGCACGTCTATACCACACCTGTGGCCCAGGCAGTTAAACAGTATGCTG
ACACATACGGACCGAGGCGAATTGCGGTCTTCGTGCCAGGCCTGACGGGGAAGAGCGGAGCCTTACAG
GTAGCTAAACAGGTCAGGCAGCTCACCGACTACGCCACGGTGGTCCTGAGTAGGAAGACGTATGAAAA
GAACATCAAGCTGATCTTTAGGGGCTACCCTAGAGGGCTGTGCATAGTGACCACCTCCATCAGTGAAT
GTGGAGCCAACTATGACCTAGACGCAGTTTTTGACACATGCCAACAATACCACTACCTTGTTACGGCT
TCAGGAACTAAGGGAATGATAACCCCCTCCACCCAAGCCCAGACCTGCCAAAGGAGAGGCAGGGTTGG
AAGGAGAAAGGAGGGCCAGTACTACCGGCCAGCAAATTATGATCTGCTGCAAGCCCCTCCCCTGGATC
ACCCCGACTCGGTCACTCTGCTGGAGGCCAACATGTGCCTTAGGGCACTGGGGCTCCCGGAGGAGCCG
TGTGGAAGAGTTGTTGAGGAGGCGATGCTAAAAATGCAGCCCTCCAAGGATCAGGTGTATAGGTGGCT
GACCGAGGGAGACACGGAGACTCTAACAGAGACCATGGCCATGTACAACGTTGAAGGTGGAAGAAGAA

GCCGTGAGCAGGAGAGAACAGTTCGAAATAGGATGAAAACCTACTTCCATGACGTCAGATGGGAGAGA
GTGGAAGATCAAGAAGAAACACCACAACCAAGACCAGGAGAATACATCTGGGATGAAGAGGGCCCTGA
GATGATACAAGGAGCATCGTATGCTAGGGCCCCACCACCTCACAGAATACGAGTGAACGAAGCCACCT
ACCTCAGGGGCACAGTCATAGAGACCCTGAGAGAGGAGGCGGACAGAGAGCTGAGAGAGACGGTGGCT
GAGCAGCGGGAAGAATAGGCCGCGCTCACGTAGCCACACGGACGCGACTCGGACTGTCACACGGAACA
GAGGACAAGGATCACAAGTTCAAGACAAACACTGGCGCCCTAGCAAAGACTAGGTTGCGGCTCGCGCC
ACCGCAAACAAAAAAATAGAAAAAACGAAACACAAAAGTCTGTAGTTAAAGCG

>MT304_ON186518
AAACAGAAAAAACTCACAAGTGATTGTTGAGAACTTGACATGGCTACCCCAGTGGTAGCAGCCTTGAG
CTGCGTATTGGCCGCAGTGGACAAGGGATTGGCTCCCAATGTGGGGGCCAAGGGTCTCTTAGCTATGG
CGGCAGCATCTGCGTTGGCACCGGAATTGGTGCTGGCTGCAGCTTGGCTCTCCATACTGAAAGGAAAC
TCACCAGGTCGGGAAATGAACTACACACAAAGACACTTCATGGAGCTGCTGCCCACCATCGGATGGCT
ATCTCTGGAATACGCGGCTCCACTGGATATCGTCCTAAACGAAGAAGACACACCACACCAGGAGGACA
GAAGACTATATGAGAAGGCACTGAGCCTCTCCTACATAGGGAGAACCAGAAACTATTTGCTGACAGCG
ATAGTGGTGTTGACATTGGCGAGCGTGTTCTACAGCCCGTCTGCGGGCGTTCTGGGGGCCGTTGTCAT
TCTGACGGTATCCTTCCTGCCACGAGGAGATCTGGGGGGAAGAACGCTGGATGACAGTGCGCCAGTTG
GAGAGGCAGAGGGGATCTACCGGGTTTTTGAGCACATCGGACCATGGGCCTTCATGAAAGGTGTCGCT
ACTGTGACGGGGGGGAGCATCGTGTCCTCGCTTCATGTGACAGGGGATAGAGCAGTATGGATAGAGGA
CCGCAGGTATGAGCCCAGCGTGGTTCAGCCTAGCGGCGACTTCATAGCTTGGGGGAGACCACCGGTCA
TAAAACCTCTAAAGGAGGACGACGAGGTGGTGGCCCTGGCACTACACCCAATCACGGACACGGTGCTG
CCACTGAGATCGAGAACAGCCAGAATGCAGGGAAATGCTATCTACCGCATCAGCAAAACCTCACCAGG
AGTGAGCGGGTCACCGCTCTTTGTGGTTGACCTTAATGAGAATGGAGACCGCACCTTCGCACTAGCGG
GGACAATAGGGAGAAGTATACGTGCAGGACCCTACCATCAATATGAGATCCAATCACATTTGCCGTTG
CCTACCACACCATATGACACTATACTCAGAGCAGGCATAGTCCTCCAGCTATTTAGCCATCCGGGGGC
GGGAAAGACCCGTGCAATACCGGAGTACGTCAGGCAGCTTATGACCTGGTCCAACAAGGTCTATGTCG
CCGGCCCAACCCGGGTGGTTGCTAGAGAGATGCTGGAGGCGCTGGAGGGCACCCGGTGGGTGTGCGCC
ATGGTAAAGGGGCTTCCAAAACCCCACGCCTTGGCCAGAGTGGTTGTTACAACACACCAAACCTTACT
CCGCTACGCGCTCACCTCCGGGCTCTTGGCCTCGCGAGATGTGTCATATGTGCTGGATGAGACGCATG
TCGACTCAGCGCAGACCAAGGTTCTGCGAGCACTGGTACATCAGGCTGTGGGAAAGGACAAAAGCAAG
GCAGCTTGCATAGAGATGACTGCCACCGGGAGAGACTCCACCAGCGGAGAGATAAGAGTGGCGACGGA
TAGCAACTACCACATCGAAGAGCACGTCTATACCACACCTGTGGCCCAGGCAGTTAAACAGTATGCTG
ACACATACGGACCGAGGCGAATTGCGGTCTTCGTGCCAGGCCTGACGGGGAAGAGCGGAGCCTTACAG
GTAGCTAAACAGGTCAGGCAGCTCACCGACTACGCCACGGTGGTCCTGAGTAGGAAGACGTATGAAAA
GAACATCAAGCTGATCTTTAGGGGCTACCCTAGAGGGCTGTGCATAGTGACCACCTCCATCAGTGAAT
GTGGAGCCAACTATGACCTAGACGCAGTTTTTGACACATGCCAACAATACCACTACCTTGTTACGGCT
TCAGGAACTAAGGGAATGATAACCCCCTCCACCCAAGCCCAGACCTGCCAAAGGAGAGGCAGGGTTGG
AAGGAGAAAGGAGGGCCAGTACTACCGGCCAGCAAATTATGATCTGCTGCAAGCCCCTCCCCTGGATC
ACCCCGACTCGGTCACTCTGCTGGAGGCCAACATGTGCCTTAGGGCACTGGGGCTCCCGGAGGAGCCG
TGTGGAAGAGTTGTTGAGGAGGCGATGCTAAAAATGCAGCCCTCCAAGGATCAGGTGTATAGGTGGCT
GACCGAGGGAGACACGGAGACTCTAACAGAGACCATGGCCATGTACAACGTTGAAGGTGGAAGAAGAA
GCCGTGAGCAGGAGAGAACAGTTCGAAATAGGATGAAAACCTACTTCCATGACGTCAGATGGGAGAGA
GTGGAAGATCAAGAAGAAACACCACAACCAAGACCAGGAGAATACATCTGGGATGAAGAGGGCCCTGA
GATGATACAAGGAGCATCGTATGCTAGGGCCCCACCACCTCACAGAATACGAGTGAACGAAGCCACCT
ACCTCAGGGGCACAGTCATAGAGACCCTGAGAGAGGAGGCGGACAGAGAGCTGAGAGAGACGGTGGCT
GAGCAGCGGGAAGAATAGGCCGCGCTCACGTAGCCACACGGACGCGACTCGGACTGTCACACGGAACA
GAGGACAAGGATCACAAGTTCAAGACAAACACTGGCGCCCTAGCAAAGACTAGGTTGCGGCTCGCGCC
ACCGCAAACAAAAAAATAGAAAAAACGAAACACAAAAGTCTGTAGTTAAAGCG

>MT297_ON186519
AAACAGAAAAAACTCACAAGTGATTGTTGAGAACTTGACATGGCTACCCCAGTGGTAGCAGCCTTGAG
CTGCGTATTGGCCGCAGTGGACAAGGGATTGGCTCCCAATGTGGGGGCCAAGGGTCTCTTAGCTATGG
CGGCAGCATCTGCGTTGGCACCGGAATTGGTGCTGGCTGCAGCTTGGCTCTCCATACTGAAAGGAAAC
TCACCAGGTCGGGAAATGAACTACACACAAAGACACTTCATGGAGCTGCTGCCCACCATCGGATGGCT
ATCTCTGGAATACGCGGCTCCACTGGATATCGTCCTAAACGAAGAAGACACACCACACCAGGAGGACA
GAAGACTATATGAGAAGGCACTGAGCCTCTCCTACATAGGGAGAACCAGAAACTATTTGCTGACAGCG
ATAGTGGTGTTGACATTGGCGAGCGTGTTCTACAGCCCGTCTGCGGGCGTTCTGGGGGCCGTTGTCAT
TCTGACGGTATCCTTCCTGCCACGAGGAGATCTGGGGGGAAGAACGCTGGATGACAGTGCGCCAGTTG
GAGAGGCAGAGGGGATCTACCGGGTTTTTGAGCACATCGGACCATGGGCCTTCATGAAAGGTGTCGCT
ACTGTGACGGGGGGGAGCATCGTGTCCTCGCTTCATGTGACAGGGGATAGAGCAGTATGGATAGAGGA
CCGCAGGTATGAGCCCAGCGTGGTTCAGCCTAGCGGCGACTTCATAGCTTGGGGGAGACCACCGGTCA
TAAAACCTCTAAAGGAGGACGACGAGGTGGTGGCCCTGGCACTACACCCAATCACGGACACGGTGCTG
CCACTGAGATCGAGAACAGCCAGAATGCAGGGAAATGCTATCTACCGCATCAGCAAAACCTCACCAGG
AGTGAGCGGGTCACCGCTCTTTGTGGTTGACCTTAATGAGAATGGAGACCGCACCTTCGCACTAGCGG
GGACAATAGGGAGAAGTATACGTGCAGGACCCTACCATCAATATGAGATCCAATCACATTTGCCGTTG
CCTACCACACCATATGACACTATACTCAGAGCAGGCATAGTCCTCCAGCTATTTAGCCATCCGGGGGC
GGGAAAGACCCGTGCAATACCGGAGTACGTCAGGCAGCTTATGACCTGGTCCAACAAGGTCTATGTCG
CCGGCCCAACCCGGGTGGTTGCTAGAGAGATGCTGGAGGCGCTGGAGGGCACCCGGTGGGTGTGCGCC
ATGGTAAAGGGGCTTCCAAAACCCCACGCCTTGGCCAGAGTGGTTGTTACAACACACCAAACCTTACT
CCGCTACGCGCTCACCTCCGGGCTCTTGGCCTCGCGAGATGTGTCATATGTGCTGGATGAGACGCATG
TCGACTCAGCGCAGACCAAGGTTCTGCGAGCACTGGTACATCAGGCTGTGGGAAAGGACAAAAGCAAG
GCAGCTTGCATAGAGATGACTGCCACCGGGAGAGACTCCACCAGCGGAGAGATAAGAGTGGCGACGGA
TAGCAACTACCACATCGAAGAGCACGTCTATACCACACCTGTGGCCCAGGCAGTTAAACAGTATGCTG
ACACATACGGACCGAGGCGAATTGCGGTCTTCGTGCCAGGCCTGACGGGGAAGAGCGGAGCCTTACAG
GTAGCTAAACAGGTCAGGCAGCTCACCGACTACGCCACGGTGGTCCTGAGTAGGAAGACGTATGAAAA
GAACATCAAGCTGATCTTTAGGGGCTACCCTAGAGGGCTGTGCATAGTGACCACCTCCATCAGTGAAT
GTGGAGCCAACTATGACCTAGACGCAGTTTTTGACACATGCCAACAATACCACTACCTTGTTACGGCT
TCAGGAACTAAGGGAATGATAACCCCCTCCACCCAAGCCCAGACCTGCCAAAGGAGAGGCAGGGTTGG
AAGGAGAAAGGAGGGCCAGTACTACCGGCCAGCAAATTATGATCTGCTGCAAGCCCCTCCCCTGGATC
ACCCCGACTCGGTCACTCTGCTGGAGGCCAACATGTGCCTTAGGGCACTGGGGCTCCCGGAGGAGCCG
TGTGGAAGAGTTGTTGAGGAGGCGATGCTAAAAATGCAGCCCTCCAAGGATCAGGTGTATAGGTGGCT
GACCGAGGGAGACACGGAGACTCTAACAGAGACCATGGCCATGTACAACGTTGAAGGTGGAAGAAGAA
GCCGTGAGCAGGAGAGAACAGTTCGAAATAGGATGAAAACCTACTTCCATGACGTCAGATGGGAGAGA
GTGGAAGATCAAGAAGAAACACCACAACCAAGACCAGGAGAATACATCTGGGATGAAGAGGGCCCTGA
GATGATACAAGGAGCATCGTATGCTAGGGCCCCACCACCTCACAGAATGCGAGTGAACGAAGCCACCT
ACCTCAGGGGCACAGTCATAGAGACCCTGAGAGAGGAGGCGGACAGAGAGCTGAGAGAGACGGTGGCT
GAGCAGCGGGAAGAATAGGCCGCGCTCACGTAGCCACACGGACGCGACTCGGACTGTCACACGGAACA
GAGGACAAGGATCACAAGTTCAAGACAAACACTGGCGCCCTAGCAAAGACTAGGTTGCGGCTCGCGCC
ACCGCAAACAAAAAAATAGAAAAAACGAAACACAAAAGTCTGTAGTTAAAGCG

>MT328_ON186520
AGTTGCAAGTGCCATAGCTTGGAATCCCGCCGCAGACTCAAACGAACGAAACACTCCAAAAAGCAAAA
CCGGCTTCGCAGCTACTGCTAGAAACAGACGGATTTGTGGGAGTACCAACAACAACAATGCCAAACAT
AAAAACTGCCTTGCTCTTGGTCACCCTGATGGGGTTGGCCTTAGGAGGCAATGACAAGCCAGACTGGG
AAAAGGTGAAGCTGCTCGGACAGCAACTGCAGTCGGGCATTGCTACAAAGAACGCCGTAAAAGTGAAG
TACTACGCTGAACTAATCATCTCTGAGATGCCTACTGACGAAACAACATATTATGCTGGCCTGAGAAC

AGGACAAGAAGTGGCACTCACGGTGGGCTCGGCGAAGTCCATCGTCGGAGAGACTCTGAACTTAAGAA
TCAACCAATGGACTAGGGCAGTCGGGGAGTATCTCATGATCGACCGGCATGTCCCAGGACTGACCCTC
CTGATCACGGGAGAGGATGTCTTGAGTGATCTCATGGAATTCGGGTGGAGCTACAGGCTTAACCCTAG
CGGACACTACCCTGTATACGCAGCCACCAAGACCCTACCCGGGAAGGGGGGCAGGTACACTGGTGGAA
AGACCTACACCGACAGGTATGCCGCGTTCTATGACGGGCACGTAAAAGACCACATGGTAGCAATACAC
CCTCACCAGGCTGACATCGAGAGAATGACTAAATACGAACCAATCAACCTAGTCTACGCAGATCTAAC
ACCGAGGAGCATTGGTGGAAGACTCGAGCACGTTCTCGTGACGAAGGGCCACTACACAATTCAGGCAG
GGAAAATCACCCACGTGTGGGGAGATAAAAAAGACTGTGAAACATGTGATCCGAACTACCAGGTTTTT
CAGTAGAGGGGGGACACGCCCAGCCGGTGCCCCCCCGTTCCCGGAAGACAACACCCACGGGAAGGGAA
GGTTGGATTCCTGACTGGATGATCATCGCAGGAGATTACTTCAAAAGGCTACAGGGGAAGGCTAACAT
CATGGGAGAGGGCTTTGACATCATCTTGGATGAAGCTAGGGACCTGTTTCCGTCAAGCAAAGAGTTTG
CCCTGCCAGGCTTGATACGACAGTACCTCGAGAGACGGGACGTACCACTGGATTCGGCCTTCTTTCTG
GATGTTTTCCTACCGCTGGTCTTGATGGCCCTTGCCATCACGACCAACCGATGGACTCGAATGGCCCT
GGCCGTTGGGGTGTACCTTACCGGGTACTACTACATAGCCATCATGCTGGCCGCCACCTCGGTGGTGT
CGCTAGCTTTCAAGGCATTCGCACCAAGGAAATGTGAGCGCGGGGACGTTGTGATCGAGAATGGGAGA
CTCAGTGTGCTGGGTATCACCGCGGTAGCCGTGGCTGCCACGGTGGGCATCCACTACTGGCAACCAAG
TCCTTCGCTCATATTTGCGGTGGCCTCATTGGCCGGGTTCGCCGCAATCCTCATGGCTTTACCGACCA
TCCAATACCATGGCGCCACAGACGTCGCTAAGATGATGATGGCGGTCCTCCTATTCGCAGGGGTGATC
TACATCGCGTCCTCTTTTGATGTCGACAAGGACCAGCTATACTTGTTTGTAAAGACGGGCACCCCGAC
CTACCACATCCCACGTGGAGAGAGAGAAAACGCAGTAAAGCTGGATCAGGTCTACAACCTGGCCCGAT
ACTACGCAAGGTATCCAGCTGATCTGCGTGCCAGATTCGTGACAGAAGAGGCGATATGGGAGTGGGCC
CCCGGCACCACATGGACGGTGGCTGAGATGGACTACATGGCAACAATTCTCCACGCGATCGTCTTCAG
CCTCGCGGTTTACACCATGTTGACGGATTCAGGGGAAACGGACAGGGTCCTTGGGGACATAAAGATGC
GTATCCTAGAAAAAATCAAGACCACAGAGCTCTTTGGGGATGTGACGATGGCAAAGATAACTCTCGCT
ATCAGCAGGGTTGAGTGGCTTGTCGTCATAGCGGGAAATCTATTGCACACCTACTTTGCCCTAGGGCT
TCCGGGCGTTGCGGTCGAGATCCTCCTGGCAGCCGGACTGATGGTACCCACTTACCACCTGTGGAGCA
GGACCTACCGAGTCATGTCATACCTGAGAGGGACTAATGGCTACAGACAGCCTGTTGAGGGCCTGCTC
CCAGCCCCTACGATAAGGAACACCCAAACGCAGTACACAGCAGGATTGGCAACAATAGTGGGAATCGC
GGCTGTGCTCGTAAACCTGTACTACTACCTGATAGGAAGCAACCCCTATTATGTAGCCCACAGCGTGC
TAGTCTTCGCAGGCACGTTTGTGATGGTCCAGGACAACGACCAGTTGAACGGCTACCCACTCTTAATG
TTGATGGCATACACATTTAACTCCCCAAGTCTGGCACTCATAGGTAGCATCTACAAAAAGTGCCTAGG
TAGAGTCCTGTGGAGCAGGACGACTTAGGGGGTTTCGTTCATACCGGAAAAGAC

>MT308_ON186521
AGTTGCAAGTGCCATAGCTTGGAATCCCGCCGCAGACTCAAACGAACGAAACACTCCAAAAAGCAAAA
CCGGCTTCGCAGCTACTGCTAGAAACAGACGGATTTGTGGGAGTACCAACAACAACAATGCCAAACAC
AAAAACTGCCTTGCTCTTGGTCACCCTGATGGGGTTGGCCTTAGGAGGCAATGACAAGCCAGACTGGG
AAAAGGTGAAGCTGCTCGGACAGCAACTGCAGTCGGGCATTGCTACAAAGAACGCCGTAAAAGTGAAG
TACTACGCTGAACTAATCATCTCTGAGATGCCTACTGACGAAACAACATATTATGCTGGCCTGAGAAC
AGGACAAGAAGTGGCACTCACGGTGGGCTCGGCGAAGTCCATCGTCGGAGAGACTCTGAACTTAAGAA
TCAACCAATGGACTAGGGCAGTCGGGGAGTATCTCATGATCGACCGGCATGTCCCAGGACTGACCCTC
CTGATCACGGGAGAGGATGTCTTGAGTGATCTCATGGAATTCGGGTGGAGCTACAGGCTTAACCCTAG
CGGACACTACCCTGTATACGCAGCCACCAAGACCCTACCCGGGAAGGGGGGCAGGTACACTGGTGGAA
AGACCTACACCGACAGGTATGCCGCGTTCTATGACGGACACGTAAAAGACCACATGGTAGCAATACAC
CCTCACCAGGCTGACATCGAGAGAATGACTAAATACGAACCAATCAACCTAGTCTACGCAGATCTAAC
ACCGAGGAGCATTGGTGGAAGACTCGAGCACGTTCTCGTGACGAAGGGCCACTACACAATTCAGGCAG
GGAAAATCACCCACGTGTGGGGAGATAAGAAAGACTGTGAAACATGTGATCCGAACTACCAGGTTTTT
CAGTAGAGGGGGGACACGCCCAACCGGTGCCCCCCCGTTCCCGGAAGACAACACCCACGGGAAGGGAA

GGTTGGATTCCTGACTGGATGATCATCGCAGGAGATTACTTCAAAAGGCTACAGGGGAAGGCTAACAT
CATGGGAGAGGGCTTTGACATCATCTTGGATGAAGCTAGGGACCTGTTTCCGTCAAGCAAAGAGTTTG
CCCTACCAGGCTTGATACGACAGTACCTCGAGAGACGGGACGTACCACTGGATTCGGCCTTCTTTCTG
GATGTTTTCCTACCGCTGGTCTTGATGGCCCTTGCCATCACGACCAACCGATGGACTCGAATGGCCCT
GGCCGTTGGGGTGTACCTTACCGGGTACTACTACATAGCCATCATGCTGGCCGCCACCTCGGTGGTGT
CGCTAGCTTTCAAGGCATTCGCACCAAGGAAATGTGAGCGCGGGGACGTTGTGATTGAGAATGGGAGA
CTCAGTGTGCTGGGTATCACCGCGGTAGCCGTGGCTGCCACGGTGGGCATCCACTACTGGCAACCAAG
TCCTTCGCTCATATTTGCGGTGGCCTCATTGGCCGGGTTCGCCGCAATCCTCATGGCTTTACCGACTA
TCCAATACCATGGCGCCACAGACGTCGCTAAGATGATGATGGCGGTCCTCCTATTCGCAGGGGTGATC
TACATCGCGTCCTCTTTTGATGTCGACAAGGACCAGCTGTACTTGTTTGTAAAGACGGGCACCCCGAC
CTACCACATCCCACGTGGAGAGAGAGAAAACGCAGTAAAGCTGGATCAGGTCTACAACCTGGCCCGAT
ACTACGCAAGGTATCCAGCAGATCTGCGTGCCAGATTCGTGACAGAGGAGGCGATATGGGAGTGGGCC
CCCGGCGCCACATGGACGGTGGCTGAGATGGACTACATGGCAACAATTCTCCATGCGATCGTCTTCAG
CCTCGCGGTTTACACCATGTTGACGGATTCAGGGGAAACGGACAGGGTCCTTGGGGACATAAAGATGC
GTATCCTAGAAAAAATCAAGACCACAGAGCTCTTTGGGGATGTGACGATGGCAAAGATAACTCTCGCT
ATCAGCAGGGTTGAGTGGCTTGTCGTCATAGCGGGAAATCTATTGCACACCTACTTTGCCCTAGGGCT
TCCGGGCGTTGCGGTCGAGATCCTCCTGGCAGCCGGACTGATGGTACCCACTTACCACCTGTGGAGCA
GGACCTACCGAGTCATGTCATACCTGAGAGGGACTAATGGCTACAGACAGCCTGTTGAGGGCTTGCTC
CCAGCCCCTACGATAAGGAACACCCAAACGCAGTACACAGCAGGATTGGCAACAATAGTGGGAATCGC
GGCTGTGCTCGTAAACCTGTACTACTACCTGATAGGAAGCAACCCCTATTATGTAGCCCACAGCGTGC
TAGTCTTCGCAGGCACGTTTGTGATGGTCCAGGACAACGACCAGTTGAACGGCTACCCACTCTTAATG
TTGATGGCATACACATTTAACTCCCCAAGTCTGGCACTCATAGGTAGCATCTACAAAAAGTGCCTAGG
TAGAGTCCTGTGGAGCAGGACGACTTAGGGGGTTTCGTTCATACCGGAAAAGAC

>MT304_ON186522
AGTTGCAAGTGCCATAGCTTGGAATCCCGCCGCAGACTCAAACGAACGAAACACTCCAAAAAGCAAAA
CCGGCTTCGCAGCTACTGCTAGAAACAGACGGATTTGTGGGAGTACCAACAACAACAATGCCAAACAC
AAAAACTGCCTTGCTCTTGGTCACCCTGATGGGGTTGGCCTTAGGAGGCAATGACAAGCCAGACTGGG
AAAAGGTGAAGCTGCTCGGACAGCAACTGCAGTCGGGCATTGCTACAAAGAACGCCGTAAAAGTGAAG
TACTACGCTGAACTAATCATCTCTGAGATGCCTACTGACGAAACAACATATTATGCTGGCCTGAGAAC
AGGACAAGAAGTGGCACTCACGGTGGGCTCGGCGAAGTCCATCGTCGGAGAGACTCTGAACTTAAGAA
TCAACCAATGGACTAGGGCAGTCGGGGAGTATCTCATGATCGACCGGCATGTCCCAGGACTGACCCTC
CTGATCACGGGAGAGGATGTCTTGAGTGATCTCATGGAATTCGGGTGGAGCTACAGGCTTAACCCTAG
CGGACACTACCCTGTATACGCAGCCACCAAGACCCTACCCGGGAAGGGGGGCAGGTACACTGGTGGAA
AGACCTACACCGACAGGTATGCCGCGTTCTATGACGGACACGTAAAAGACCACATGGTAGCAATACAC
CCTCACCAGGCTGACATCGAGAGAATGACTAAATACGAACCAATCAACCTAGTCTACGCAGATCTAAC
ACCGAGGAGCATTGGTGGAAGACTCGAGCACGTTCTCGTGACGAAGGGCCACTACACAATTCAGGCAG
GGAAAATCACCCACGTGTGGGGAGATAAGAAAGACTGTGAAACATGTGATCCGAACTACCAGGTTTTT
CAGTAGAGGGGGGACACGCCCAACCGGTGCCCCCCCGTTCCCGGAAGACAACACCCACGGGAAGGGAA
GGTTGGATTCCTGACTGGATGATCATCGCAGGAGATTACTTCAAAAGGCTACAGGGGAAGGCTAACAT
CATGGGAGAGGGCTTTGACATCATCTTGGATGAAGCTAGGGACCTGTTTCCGTCAAGCAAAGAGTTTG
CCCTACCAGGCTTGATACGACAGTACCTCGAGAGACGGGACGTACCACTGGATTCGGCCTTCTTTCTG
GATGTTTTCCTACCGCTGGTCTTGATGGCCCTTGCCATCACGACCAACCGATGGACTCGAATGGCCCT
GGCCGTTGGGGTGTACCTTACCGGGTACTACTACATAGCCATCATGCTGGCCGCCACCTCGGTGGTGT
CGCTAGCTTTCAAGGCATTCGCACCAAGGAAATGTGAGCGCGGGGACGTTGTGATTGAGAATGGGAGA
CTCAGTGTGCTGGGTATCACCGCGGTAGCCGTGGCTGCCACGGTGGGCATCCACTACTGGCAACCAAG
TCCTTCGCTCATATTTGCGGTGGCCTCATTGGCCGGGTTCGCCGCAATCCTCATGGCTTTACCGACTA
TCCAATACCATGGCGCCACAGACGTCGCTAAGATGATGATGGCGGTCCTCCTATTCGCAGGGGTGATC

TACATCGCGTCCTCTTTTGATGTCGACAAGGACCAGCTGTACTTGTTTGTAAAGACGGGCACCCCGAC
CTACCACATCCCACGTGGAGAGAGAGAAAACGCAGTAAAGCTGGATCAGGTCTACAACCTGGCCCGAT
ACTACGCAAGGTATCCAGCAGATCTGCGTGCCAGATTCGTGACAGAGGAGGCGATATGGGAGTGGGCC
CCCGGCGCCACATGGACGGTGGCTGAGATGGACTACATGGCAACAATTCTCCATGCGATCGTCTTCAG
CCTCGCGGTTTACACCATGTTGACGGATTCAGGGGAAACGGACAGGGTCCTTGGGGACATAAAGATGC
GTATCCTAGAAAAAATCAAGACCACAGAGCTCTTTGGGGATGTGACGATGGCAAAGATAACTCTCGCT
ATCAGCAGGGTTGAGTGGCTTGTCGTCATAGCGGGAAATCTATTGCACACCTACTTTGCCCTAGGGCT
TCCGGGCGTTGCGGTCGAGATCCTCCTGGCAGCCGGACTGATGGTACCCACTTACCACCTGTGGAGCA
GGACCTACCGAGTCATGTCATACCTGAGAGGGACTAATGGCTACAGACAGCCTGTTGAGGGCTTGCTC
CCAGCCCCTACGATAAGGAACACCCAAACGCAGTACACAGCAGGATTGGCAACAATAGTGGGAATCGC
GGCTGTGCTCGTAAACCTGTACTACTACCTGATAGGAAGCAACCCCTATTATGTAGCCCACAGCGTGC
TAGTCTTCGCAGGCACGTTTGTGATGGTCCAGGACAACGACCAGTTGAACGGCTACCCACTCTTAATG
TTGATGGCATACACATTTAACTCCCCAAGTCTGGCACTCATAGGTAGCATCTACAAAAAGTGCCTAGG
TAGAGTCCTGTGGAGCAGGACGACTTAGGGGGTTTCGTTCATACCGGAAAAGAC

>MT297_ON186523
AGTTGCAAGTGCCATAGCTTGGAATCCCGCCGCAGACTCAAACGAACGAAACACTCCAAAAAGCAAAA
CCGGCTTCGCAGCTACTGCTAGAAACAGACGGATTTGTGGGAGTACCAACAACAACAATGCCAAACAC
AAAAACTGCCTTGCTCTTGGTCACCCTGATGGGGTTGGCCTTAGGAGGCAATGACAAGCCAGACTGGG
AAAAGGTGAAGCTGCTCGGACAGCAACTGCAGTCGGGCATTGCTACAAAGAACGCCGTAAAAGTGAAG
TACTACGCTGAACTAATCATCTCTGAGATGCCTACTGACGAAACAACATATTATGCTGGCCTGAGAAC
AGGACAAGAAGTGGCACTCACGGTGGGCTCGGCGAAGTCCATCGTCGGAGAGACTCTGAACTTAAGAA
TCAACCAATGGACTAGGGCAGTCGGGGAGTATCTCATGATCGACCGGCATGTCCCAGGACTGACCCTC
CTGATCACGGGAGAGGATGTCTTGAGTGATCTCATGGAATTCGGGTGGAGCTACAGGCTTAACCCTAG
CGGACACTACCCTGTATACGCAGCCACCAAGACCCTACCCGGGAAGGGGGGCAGGTACACTGGTGGAA
AGACCTACACCGACAGGTATGCCGCGTTCTATGACGGACACGTAAAAGACCACATGGTAGCAATACAC
CCTCACCAGGCTGACATCGAGAGAATGACTAAATACGAACCAATCAACCTAGTCTACGCAGATCTAAC
ACCGAGGAGCATTGGTGGAAGACTCGAGCACGTTCTCGTGACGAAGGGCCACTACACAATTCAGGCAG
GGAAAATCACCCACGTGTGGGGAGATAAGAAAGACTGTGAAACATGTGATCCGAACTACCAGGTTTTT
CAGTAGAGGGGGGACACGCCCAACCGGTGCCCCCCCGTTCCCGGAAGACAACACCCACGGGAAGGGAA
GGTTGGATTCCTGACTGGATGATCATCGCAGGAGATTACTTCAAAAGGCTACAGGGGAAGGCTAACAT
CATGGGAGAGGGCTTTGACATCATCTTGGATGAAGCTAGGGACCTGTTTCCGTCAAGCAAAGAGTTTG
CCCTACCAGGCTTGATACGACAGTACCTCGAGAGACGGGACGTACCACTGGATTCGGCCTTCTTTCTG
GATGTTTTCCTACCGCTGGTCTTGATGGCCCTTGCCATCACGACCAACCGATGGACTCGAATGGCCCT
GGCCGTTGGGGTGTACCTTACCGGGTACTACTACATAGCCATCATGCTGGCCGCCACCTCGGTGGTGT
CGCTAGCTTTCAAGGCATTCGCACCAAGGAAATGTGAGCGCGGGGACGTTGTGATTGAGAATGGGAGA
CTCAGTGTGCTGGGTATCACCGCGGTAGCCGTGGCTGCCACGGTGGGCATCCACTACTGGCAACCAAG
TCCTTCGCTCATATTTGCGGTGGCCTCATTGGCCGGGTTCGCCGCAATCCTCATGGCTTTACCGACTA
TCCAATACCATGGCGCCACAGACGTCGCTAAGATGATGATGGCGGTCCTCCTATTCGCAGGGGTGATC
TACATCGCGTCCTCTTTTGATGTCGACAAGGACCAGCTGTACTTGTTTGTAAAGACGGGCACCCCGAC
CTACCACATCCCACGTGGAGAGAGAGAAAACGCAGTAAAGCTGGATCAGGTCTACAACCTGGCCCGAT
ACTACGCAAGGTATCCAGCAGATCTGCGTGCCAGATTCGTGACAGAGGAGGCGATATGGGAGTGGGCC
CCCGGCGCCACATGGACGGTGGCTGAGATGGACTACATGGCAACAATTCTCCATGCGATCGTCTTCAG
CCTCGCGGTTTACACCATGTTGACGGATTCAGGGGAAACGGACAGGGTCCTTGGGGACATAAAGATGC
GTATCCTAGAAAAAATCAAGACCACAGAGCTCTTTGGGGATGTGACGATGGCAAAGATAACTCTCGCT
ATCAGCAGGGTTGAGTGGCTTGTCGTCATAGCGGGAAATCTATTGCACACCTACTTTGCCCTAGGGCT
TCCGGGCGTTGCGGTCGAGATCCTCCTGGCAGCCGGACTGATGGTACCCACTTACCACCTGTGGAGCA
GGACCTACCGAGTCATGTCATACCTGAGAGGGACTAATGGCTACAGACAGCCTGTTGAGGGCTTGCTC

CCAGCCCCTACGATAAGGAACACCCAAACGCAGTACACAGCAGGATTGGCAACAATAGTGGGAATCGC
GGCTGTGCTCGTAAACCTGTACTACTACCTGATAGGAAGCAACCCCTATTATGTAGCCCACAGCGTGC
TAGTCTTCGCAGGCACGTTTGTGATGGTCCAGGACAACGACCAGTTGAACGGCTACCCACTCTTAATG
TTGATGGCATACACATTTAACTCCCCAAGTCTGGCACTCATAGGTAGCATCTACAAAAAGTGCCTAGG
TAGAGTCCTGTGGAGCAGGACGACTTAGGGGGTTTCGTTCATACCGGAAAAGAC

>MT290_ON186524
AGTTGCAAGTGCCATAGCTTGGAATCCCGCCGCAGACTCAAACGAACGAAACACTCCAAAAAGCAAAA
CCGGCTTCGCAGCTACTGCTAGAAACAGACGGATTTGTGGGAGTACCAACAACAACAATGCCAAACAC
AAAAACTGCCTTGCTCTTGGTCACCCTGATGGGGTTGGCCTTAGGAGGCAATGACAAGCCAGACTGGG
AAAAGGTGAAGCTGCTCGGACAGCAACTGCAGTCGGGCATTGCTACAAAGAACGCCGTAAAAGTGAAG
TACTACGCTGAACTAATCATCTCTGAGATGCCTACTGACGAAACAACATATTATGCTGGCCTGAGAAC
AGGACAAGAAGTGGCACTCACGGTGGGCTCGGCGAAGTCCATCGTCGGAGAGACTCTGAACTTAAGAA
TCAACCAATGGACTAGGGCAGTCGGGGAGTATCTCATGATCGACCGGCATGTCCCAGGACTGACCCTC
CTGATCACGGGAGAGGATGTCTTGAGTGATCTCATGGAATTCGGGTGGAGCTACAGGCTTAACCCTAG
CGGACACTACCCTGTATACGCAGCCACCAAGACCCTACCCGGGAAGGGGGGCAGGTACACTGGTGGAA
AGACCTACACCGACAGGTATGCCGCGTTCTATGACGGACACGTAAAAGACCACATGGTAGCAATACAC
CCTCACCAGGCTGACATCGAGAGAATGACTAAATACGAACCAATCAACCTAGTCTACGCAGATCTAAC
ACCGAGGAGCATTGGTGGAAGACTCGAGCACGTTCTCGTGACGAAGGGCCACTACACAATTCAGGCAG
GGAAAATCACCCACGTGTGGGGAGATAAGAAAGACTGTGAAACATGTGATCCGAACTACCAGGTTTTT
CAGTAGAGGGGGGACACGCCCAACCGGTGCCCCCCCGTTCCCGGAAGACAACACCCACGGGAAGGGAA
GGTTGGATTCCTGACTGGATGATCATCGCAGGAGATTACTTCAAAAGGCTACAGGGGAAGGCTAACAT
CATGGGAGAGGGCTTTGACATCATCTTGGATGAAGCTAGGGACCTGTTTCCGTCAAGCAAAGAGTTTG
CCCTACCAGGCTTGATACGACAGTACCTCGAGAGACGGGACGTACCACTGGATTCGGCCTTCTTTCTG
GATGTTTTCCTACCGCTGGTCTTGATGGCCCTTGCCATCACGACCAACCGATGGACTCGAATGGCCCT
GGCCGTTGGGGTGTACCTTACCGGGTACTACTACATAGCCATCATGCTGGCCGCCACCTCGGTGGTGT
CGCTAGCTTTCAAGGCATTCGCACCAAGGAAATGTGAGCGCGGGGACGTTGTGATTGAGAATGGGAGA
CTCAGTGTGCTGGGTATCACCGCGGTAGCCGTGGCTGCCACGGTGGGCATCCACTACTGGCAACCAAG
TCCTTCGCTCATATTTGCGGTGGCCTCATTGGCCGGGTTCGCCGCAATCCTCATGGCTTTACCGACTA
TCCAATACCATGGCGCCACAGACGTCGCTAAGATGATGATGGCGGTCCTCCTATTCGCAGGGGTGATC
TACATCGCGTCCTCTTTTGATGTCGACAAGGACCAGCTGTACTTGTTTGTAAAGACGGGCACCCCGAC
CTACCACATCCCACGTGGAGAGAGAGAGAAAACGCAGTAAAGCTGGATCAGGTCTACAACCTGGCCCGAT
ACTACGCAAGGTATCCAGCAGATCTGCGTGCCAGATTCGTGACAGAGGAGGCGATATGGGAGTGGGCC
CCCGGCGCCACATGGACGGTGGCTGAGATGGACTACATGGCAACAATTCTCCATGCGATCGTCTTCAG
CCTCGCGGTTTACACCATGTTGACGGATTCAGGGGAAACGGACAGGGTCCTTGGGGACATAAAGATGC
GTATCCTAGAAAAAATCAAGACCACAGAGCTCTTTGGGGATGTGACGATGGCAAAGATAACTCTCGCT
ATCAGCAGGGTTGAGTGGCTTGTCGTCATAGCGGGAAATCTATTGCACACCTACTTTGCCCTAGGGCT
TCCGGGCGTTGCGGTCGAGATCCTCCTGGCAGCCGGACTGATGGTACCCACTTACCACCTGTGGAGCA
GGACCTACCGAGTCATGTCATACCTGAGAGGGACTAATGGCTACAGACAGCCTGTTGAGGGCTTGCTC
CCAGCCCCTACGATAAGGAACACCCAAACGCAGTACACAGCAGGATTGGCAACAATAGTGGGAATCGC
GGCTGTGCTCGTAAACCTGTACTACTACCTGATAGGAAGCAACCCCTATTATGTAGCCCACAGCGTGC
TAGTCTTCGCAGGCACGTTTGTGATGGTCCAGGACAACGACCAGTTGAACGGCTACCCACTCTTAATG
TTGATGGCATACACATTTAACTCCCCAAGTCTGGCACTCATAGGTAGCATCTACAAAAAGTGCCTAGG
TAGAGTCCTGTGGAGCAGGACGACTTAGGGGGTTTCGTTCATACCGGAAAAGAC

>MT299_ON186525
AGTTGCAAGTGCCATAGCTTGGAATCCCGCCGCAGACTCAAACGAACGAAACACTCCAAAAAGCAAAA
CCGGCTTCGCAGCTACTGCTAGAAACAGACGGATTTGTGGGAGTACCAACAACAACAATGCCAAACAC

AAAAACTGCCTTGCTCTTGGTCACCCTGATGGGGTTGGCCTTAGGAGGCAATGATAAGCCAGACTGGG
AAAAGGTGAAGCTGCTCGGACAGCAACTGCAGTCGGGCATTGCTACAAAGAACGCCGTAAAAGTGAAG
TACTACGCTGAACTAATCATCTCTGAGATGCCTACTGACGAAACAACATATTATGCTGGCCTGAGAAC
AGGACAAGAAGTGGCACTCACGGTGGGCTCGGCGAAGTCCATCGTCGGAGAGACTCTGAACTTAAGAA
TCAACCAATGGACTAGGGCAGTCGGGGAGTATCTCATGATCGACCGGCATGTCCCAGGACTGACCCTC
CTGATCACGGGAGAGGATGTCTTGAGTGATCTCATGGAATTCGGGTGGAGCTACAGGCTCAACCCTAG
CGGACATTACCCTGTATACGCAGCCACTAAGACCCTACCCGGGAAGGGGGGCAGGTACACTGGTGGAA
AGACCTACACCGACAGGTATGCCGCGTTCTATGACGGACACGTAAAAGACCACATGGTAGCAATACAC
CCTCACCAGGCTGACATCGAGAGAATGACTAAATACGAACCAATCAACCTAGTCTACGCAGATCTAAC
ACCGAGGAGCATTGGTGGAAGACTCGAGCACGTTCTCGTGACGAAGGGCCACTACACAATTCAGGCAG
GGAAAATCACCCACGTGTGGGGAGATAAGAAAGACTGTGAAACATGTGATCCGAACTACCAGGTTTTT
CAGTAGAGGGGGGACACGCCCAACCGGTGCCCCCCCGTTCCCGGAAGACAACACCCACGGGAAGGGAA
GGTTGGATTCCTGACTGGATGATCATCGCAAGAGATTACTTCAAAAGGCTACAGGGAAAGGCTGACAT
CATGGGAGAGGGCTTTGACATCATCTTGGATGAAGCTAGGGACCTGTTTCCGTCAAGCAAAGAGTTTG
CCCTACCAGGCTTGATACGACAGTACCTCGAGAGACGGGACGTACCACTGGATTCGGCCTTCTTTCTG
GATGTTTTTCTACCGCTGGTCTTGATGGCCCTTGCCATCACGACTAACCGATGGACTCGAATGGCCCT
GGCCGTTGGGGTGTACCTTACCGGGTACTACTACATAGCCATCATGCTGGCCGCCACCTCGGTGGTGT
CGCTAGCTTTCAAGGCATTCGCACCAAGGAAATGTGAGCGCGGGGACGTTGTGATCGAGAATGGGAGA
CTCAGTGTGCTGGGCATCACCGCGGTAGCCGTGGCTGCCACGGTGGGCATCCACTACTGGCAACCAAG
TCCTTCGCTCATATTTGCGGTGGCCTCATTGGCCGGGTTCGCCGCAATCCTCATGGCTTTACCGACCA
TCCAATACCATGGCGCCACAGACGTCGCTAAGATGATGATGGCGGTCCTCCTATTCGCAGGGGTGATC
TACATCGCGTCCTCTTTTGATGTCGACAAGGACCAGCTATACTTGTTTGTGAAGACAGGCACCCCGAC
CTACCACATCCCACGTGGAGAGAGAGAAAACGCAGTAAAGCTGGATCAGGTCTACAACCTGGCCCGAT
ACTACGCAAGGTATCCAGCTGATCTGCGTGCCAGATTCGTGACAGAAGAGGCGATATGGGAGTGGGCC
CCCGGCGCCACATGGACGGTGGCTGAGATGGACTACATGGCAACAATTCTCCATGCGATCGTCTTCAG
CCTCGCGGTTTACACCATGTTGACGGATTCAGGGGAAACGGACAGGGTCCTTGGGGACATAAAGATGC
GTATCCTAGAAAAAATCAAGACCACAGAGCTCTTTGGGGATGTGACGATGGCAAAGATAACTCTCGCT
ATCAGCAGGGTTGAGTGGCTTGTCGTCATAGCGGGAAATCTATTGCACACCTACTTTGCCCTGGGGCT
TCCGGGCGTTGCGGTCGAGATCCTCCTGGCAGCCGGACTGATGGTACCCACTTACCACCTGTGGAGCA
GGACCTACCGAGTCATGTCATACCTGAGAGGGACTAATGGCTACAGACAGCCTGTTGAGGGCCTGCTC
CCAGCCCCTACGATAAGGAACACCCAAACGCAGTACACAGCAGGATTGGCAACAATAGTGGGAATCGC
GGCTGTGCTCGTAAACCTGTACTACTACCTGATAGGAAGCAACCCCTATTATGTAGCCCACAGCGTGC
TAGTCTTCGCAGGCACGTTTGTGATGGTCCAGGACAACGACCAGTTGAACGGCTACCCACTCTTAATG
TTGATGGCATACACATTTAACTCCCCAAGTCTGGCACTCATAGGTAGCATCTACAAAAAGTGCCTAGG
CAGAGTCCTGTGGAGCAGGACGACTTAGGGGGTTTCGTTCATACCGGAAAAGAC

>MT293_ON186526
AGTTGCAAGTGCCATAGCTTGGAATCCCGCCGCAGACTCAAACGAACGAAACACTCCAAAAAGCAAAA
CCGGCTTCGCAGCTACTGCTAGAAACAGACGGATTTGTGGGAGTACCAACAACAACAATGCCAAACAC
AAAAACTGCCTTGCTCTTGGTCACCCTGATGGGGTTGGCCTTAGGAGGCAATGATAAGCCAGACTGGG
AAAAGGTGAAGCTGCTCGGACAGCAACTGCAGTCGGGCATTGCTACAAAGAACGCCGTAAAAGTGAAG
TACTACGCTGAACTAATCATCTCTGAGATGCCTACTGACGAAACAACATATTATGCTGGCCTGAGAAC
AGGACAAGAAGTGGCACTCACGGTGGGCTCGGCGAAGTCCATCGTCGGAGAGACTCTGAACTTAAGAA
TCAACCAATGGACTAGGGCAGTCGGGGAGTATCTCATGATCGACCGGCATGTCCCAGGACTGACCCTC
CTGATCACGGGAGAGGATGTCTTGAGTGATCTCATGGAATTCGGGTGGAGCTACAGGCTCAACCCTAG
CGGACATTACCCTGTATACGCAGCCACTAAGACCCTACCCGGGAAGGGGGGCAGGTACACTGGTGGAA
AGACCTACACCGACAGGTATGCCGCGTTCTATGACGGACACGTAAAAGACCACATGGTAGCAATACAC
CCTCACCAGGCTGACATCGAGAGAATGACTAAATACGAACCAATCAACCTAGTCTACGCAGATCTAAC

ACCGAGGAGCATTGGTGGAAGACTCGAGCACGTTCTCGTGACGAAGGGCCACTACACAATTCAGGCAG
GGAAAATCACCCACGTGTGGGGAGATAAGAAAGACTGTGAAACATGTGATCCGAACTACCAGGTTTTT
CAGTAGAGGGGGGACACGCCCAACCGGTGCCCCCCCGTTCCCGGAAGACAACACCCACGGGAAGGGAA
GGTTGGATTCCTGACTGGATGATCATCGCAAGAGATTACTTCAAAAGGCTACAGGGAAAGGCTGACAT
CATGGGAGAGGGCTTTGACATCATCTTGGATGAAGCTAGGGACCTGTTTCCGTCAAGCAAAGAGTTTG
CCCTACCAGGCTTGATACGACAGTACCTCGAGAGACGGGACGTACCACTGGATTCGGCCTTCTTTCTG
GATGTTTTTCTACCGCTGGTCTTGATGGCCCTTGCCATCACGACTAACCGATGGACTCGAATGGCCCT
GGCCGTTGGGGTGTACCTTACCGGGTACTACTACATAGCCATCATGCTGGCCGCCACCTCGGTGGTGT
CGCTAGCTTTCAAGGCATTCGCACCAAGGAAATGTGAGCGCGGGGACGTTGTGATCGAGAATGGGAGA
CTCAGTGTGCTGGGCATCACCGCGGTAGCCGTGGCTGCCACGGTGGGCATCCACTACTGGCAACCAAG
TCCTTCGCTCATATTTGCGGTGGCCTCATTGGCCGGGTTCGCCGCAATCCTCATGGCTTTACCGACCA
TCCAATACCATGGCGCCACAGACGTCGCTAAGATGATGATGGCGGTCCTCCTATTCGCAGGGGTGATC
TACATCGCGTCCTCTTTTGATGTCGACAAGGACCAGCTATACTTGTTTGTGAAGACAGGCACCCCGAC
CTACCACATCCCACGTGGAGAGAGAGAAAACGCAGTAAAGCTGGATCAGGTCTACAACCTGGCCCGAT
ACTACGCAAGGTATCCAGCTGATCTGCGTGCCAGATTCGTGACAGAAGAGGCGATATGGGAGTGGGCC
CCCGGCGCCACATGGACGGTGGCTGAGATGGACTACATGGCAACAATTCTCCATGCGATCGTCTTCAG
CCTCGCGGTTTACACCATGTTGACGGATTCAGGGGAAACGGACAGGGTCCTTGGGGACATAAAGATGC
GTATCCTAGAAAAAATCAAGACCACAGAGCTCTTTGGGGATGTGACGATGGCAAAGATAACTCTCGCT
ATCAGCAGGGTTGAGTGGCTTGTCGTCATAGCGGGAAATCTATTGCACACCTACTTTGCCCTGGGGCT
TCCGGGCGTTGCGGTCGAGATCCTCCTGGCAGCCGGACTGATGGTACCCACTTACCACCTGTGGAGCA
GGACCTACCGAGTCATGTCATACCTGAGAGGGACTAATGGCTACAGACAGCCTGTTGAGGGCCTGCTC
CCAGCCCCTACGATAAGGAACACCCAAACGCAGTACACAGCAGGATTGGCAACAATAGTGGGAATCGC
GGCTGTGCTCGTAAACCTGTACTACTACCTGATAGGAAGCAACCCCTATTATGTAGCCCACAGCGTGC
TAGTCTTCGCAGGCACGTTTGTGATGGTCCAGGACAACGACCAGTTGAACGGCTACCCACTCTTAATG
TTGATGGCATACACATTTAACTCCCCAAGTCTGGCACTCATAGGTAGCATCTACAAAAAGTGCCTAGG
CAGAGTCCTGTGGAGCAGGACGACTTAGGGGGTTTCGTTCATACCGGAAAAGAC
