# Supplementary material for: Jingmen Tick Virus in Ticks from Kenya
Source: Viruses. 2022 May 13;14(5):1041. doi: 10.3390/v14051041 (PMC9147648; doi:10.3390/v14051041)
Supplement: Supplementary file 1 [file viruses-14-01041-s001.zip › viruses-1707077-supplementary/Table S3.pdf]

Table S3. Relative abundance of ticks sampled from different hosts.

Tick Species	Goats (%)	Sheep (%)	Cattle (%)	Tortoises (%)	n (%)
Rhipicephalus appendiculatus	1190 (26.15)	979 (21.52)	205 (4.51)	0	2374 (52.18)
Hyalomma rufipes	373 (8.20)	31 (0.68)	369 (8.11)	0	773 (17.00)
Hyalomma impeltatum	381 (8.37)	435 (9.56)	168 (3.69)	0	984 (21.62)
Rhipicephalus evertsi evertsi	22 (0.48)	65 (1.43)	16 (0.35)	0	103 (2.26)
Amblyomma gemma	22 (0.48)	9 (0.20)	39 (0.86)	0	70 (1.54)
Hyalomma albiparmatum	27 (0.59)	11 (0.24)	23 (0.51)	0	61 (1.34)
Hyalomma truncatum	26 (0.57)	19 (0.42)	17 (0.37)	0	62 (1.36)
Amblyomma sp.	0	0	0	35 (0.77)	35 (0.77)
Amblyomma sparsum	0	0	0	7 (0.15)	7 (0.15)
Amblyomma nuttalli	0	0	0	5 (0.11)	5 (0.11)
Rhipicephalus pulchellus	0	0	3 (0.07)	0	3 (0.07)
Amblyomma variegatum	0	11 (0.24)	0	0	11 (0.24)
Amblyomma lepidum	0	0	4 (0.09)	0	4 (0.09)
Hyalomma marginatum	0	2 (0.04)	0	0	2 (0.04)
Hyalomma marginatum spp.	29 (0.64)	12 (0.26)	15 (0.33)	0	56 (1.23)
**Total (%)**	**2070 (45.49)**	**1574 (34.59)**	**859 (18.88)**	**47 (1.03)**	**4550**

n: number of collected ticks.
