# Supplementary figures and images for: Jingmen Tick Virus in Ticks from Kenya
Source: Viruses. 2022 May 13;14(5):1041. doi: 10.3390/v14051041 (PMC9147648; doi:10.3390/v14051041)

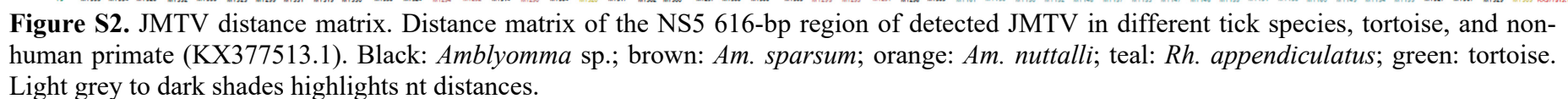

Supplement: Supplementary file 1 [file viruses-14-01041-s001.zip › viruses-1707077-supplementary/Figure S2.pdf]
